# Supplementary material for: Selection of isomerization pathways of multistep photoswitches by chalcogen bonding
Source: Nat Commun. 2023 Nov 6;14:7139. doi: 10.1038/s41467-023-43013-8 (PMC10628202; doi:10.1038/s41467-023-43013-8)
Supplement: Supplementary file 1 — Supplementary Information [file 41467_2023_43013_MOESM1_ESM.pdf]

Supplementary Information

Selection of Isomerization Pathways of Multistep Photoswitches by Chalcogen Bonding

Shuaipeng Jia,[a,b] Hebo Ye,[a] Peng He,[a] Xin Lin,[a,b] and Lei You[a,b,c*]

a. State Key Laboratory of Structural Chemistry, Fujian Institute of Research on the Structure of Matter, Chinese Academy of Sciences, Fuzhou 350002, China.

b. University of Chinese Academy of Sciences, Chinese Academy of Sciences, Beijing 100049, China.

c. Fujian Science & Technology Innovation Laboratory for Optoelectronic Information of China, Fuzhou 350108, China.

*e-mail: lyou@fjirsm.ac.cn

Supplementary Table of Contents

Supplementary Note 1. General Methods……………..S3

Supplementary Note 2. Synthesis and Characterization................................S4-S38

Supplementary Note 3. DFT Calculations……………...................................S39-S44

Supplementary Note 4. Photoswitching Studies of Neutral Compounds........S45-S74

(1) Photoswitching Experiments……………..............................………......S45-S62

(2) Thermal Experiments.……..…........................……….........................S63-S72

Supplementary Note 5. Photoswitching Studies of Cationic Telluronium........S73-S80

Supplementary Note 6. Dynamic Covalent Chemistry Gated Photoswitching.S81-S90

Supplementary Note 7. Photoaddressed Rewritable Materials……......….............S91-S98

Supplementary Note 8. References………...S99

Supplementary Note 1. General Methods

General. ^{1}H NMR and ^{13}C NMR spectra were recorded on a 400 MHz Bruker Biospin Avance III spectrometer or a 400 MHz JEOL JNM-ECZ400S spectrometer. The chemical shifts (δ) for ^{1}H NMR spectra, given in ppm, are referenced to the residual proton signal of the deuterated solvent. Mass spectral analysis (ESI-HRMS) was performed on a Bruker IMPACT-II spectrometer. Crystallographic data was collected on a Mercury single crystal diffractometer at room temperature. The structures were solved with direct methods by using OlexSys or SHELXS-97 and refined with the full-matrix least-squares technique based on F2. The UV-vis spectra were measured on a PerkinElmer Lambda 365 spectrometer. All other reagents were obtained from commercial sources and were used without further purification, unless indicated otherwise.

Supplementary Note 2. Synthesis and Characterization

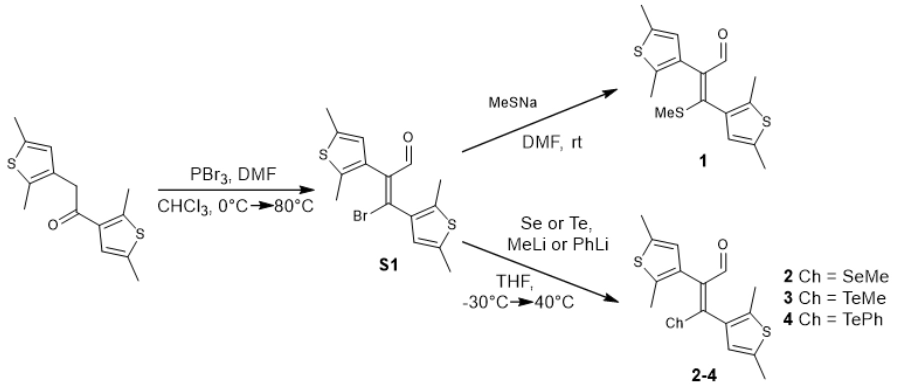

Supplementary Figure 1. General synthetic routes of **1-4**.

S1: A flame-dried round bottom flask containing a stir bar, $CHCl_3$ (20.0 ml), and DMF (2.3 g, 32 mmol) was cooled to 0 ℃, and PBr_3 (3.0 mL, 32 mmol) was added dropwise while stirring. The mixture was warmed to ambient temperature for 30 min, and then cooled to 0 ℃ before the slow addition of 1,2-bis(2,5-dimethylthiophen-3-yl)ethenone[1] (2.1 g, 8.0 mmol). The reaction mixture was stirred for 1 h at 0 ℃, heated to 80 ℃ for 5 h, then cooled to ambient temperature, and finally poured into an ice-cold saturated $NaHCO_3$ solution. Once neutralized, the mixture was extracted with dichloromethane and washed with water and brine. The organic layer was dried over anhydrous Na_2SO_4, evaporated, and then purified by column chromatography (SiO_2, petroleum ether/ethyl acetate 20:1 to 10:1) to give *E*-**S1** (1.27 g, 45%) as a light-yellow solid and *Z*-**S1** (0.56 g, 20%) as a brown solid. *E*-**S1**: ^{1}H NMR (400 MHz, 25 ℃, $CDCl_3$): δ 9.46 (s, 1H), 6.77 (s, 1H), 6.45 (s, 1H), 2.43 (s, 3H), 2.43 (s, 3H), 2.37 (s, 3H), 2.28 (s, 3H). ^{13}C NMR (100 MHz, 25 ℃, $CDCl_3$): δ 189.0, 144.0, 140.7, 139.1, 137.8, 136.4, 135.8, 134.7, 131.8, 126.9, 126.5, 15.6, 15.5, 14.8, 14.4. ESI-HRMS: m/z calcd for $C_{15}H_{16}BrONaS_2$ $[M + Na]^+$: 376.9645; found: 376.9642. *Z*-**S1**: ^{1}H NMR (400 MHz, 25 ℃, $CDCl_3$): δ 10.32 (s, 1H), 6.59 (s, 1H), 6.18 (s, 1H), 2.35(s, 3H), 2.31(s, 3H), 1.95(s, 3H), 1.93(s, 3H). ^{13}C NMR (100 MHz, 25 ℃, $CDCl_3$): δ 193.7, 138.0, 137.8, 136.6, 136.1, 136.0, 135.7, 135.4, 130.1, 126.9, 126.8, 15.5, 15.4, 14.8, 14.8. ESI-HRMS: m/z calcd for $C_{15}H_{16}BrONaS_2$ $[M + Na]^+$: 376.9645; found: 376.9642.

E-**1:** MeSNa (20wt% in water, 0.42 g, 1.2 mmol) was added to a solution of **S1** (0.31 g, 1.0 mmol) in anhydrous DMF (10 mL) under N_2 atmosphere. After stirring at 40 ℃ for 3 h, the solvent was removed under reduced pressure, and the residue was extracted with ethyl acetate. The combined organic layer was dried over anhydrous Na_2SO_4. The crude product was purified by column chromatography (SiO_2, petroleum ether/ethyl acetate 30:1) to give *E*-**1** (0.28 g, 85%) as a white solid. ^{1}H NMR (400 MHz, 25 ℃, $CDCl_3$): δ 9.30 (s, 1H), 6.56 (s, 1H), 6.46 (s, 1H), 2.45 (s, 3H), 2.43 (s, 3H), 2.34 (s, 3H), 2.26 (s, 3H), 1.57 (s, 3H). ^{13}C NMR (100 MHz, 25 ℃, $CDCl_3$): δ 188.8, 163.5, 138.2, 136.6, 135.9, 135.6, 133.9, 130.7, 130.5, 126.7, 126.7, 15.7, 15.5, 15.1, 14.4, 13.8. ESI-HRMS: m/z calcd for $C_{16}H_{18}ONaS_3$ $[M + Na]^+$: 345.0417; found: 375.0412.

E-**2:** Selenium (0.16 g, 2.0 mmol) was suspended in degassed anhydrous THF (20 mL) under N_2 atmosphere. The suspension was cooled to -30 ℃, and MeLi (1.7 M, 1.3 mL, 2.2 mmol) was added dropwise. The resulting white suspension was stirred for 40 min. *E*-**S1** (0.85 g, 2.4 mmol) in degassed anhydrous THF (10 mL) was added dropwise to give a clear yellow solution. The mixture was then stirred at 40 ℃ for 4 h, quenched with water, and extracted with ethyl acetate. The organic layer was washed with brine, dried over anhydrous Na_2SO_4, and concentrated *in vacuo*. The crude mixture was purified by column chromatography (SiO_2, petroleum ether/ethyl acetate 30:1) to give *E*-**2** as a yellow solid (0.54 g, 73%). ^{1}H NMR (400 MHz, 25 ℃, $CDCl_3$): δ 9.30 (s, 1H), 6.54 (s, 1H), 6.47 (s, 1H), 2.45 (s, 3H), 2.43 (s, 3H), 2.33 (s, 3H), 2.28 (s, 3H), 1.67 (s, 3H). ^{13}C NMR (100 MHz, 25 ℃, $CDCl_3$): δ 188.0, 163.6, 137.9, 137.2, 136.9, 135.6, 135.1, 132.0, 131.9, 15.7, 15.5, 14.3, 13.7, 6.9. ESI-HRMS: m/z calcd for $C_{16}H_{18}ONaS_2Se$ $[M + Na]^+$: 392.9862; found: 392.9856.

3: Tellurium (0.38 g, 3.0 mmol) was suspended in degassed anhydrous THF (20 mL) under N_2 atmosphere. The suspension was cooled to -30 ℃, and MeLi (1.7 M, 1.95 mL, 3.3 mmol) was added dropwise. The resulting white suspension was stirred for 40 min. *E*-**S1** (1.17 g, 3.3 mmol) in degassed anhydrous THF (15 mL) was added dropwise to give a clear orange solution. The mixture was then stirred at 40 ℃ for 4 h, quenched with water, and extracted with ethyl acetate. The organic layer was washed with brine, dried over anhydrous Na_2SO_4,

and concentrated *in vacuo*. The crude mixture was purified by column chromatography (SiO_2, petroleum ether/ethyl acetate 60:1 to 30:1) to give *E*-**3** as a yellow solid (0.36 g, 29%) and *Z*-**3** as an orange liquid (0.44 g, 35%). *E*-**3**: ^{1}H NMR (400 MHz, 25 ℃, $CDCl_3$): δ 9.27 (s, 1H), 6.54 (s, 1H), 6.45 (s, 1H), 2.44 (s, 3H), 2.44 (s, 3H), 2.31 (s, 3H), 2.31 (s, 3H), 1.43 (s, 3H). ^{13}C NMR (100 MHz, 25 ℃, $CDCl_3$): δ 186.6, 155.5, 143.6, 137.5, 137.4, 135.4, 134.4, 134.4, 133.2, 126.5, 125.7, 15.7, 15.5, 14.2, 13.8, -13.8. ESI-HRMS: m/z calcd for $C_{16}H_{18}ONaS_2Te$ $[M + Na]^+$: 442.9759; found: 442.9754.

Z-**3**: ^{1}H NMR (400 MHz, 25 ℃, $CDCl_3$): δ 9.82 (s, 1H), 6.28 (s, 1H), 6.14 (s, 1H), 2.34 (s, 3H), 2.29 (s, 3H), 2.18 (s, 3H), 2.00 (s, 3H), 1.33 (s, 3H). ^{13}C NMR (100 MHz, 25 ℃, $CDCl_3$): δ 191.2, 151,6, 136.9, 136.6, 136.1, 135.6, 135.5, 133.1, 15.5, 14.2, 13.9, -11.5. ESI-HRMS: m/z calcd for $C_{16}H_{18}ONaS_2Te$ $[M + Na]^+$: 442.9759; found: 442.9754.

4: Tellurium (0.38 g, 3.0 mmol) was suspended in degassed anhydrous THF (20 mL) under N_2 atmosphere. The suspension was cooled to -30 ℃, and PhLi (1.0 M, 3.3 mL, 3.3 mmol) was added dropwise. The resulting white suspension was stirred for 40 min. *E*-**S1** (1.17 g, 3.3 mmol) in degassed anhydrous THF (15 mL) was added dropwise to give a clear orange solution. The mixture was then stirred at 40 ℃ for 4 h, quenched with water, and extracted with ethyl acetate. The organic layer was washed with brine, dried over anhydrous Na_2SO_4, and concentrated *in vacuo*. The crude mixture was purified by column chromatography (SiO_2, petroleum ether/ethyl acetate 60:1 to 30:1) to give *E*-**4** as a yellow solid (0.25 g, 17%) and *Z*-**4** as an orange liquid (0.58 g, 40%). *E*-**4**: ^{1}H NMR (400 MHz, 25 ℃, DMSO-d_6): δ 9.16 (s, 1H), 7.42 (d, *J* = 7.2 Hz, 2H), 7.28 (t, *J* = 7.2 Hz, 1H), 7.14 (t, *J* = 7.6 Hz, 2H), 6.53 (s, 1H), 6.28 (s, 1H), 2.43 (s, 3H), 2.30 (s, 3H), 2.14 (s, 3H), 2.10 (s, 3H). ^{13}C NMR (100 MHz, 25 ℃, $CDCl_3$): δ 186.9, 158.7, 142.6, 140.9, 137.6, 136.2, 135.7, 134.5, 134.0, 133.5, 128.9, 128.8, 127.2, 125.7, 116.0, 15.7, 15.0, 14.3, 14.0. ESI-HRMS: m/z calcd for $C_{21}H_{20}ONaS_2Te$ $[M + Na]^+$: 504.9915; found: 504.9910.

Z-**4**: ^{1}H NMR (400 MHz, 25 ℃, $CDCl_3$): δ 9.87 (s, 1H), 7.41 (d, *J* = 6.8 Hz, 2H), 7.17 (t, *J* = 7.2 Hz, 1H), 7.05 (t, *J* = 7.6 Hz, 2H), 6.17 (s, 1H), 5.86 (s, 1H), 2.28 (s, 3H), 2.16 (s, 3H), 1.94 (s, 3H), 1.87 (s, 3H). ^{13}C NMR (100 MHz, 25 ℃, $CDCl_3$): δ 191.8, 154.5, 140.0, 136.4, 135.6, 135.4, 135.4, 134.7, 133.5, 131.7, 128.3, 128.1, 127.6, 126.9, 122.3, 15.5,

14.9, 14.3, 14.1. ESI-HRMS: m/z calcd for $C_{21}H_{20}ONaS_2Te$ $[M + Na]^+$: 504.9915; found: 504.9910.

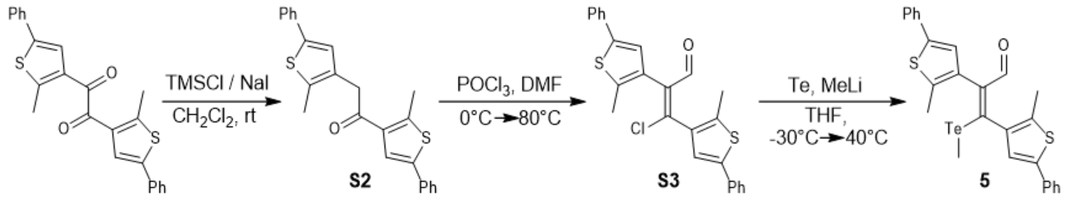

Supplementary Figure 2. Synthesis of **5**.

S2: Under N_2 atmosphere 1,2-bis-(2'-methyl-5'-phenylthien-3'-yl)-ethanedione[2] (4.0 g, 10 mmol) and NaI (7.5 g, 50 mmol) were mixed in CH_2Cl_2 (20 mL). The resulting mixture was stirred for 5 min at room temperature, and TMSCl (5.4 g, 50 mmol) was added to the solution. After stirring for 6 h the dark mixture was washed with saturated $Na_2S_2O_3$ solution (30 mL), extracted with CH_2Cl_2, dried over anhydrous Na_2SO_4, and concentrated *in vacuo*. The crude product was purified by column chromatography (SiO_2, petroleum ether/ethyl acetate 50:1) to give **S2** as a white solid (3.57 g, 92%). ^{1}H NMR (400 MHz, 25 ℃, $CDCl_3$): δ 7.61 (s, 1H), 7.58-7.53 (m, 4H), 7.41 (t, *J* = 7.6 Hz, 2H), 7.36-7.30 (m, 3H), 7.23 (t, *J* = 7.6 Hz, 1H), 7.10 (s, 1H), 4.10 (s, 2H), 2.78 (s, 3H), 2.44 (s, 3H). ^{13}C NMR (100 MHz, 25 ℃, $CDCl_3$): δ 193.2, 150.0, 140.4, 140.0, 136.2, 135.4, 134.7, 133.8, 131.3, 129.4, 129.1, 128.2, 127.3, 126.0, 125.8, 125.6, 123.8, 41.7, 16.8, 13.9. ESI-HRMS: m/z calcd for $C_{24}H_{20}ONaS_2$ $[M + Na]^+$: 411.0853; found: 411.0850.

S3: A flame-dried round bottom flask containing a stir bar and DMF (7.0 mL) was cooled to 0 ℃, and $POCl_3$ (1.9 mL, 20 mmol) was added dropwise while stirring. The mixture was warmed to ambient temperature for 30 min, and then cooled to 0 °C before the slow addition of **S2** (1.9 g, 5.0 mmol). The reaction mixture was stirred for 1 h at 0 ℃, then heated to 80 ℃ for 5h, cooled to ambient temperature, and finally poured into an ice-cold saturated $NaHCO_3$ solution. Once neutralized, the mixture was extracted with dichloromethane and washed with water and brine. The organic layer was dried over anhydrous Na_2SO_4, evaporated, and then purified by column chromatography (SiO_2, petroleum ether/ethyl acetate 10:1) to give *E*-**S3** (1.41 g, 65%) as a pale pink solid. ^{1}H

NMR (400 MHz, 25 ℃, $CDCl_3$): δ 9.64 (s, 1H), 7.59 (m, *J* = 7.2 Hz, 4H), 7.44-7.32 (m, 6H), 7.26 (t, *J* = 7.2 Hz, 1H), 7.08 (s, 1H), 2.53 (s, 3H), 2.40 (s, 3H). ^{13}C NMR (100 MHz, 25 ℃, $CDCl_3$): δ 189.5, 150.5, 142.4, 141.3, 140.9, 138.3, 137.5, 134.5, 133.4, 131.0, 129.4, 129.1, 128.5, 127.6, 126.0, 125.9, 124.9, 124.3, 15.1, 14.8. ESI-HRMS: m/z calcd for $C_{25}H_{19}ClONaS_2$ $[M + Na]^+$: 457.0464; found: 457.0460.

5: Tellurium (0.19 g, 1.5 mmol) was suspended in degassed anhydrous THF (20 mL) under N_2 atmosphere. The suspension was cooled to -30 ℃, and MeLi (1.7 M, 0.97 mL, 1.65 mmol) was added dropwise. The resulting white suspension was stirred for 40 min. *E*-**S3** (0.77 g, 1.52 mmol) in degassed anhydrous THF (10 mL) was added dropwise to give a clear orange solution. The mixture was then stirred at 40 ℃ for 4 h, quenched with water, and extracted with ethyl acetate. The organic layer was washed with brine, dried over anhydrous Na_2SO_4, and concentrated *in vacuo*. The crude mixture was purified by column chromatography (SiO_2, petroleum ether/ethyl acetate 60:1 to 10:1) to give *E*-**5** as a yellow solid (0.23 g, 28%) and *Z*-**5** as an orange solid (0.22 g, 27%). *E*-**5**: ^1H NMR (400 MHz, 25 ℃, DMSO-d_6): δ 9.25 (s, 1H), 7.69 (d, *J* = 7.2 Hz, 2H), 7.62 (d, *J* = 7.2 Hz, 2H), 7.45-7.40 (m, 5H), 7.34-7.28 (m, 2H), 7.26 (s, 1H), 2.42 (s, 3H), 2.37 (s, 3H), 1.48 (s, 3H). ^{13}C NMR (100 MHz, 25 ℃, $CDCl_3$): δ 186.0, 155.3, 143.5, 142.0, 141.7, 137.5, 136.0, 135.6, 135.0, 134.5, 133.8, 129.3, 129.1, 128.1, 127.5, 125.8, 125.7, 123.9, 123.8, 14.5, 14.1, -13.2. ESI-HRMS: m/z calcd for $C_{26}H_{22}ONaS_2Te$ $[M + Na]^+$: 567.0072; found: 567.0067. *Z*-**5**: ^1H NMR (400 MHz, 25 ℃, $CDCl_3$): δ 9.97 (s, 1H), 7.52 (d, *J* = 7.2 Hz, 2H), 7.38-7.22 (m, 8H), 6.97 (s, 1H), 6.79 (s, 1H), 2.34 (s, 3H), 2.14 (s, 3H), 1.46 (s, 3H). ^{13}C NMR (100 MHz, 25 ℃, $CDCl_3$): δ 190.8, 151.5, 141.0, 140.3, 138.2, 136.7, 136.5, 135.4, 134.5, 134.2, 133.6, 129.3, 129.1, 127.8, 127.5, 124.9, 124.2, 14.5, 14.2, -11.0. ESI-HRMS: m/z calcd for $C_{26}H_{22}ONaS_2Te$ $[M + Na]^+$: 567.0072; found: 567.0067.

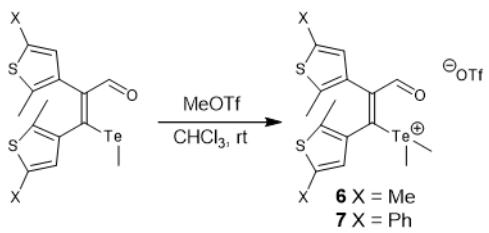

Supplementary Figure 3. Synthesis of **6**-**7**.

6: MeOTf (47 mg, 0.29 mmol) and *Z*-**3** (100 mg, 0.24 mmol) were dissolved and stirred in freshly distilled $CHCl_3$ (6 mL) under N_2 atmosphere. The mixture was stirred overnight in the dark at room temperature. The mixture was concentrated *in vacuo,* and then *n*-hexane (10 mL) was slowly added. The solid precipitates were collected by filtration and washed with *n*-hexane to give *Z*-**6** as a light-yellow solid (138 mg, 99 %). *Z*-**6**: ^{1}H NMR (400 MHz, 25 ℃, $CDCl_3$): δ 9.75 (s, 1H), 6.70 (s, 1H), 6.18 (s, 1H), 2.41 (s, 3H), 2.30 (s, 3H), 2,26 (s, 3H), 2.21 (s, 3H), 2.07 (s, 3H), 1.94 (s, 3H). ^{13}C NMR (100 MHz, 25 ℃, $CDCl_3$): δ 194.4, 141.8, 138.3, 137.8, 137.1, 137.0, 136.5, 131.4, 126.3, 125.7, 122.2, 119.1, 15.5, 15.4, 14.5, 14.1, 9.6, 9.0. ^{19}F NMR (376 MHz, 25 ℃, $CDCl_3$): δ -78.29. ESI-HRMS: m/z calcd for $C_{17}H_{21}OS_2Te^+$ $[M]^+$: 435.0091; found: 435.0088.

7: MeOTf (88 mg, 0.54 mmol) and *Z*-**5** (100 mg, 0.18 mmol) were dissolved and stirred in freshly distilled $CHCl_3$ (6 mL) under N_2 atmosphere. The mixture was stirred overnight in the dark at room temperature. The mixture was concentrated *in vacuo,* and then *n*-hexane (10 mL) was slowly added. The solid precipitates were collected by filtration and recrystallized from dichloromethane and *n*-hexane to give **7** (*Z*/*E* = 86:14) as a yellow-green solid (119 mg, 92%). *Z*-**7**: ^{1}H NMR (400 MHz, 25 ℃, DMSO-d_6): δ 9.92 (s, 1H), 7.58 (d, *J* = 7.2 Hz, 2H), 7.54 (s, 1H), 7.49-7.41 (m, 5H), 7.34 (t, *J* = 7.2 Hz, 2H), 7.27 (d, *J* = 7.2 Hz, 1H), 7.24 (s, 1H), 2.25 (s, 3H), 2.18 (s, 3H), 2.07 (s, 3H), 2.07 (s, 3H). ^{13}C NMR (100 MHz, 25 ℃, $CDCl_3$): δ 194.1, 142.6, 142.2, 141.7, 139.0, 138.0, 137.3, 133.6, 133.4, 132.7, 132.5, 129.3, 129.2, 128.3, 128.0, 125.9, 125.7, 124.3, 123.5, 14.7, 14.3, 9.6, 9.6. ^{19}F NMR (376 MHz, 25 ℃, DMSO-d_6): δ -77.78.

E-**7**: ^{1}H NMR (400 MHz, 25 ℃, DMSO-d_6): δ 9.49 (s, 1H), 7.71 (d, *J* = 7.2 Hz, 2H), 7.66 (d, *J* = 7.2 Hz, 2H), 7.52 (s, 1H), 7.41-7.30 (m, 7H), 2.54 (s, 3H), 2.44 (s, 3H), 2.27 (s, 3H), 2.06 (s, 3H). ^{13}C NMR (100 MHz, 25 ℃, $CDCl_3$): δ 187.4, 149.6, 146.0, 143.6, 143.5, 140.2, 133.0, 132.3, 130.3, 129.4, 128.7, 128.0, 126.0, 126.0, 125.4, 122.3, 119.1, 115.9, 14.0, 14.6, 14.4, 9.9. ESI-HRMS: m/z calcd for $C_{27}H_{25}OS_2Te^+$ $[M]^+$: 559.0404; found:559.0402.

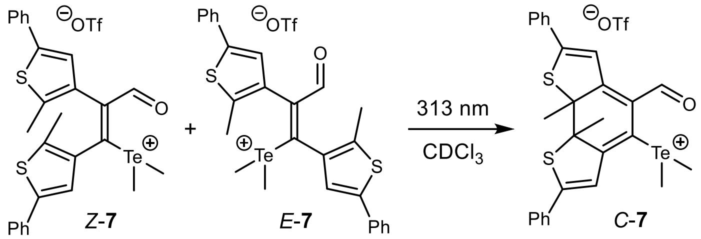

Supplementary Figure 4. Synthesis of *C*-**7**.

C-**7**: A solution of **7** (40 mg, 0.057 mmol) in $CHCl_3$ (5 mL) was irradiated with UV light (313 nm) for 4 h. The solid precipitates were collected by filtration and washed with *n*-hexane to give *C*-**7** as a dark solid (18 mg, 45 %). *C*-**7**: ^{1}H NMR (400 MHz, 25 ℃, DMSO-*d*$_6$): δ 10.08 (s, 1H), 8.00 (s, 1H), 7.81 (d, *J* = 7.2 Hz, 2H), 7.68-7.48 (m, 8H), 6.97 (s, 1H), 2.46 (s, 3H), 2.23 (s, 3H), 2.15 (s, 3H), 1.96 (s, 3H). ^{13}C NMR (100 MHz, 25 ℃, DMSO-*d*$_6$): δ 190.2, 169.4, 161.5, 156.3, 154.0, 132.2, 132.0, 131.9, 130.9, 129.3, 129.2, 127.5, 126.7, 125.3, 122.3, 119.1, 116.5, 114.9, 105.6, 66.8, 66.4, 25.0, 25.0, 10.4, 10.2. ^{19}F NMR (376 MHz, 25 ℃, DMSO-*d*$_6$): δ -77.78. ESI-HRMS: m/z calcd for $C_{27}H_{25}OS_2Te^+$ $[M]^+$: 559.0404; found:559.0401.

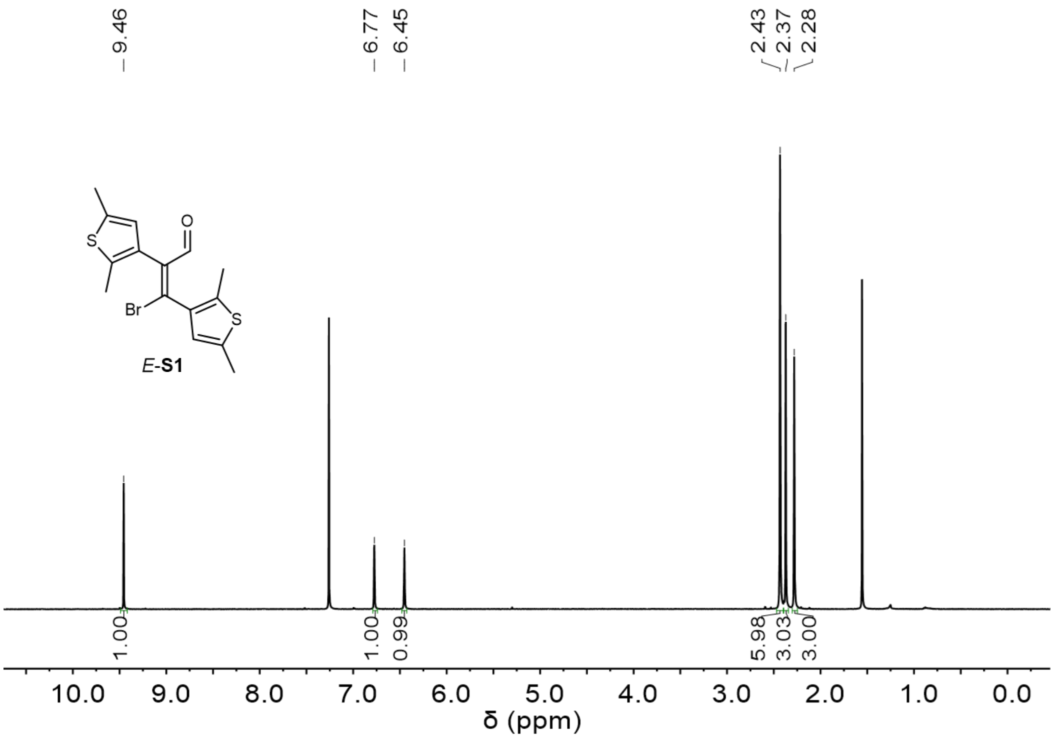

Supplementary Figure 5. ^{1}H NMR spectrum (400 MHz, 25 ℃) of *E*-**S1** in $CDCl_3$.

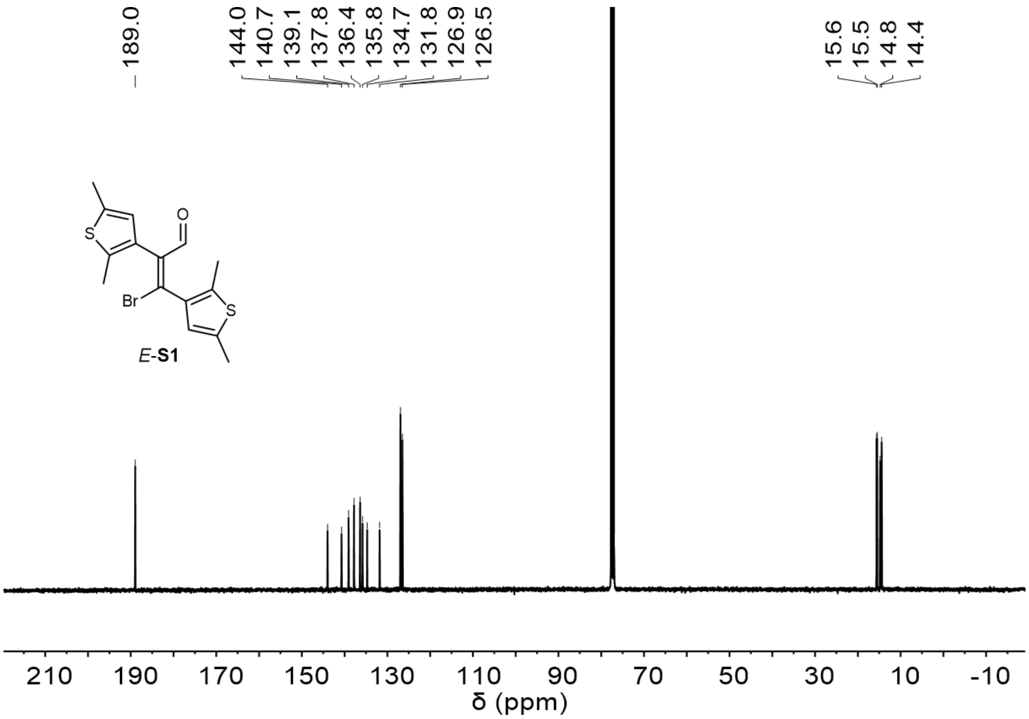

Supplementary Figure 6. ^{13}C NMR spectrum (100 MHz, 25 ℃) of *E*-**S1** in $CDCl_3$.

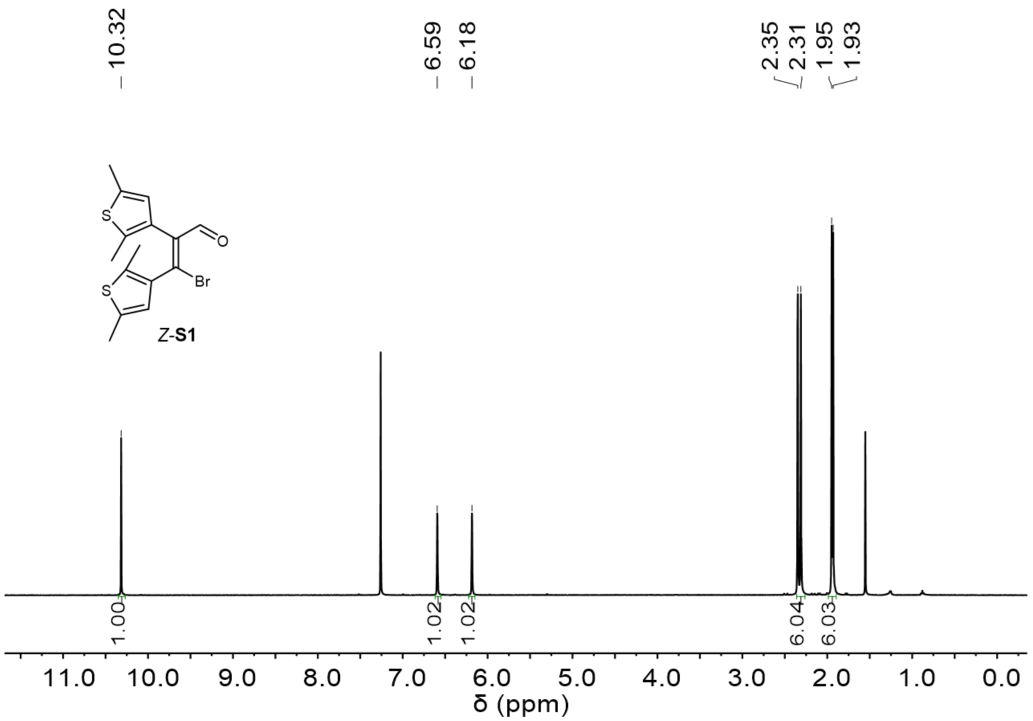

Supplementary Figure 7. ^{1}H NMR spectrum (400 MHz, 25 ℃) of *Z*-**S1** in $CDCl_3$.

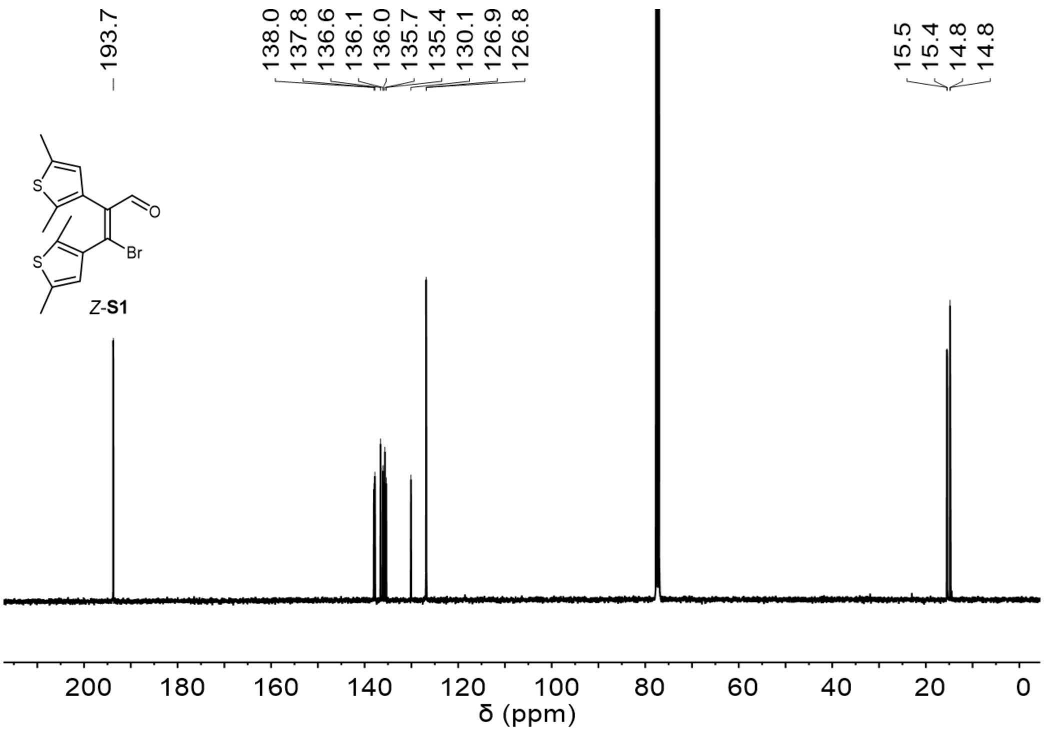

Supplementary Figure 8. ^{13}C NMR spectrum (100 MHz, 25 ℃) of *Z*-**S1** in $CDCl_3$.

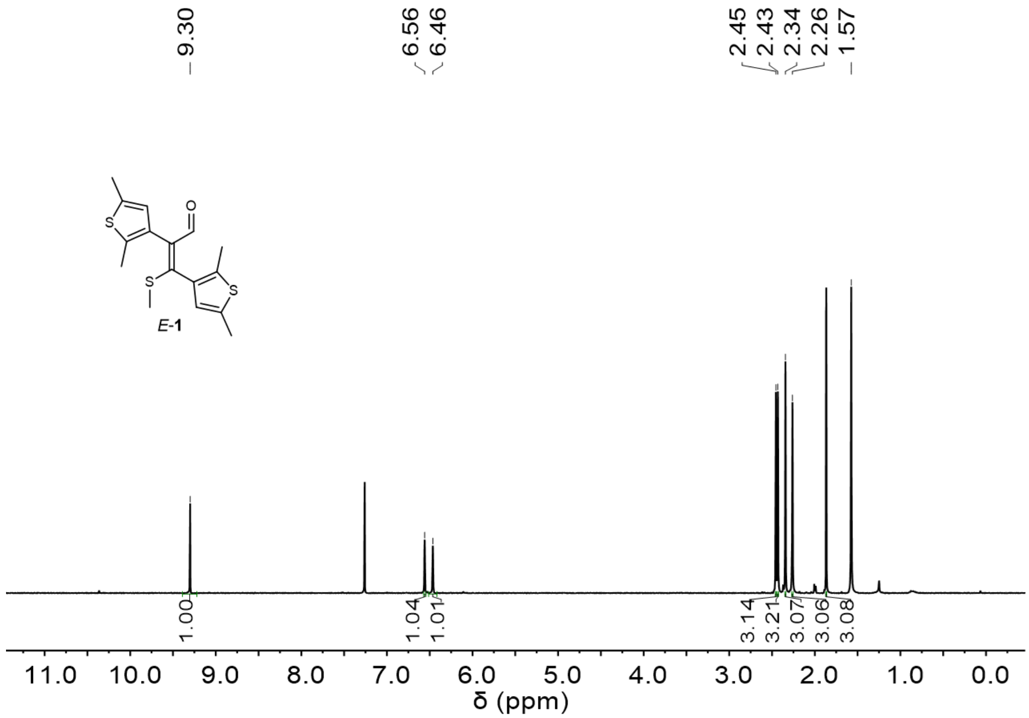

Supplementary Figure 9. ^{1}H NMR spectrum (400 MHz, 25 ℃) of *E*-**1** in $CDCl_3$.

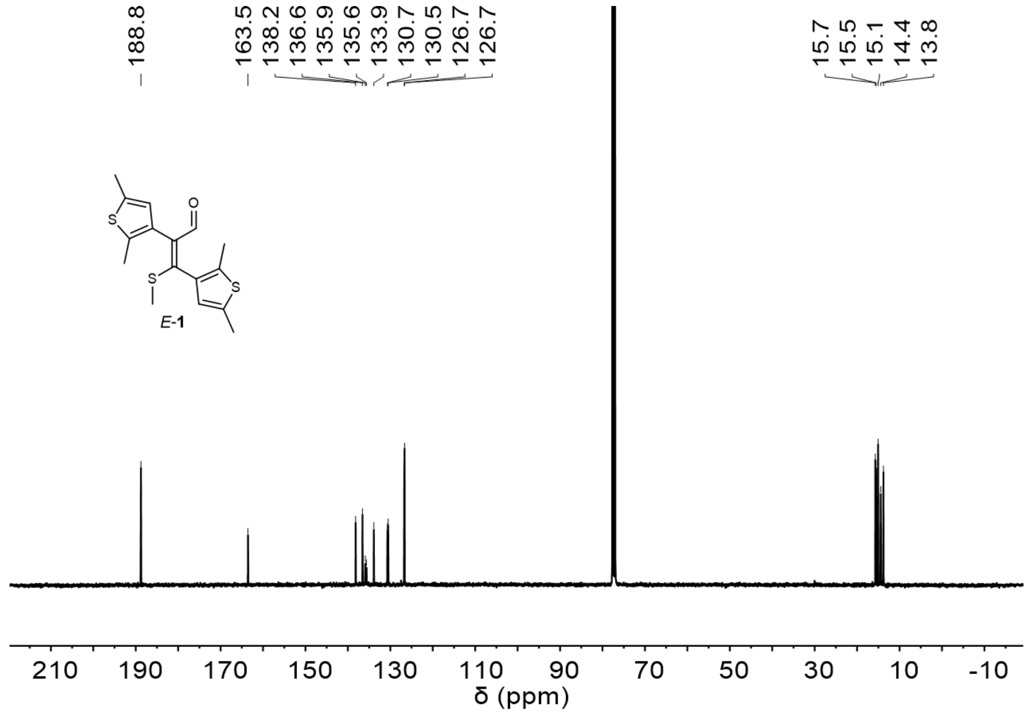

Supplementary Figure 10. ^{13}C NMR spectrum (100 MHz, 25 ℃) of *E*-**1** in $CDCl_3$.

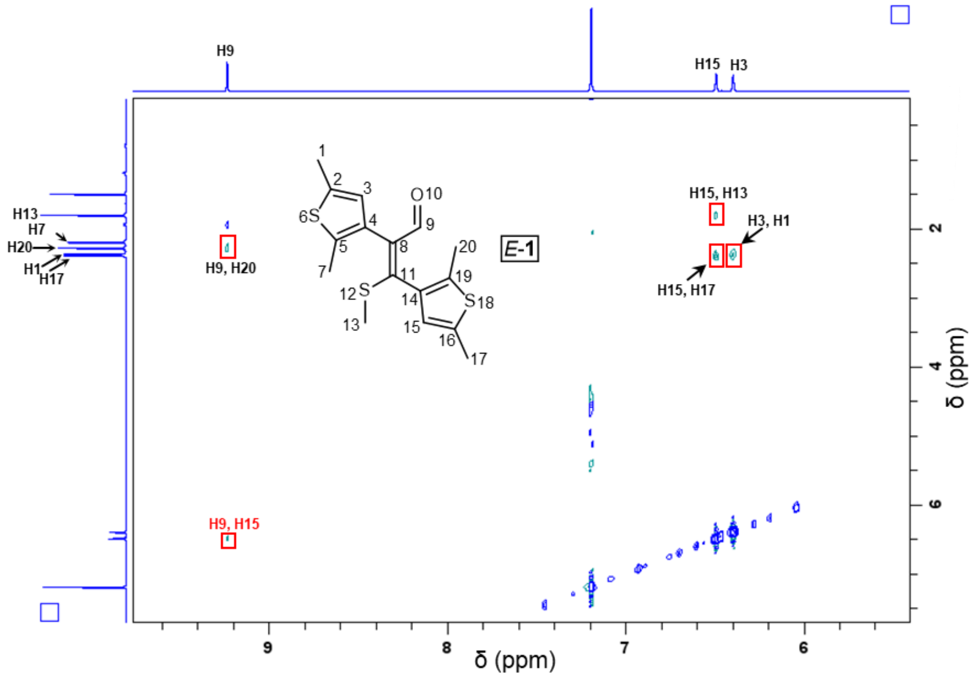

Supplementary Figure 11. Partial 2D ^1H-^1H NOESY NMR spectrum (400 MHz, 25 ℃) of *E*-**1** in $CDCl_3$. The cross signals between the protons at 9.30 ppm (H9 of *E*-**1**) and 6.56 ppm (H15 of *E*-**1**) confirm that the isomer is the *E* isomer.

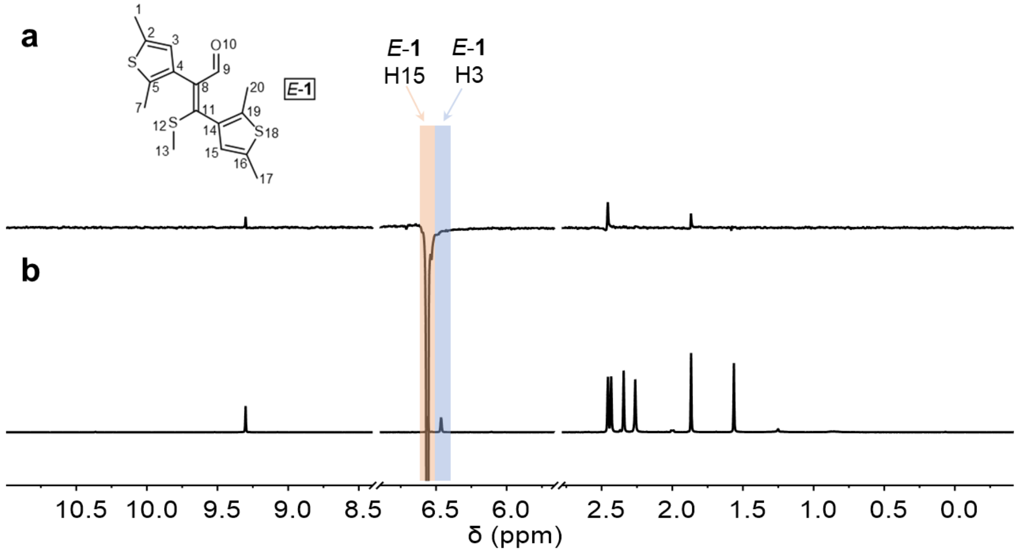

Supplementary Figure 12. 1D ^1H-^1H NOE NMR experiment of *E*-**1**. Irradiating the protons at 6.56 ppm (H15 of *E*-**1**) can't lead to signal increase at 6.46 (H3 of *E*-**1**). This confirms that the isomer is the *E* isomer. (a) 1D ^1H-^1H NOE NMR spectrum (400 MHz, $CDCl_3$, 25 ℃). (b) ^1H NMR spectrum (400 MHz, $CDCl_3$, 25 ℃).

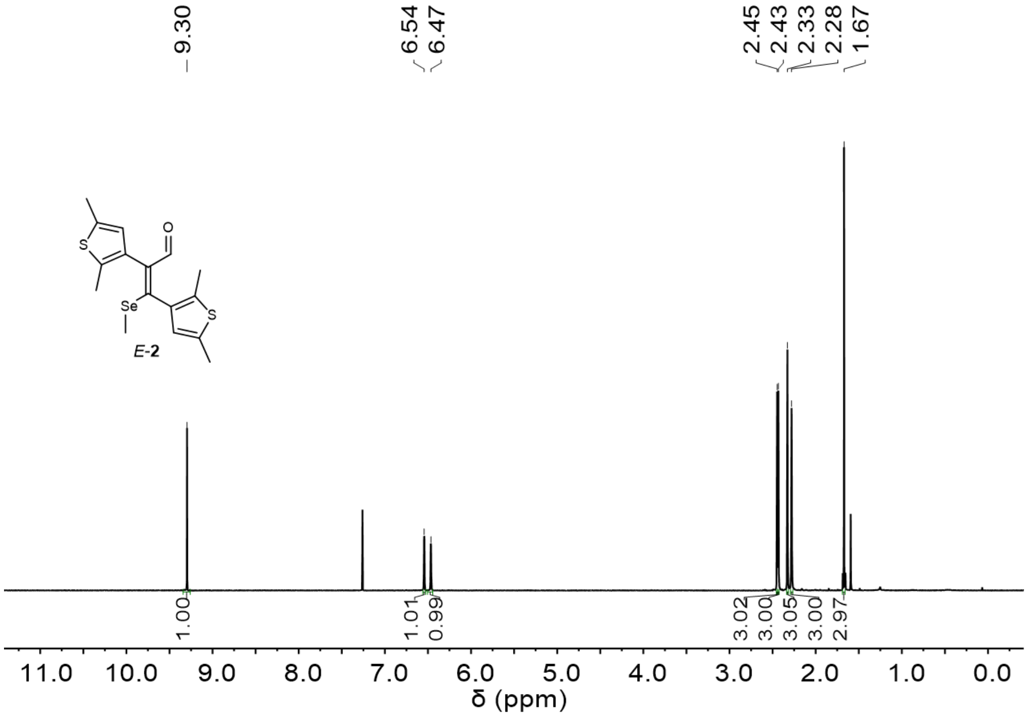

Supplementary Figure 13. ^{1}H NMR spectrum (400 MHz, 25 ℃) of *E*-**2** in $CDCl_3$.

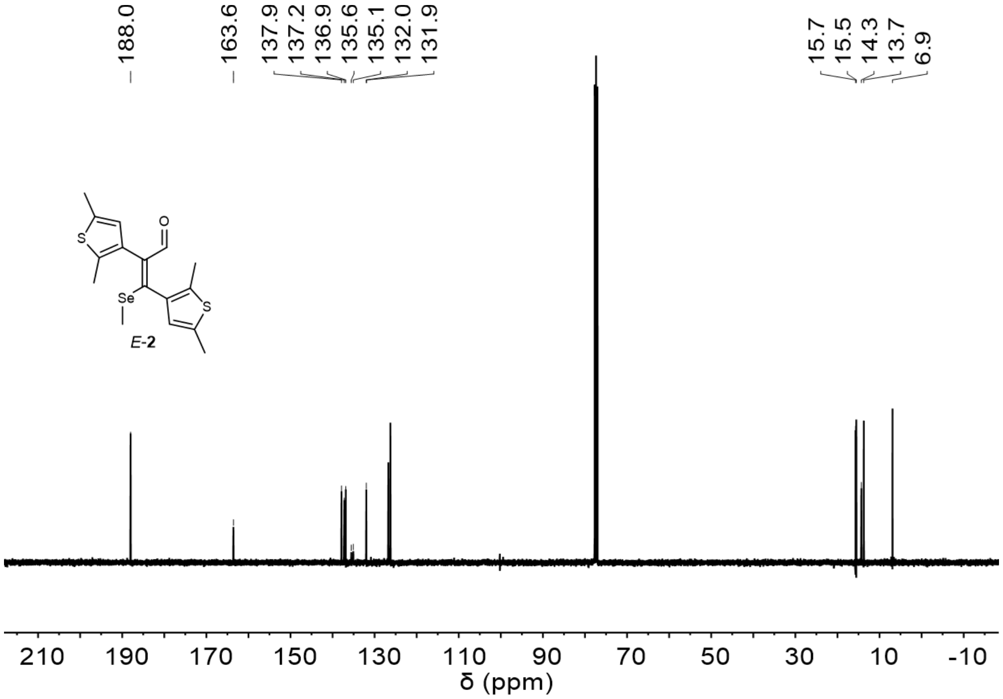

Supplementary Figure 14. ^{13}C NMR spectrum (100 MHz, 25 ℃) of *E*-**2** in $CDCl_3$.

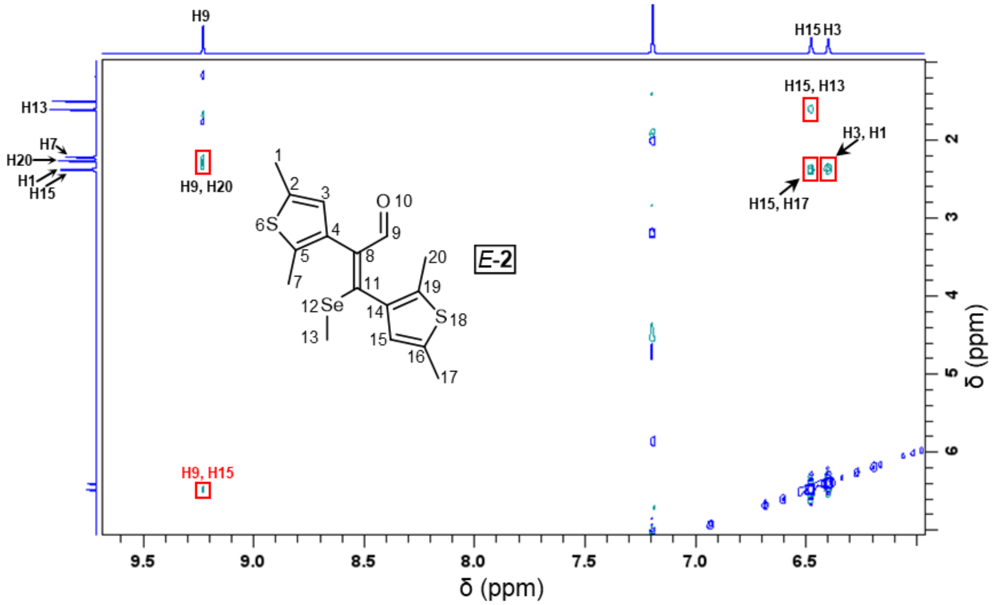

Supplementary Figure 15. Partial 2D ^{1}H-^{1}H NOESY NMR spectrum (400 MHz, 25 ℃) of *E*-**2** in $CDCl_3$. The cross signals between the protons at 9.30 ppm (H9 of *E*-**2**) and 6.54 ppm (H15 of *E*-**2**) confirm that the isomer is the *E* isomer.

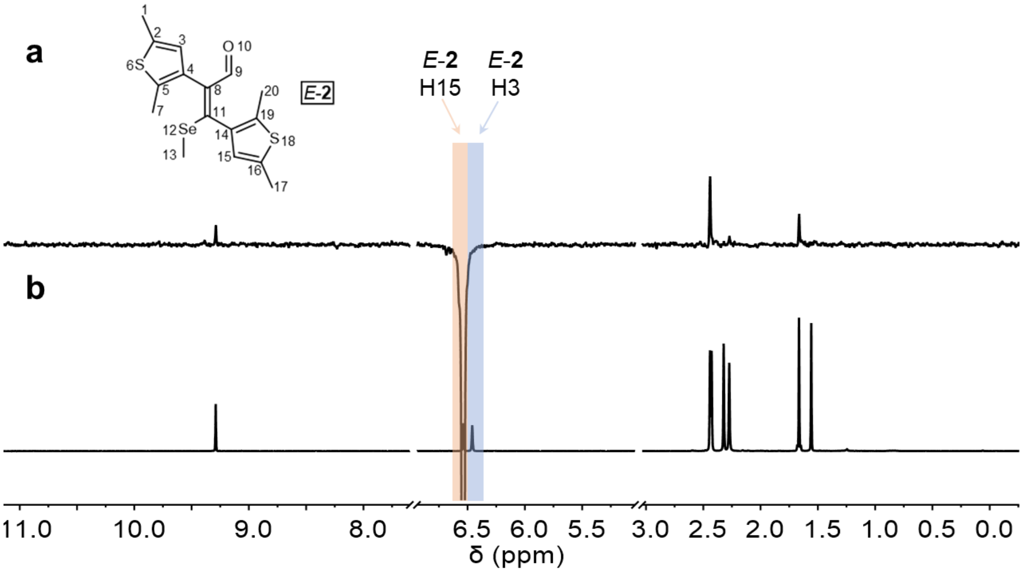

Supplementary Figure 16. 1D ^{1}H-^{1}H NOE NMR experiment of *E*-**2**. Irradiating the protons at 6.54 ppm (H15 of *E*-**2**) can't lead to signal increase at 6.47 ppm (H3 of *E*-**2**). This confirms that the isomer is the *E* isomer. (a) 1D ^{1}H-^{1}H NOE NMR spectrum (400 MHz, $CDCl_3$, 25 ℃). (b) ^{1}H NMR spectrum (400 MHz, $CDCl_3$, 25 ℃).

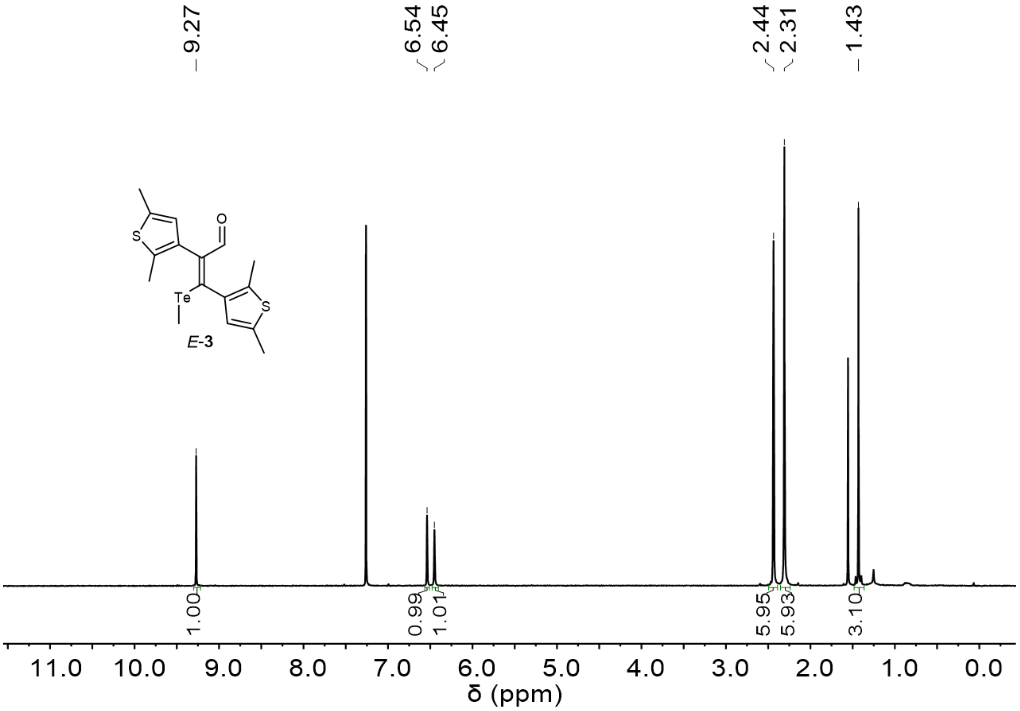

Supplementary Figure 17. ^{1}H NMR spectrum (400 MHz, 25 ℃) of *E*-**3** in $CDCl_3$.

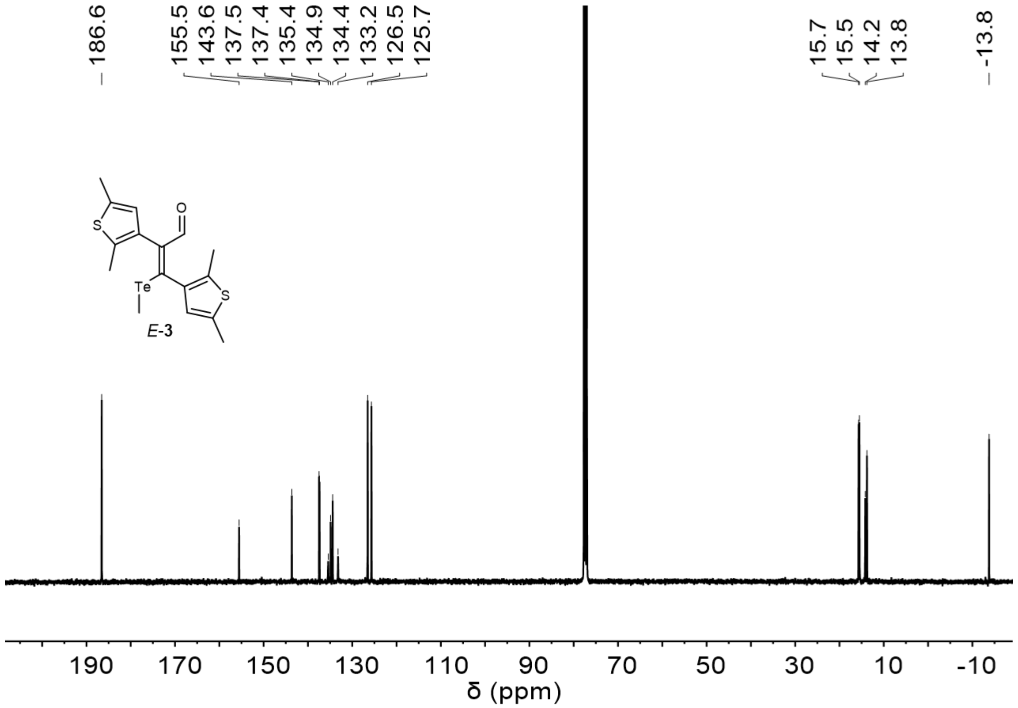

Supplementary Figure 18. ^{13}C NMR spectrum (100 MHz, 25 ℃) of *E*-**3** in $CDCl_3$.

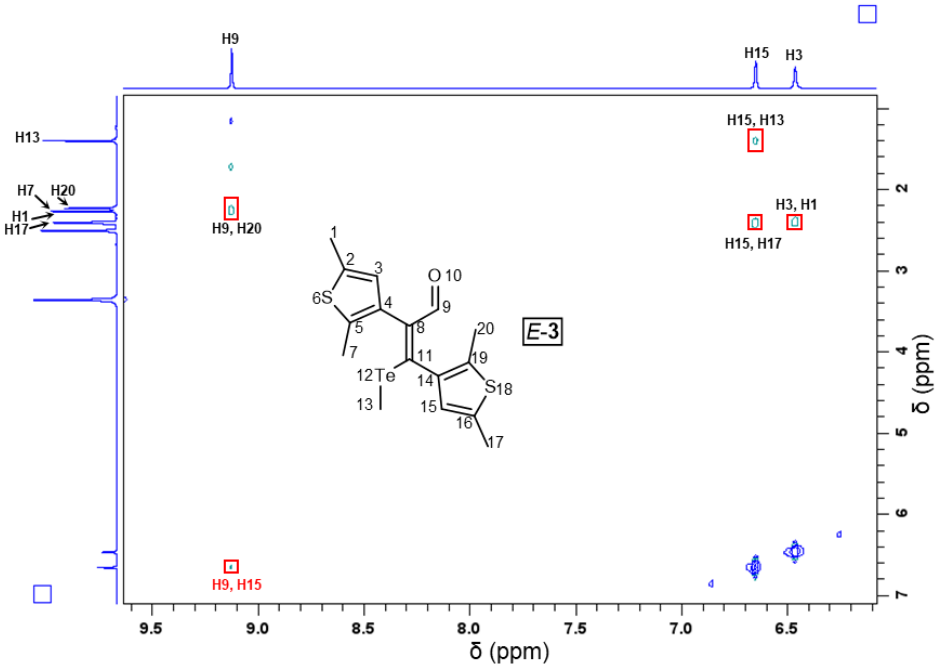

Supplementary Figure 19. Partial 2D ^{1}H-^{1}H NOESY NMR spectrum (400 MHz, 25 ℃) of *E*-**3** in DMSO-d_6. The cross signals between the protons at 9.13 ppm (H9 of *E*-**3**) and 6.65 ppm (H15 of *E*-**3**) confirm that the isomer is the *E* isomer.

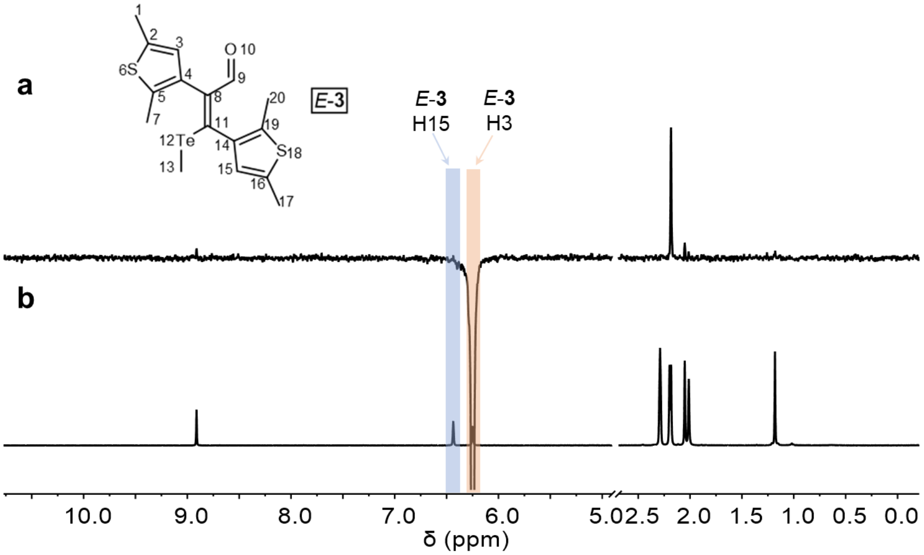

Supplementary Figure 20. 1D ^{1}H-^{1}H NOE NMR experiment of *E*-**3**. Irradiating the protons at 6.25 ppm (H3 of *E*-**3**) can't lead to signal increase at 6.44 ppm (H15 of *E*-**3**). This confirms that the isomer is the *E* isomer. (a) 1D ^{1}H-^{1}H NOE NMR spectrum (400 MHz, DMSO-d_6, 25 ℃). (b) ^{1}H NMR spectrum (400 MHz, DMSO-d_6, 25 ℃).

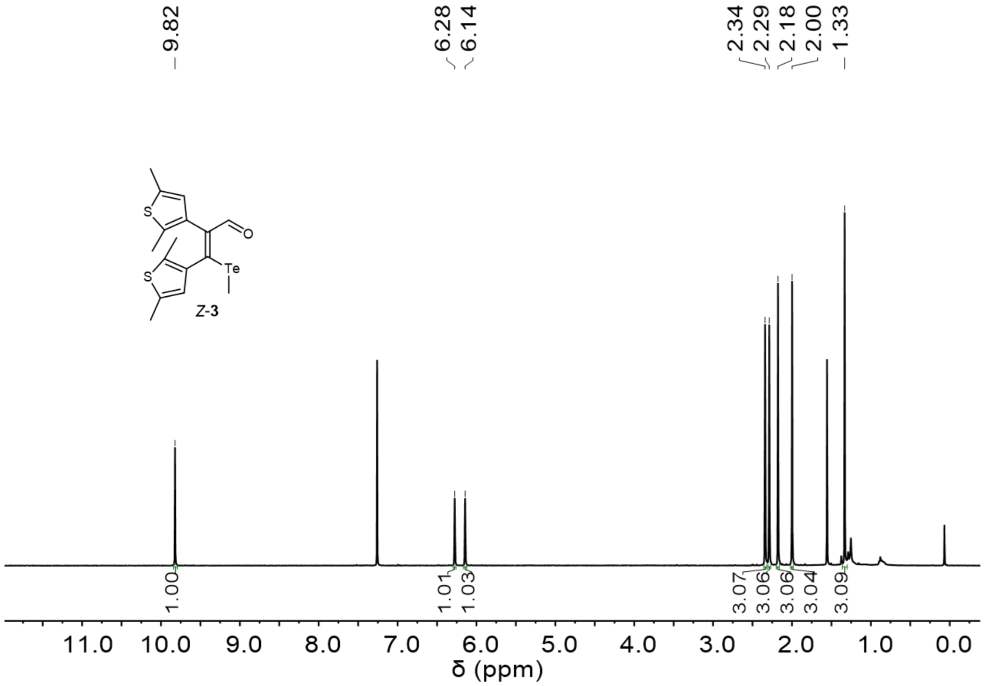

Supplementary Figure 21. ^{1}H NMR spectrum of (400 MHz, 25 ℃) *Z*-**3** in $CDCl_3$.

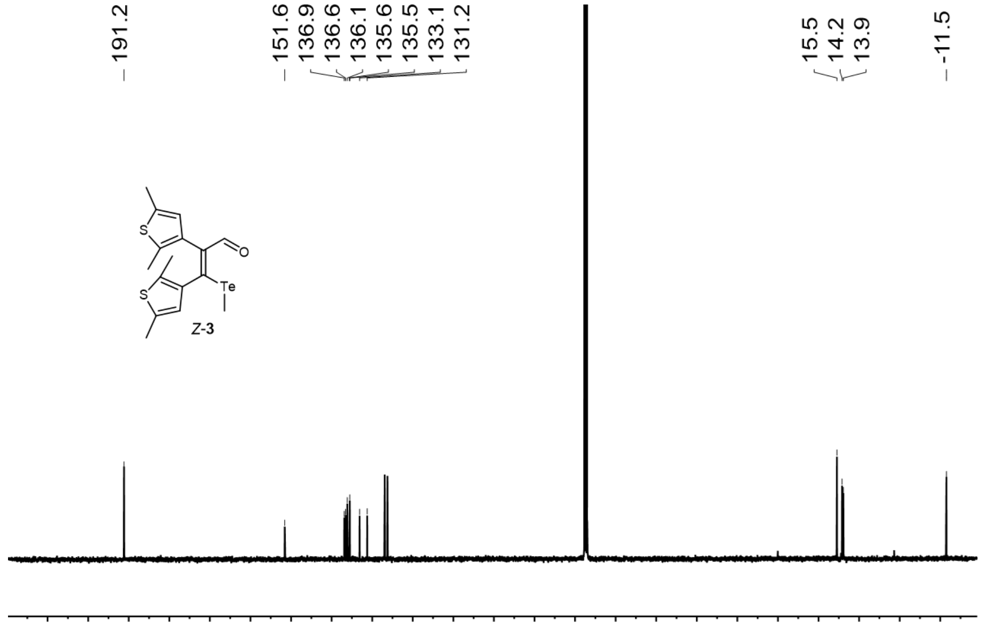

Supplementary Figure 22. ^{13}C NMR spectrum (100 MHz, 25 ℃) of *Z*-**3** in $CDCl_3$.

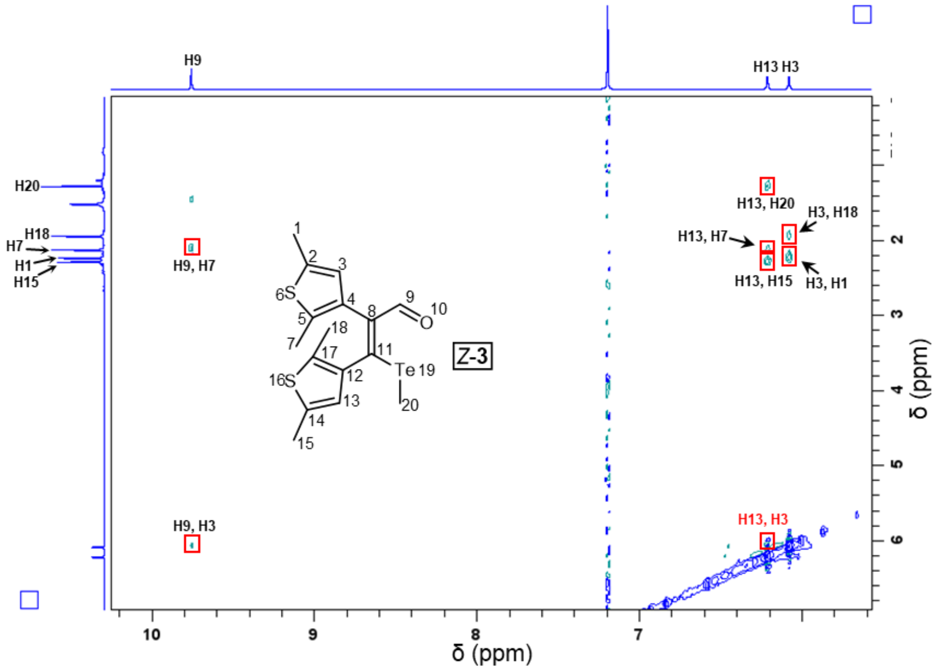

Supplementary Figure 23. Partial 2D ^{1}H-^{1}H NOESY NMR spectrum (400 MHz, 25 ℃) of *Z*-**3** in $CDCl_3$. The cross signals between the protons at 6.14 ppm (H3 of *Z*-**3**) and 6.28 ppm (H13 of *Z*-**3**) confirm that the isomer is the *Z* isomer.

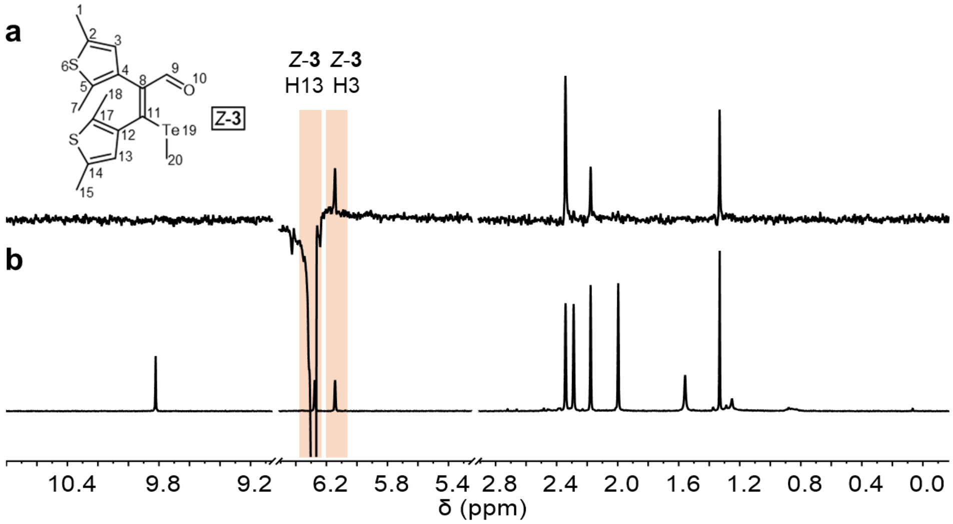

Supplementary Figure 24. 1D ^{1}H-^{1}H NOE NMR experiment of *Z*-**3**. Irradiating the protons at 6.28 ppm (H13 of *Z*-**3**) leads to signal increase at 6.14 ppm (H3 of *Z*-**3**). This confirms that the isomer is the *Z* isomer. (a) 1D ^{1}H-^{1}H NOE NMR spectrum (400 MHz, $CDCl_3$, 25 ℃). (b) ^{1}H NMR spectrum (400 MHz, $CDCl_3$, 25 ℃).

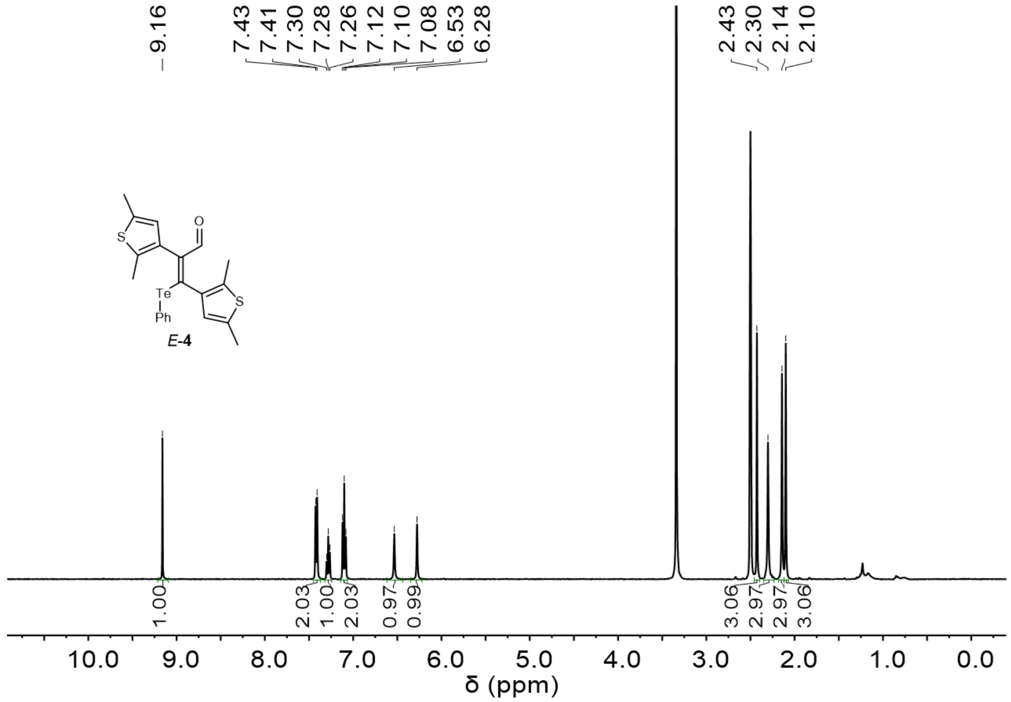

Supplementary Figure 25. ^{1}H NMR spectrum (400 MHz, 25 ℃) of *E*-**4** in DMSO-d_6.

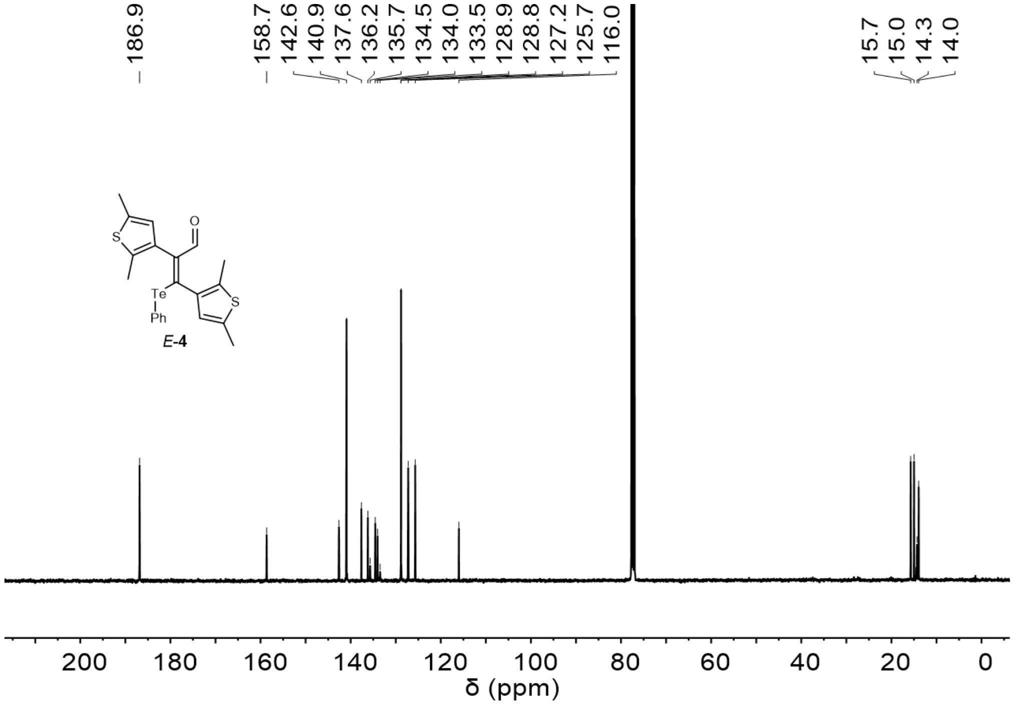

Supplementary Figure 26. ^{13}C NMR spectrum (100 MHz, 25 ℃) of *E*-**4** in $CDCl_3$.

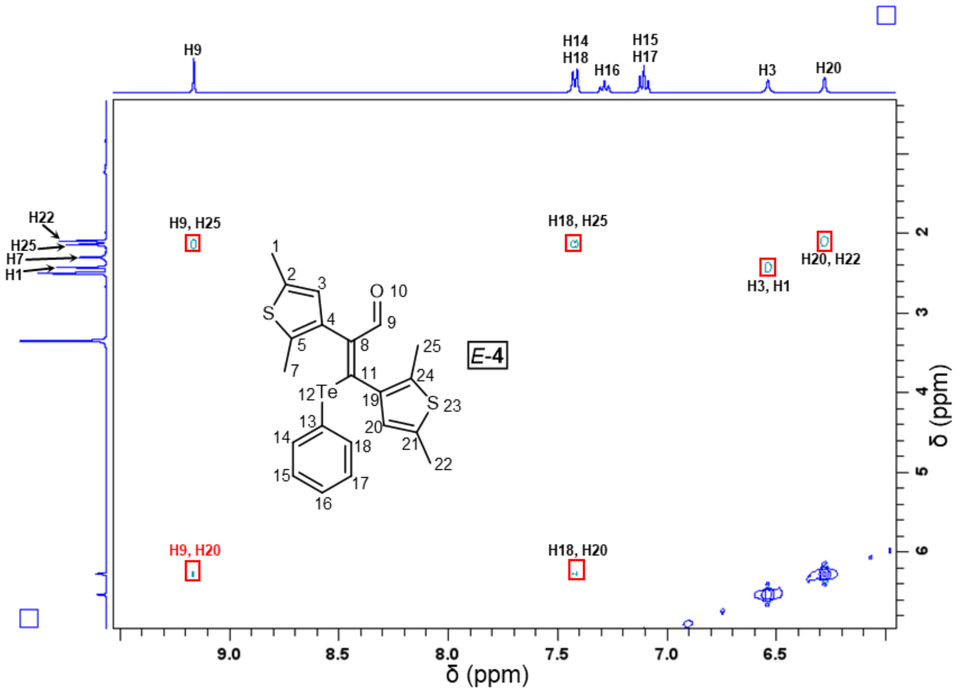

Supplementary Figure 27. Partial 2D ^{1}H-^{1}H NOESY NMR spectrum (400 MHz, 25 ℃) of *E*-**4** in DMSO-d_6. The cross signals between the protons at 9.16 ppm (H9 of *E*-**4**) and 6.28 ppm (H20 of *E*-**4**) confirm that the isomer is the *E* isomer.

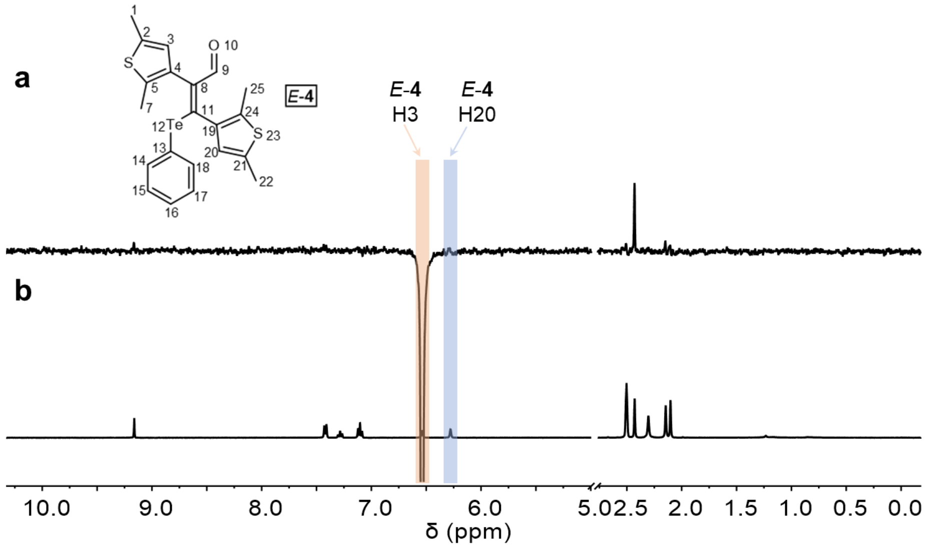

Supplementary Figure 28. 1D ^{1}H-^{1}H NOE NMR experiment of *E*-**4**. Irradiating the protons at 6.54 ppm (H3 of *E*-**4**) can't lead to signal increase at 6.28 ppm (H20 of *E*-**4**). This confirms that the isomer is the *E* isomer. (a) 1D ^{1}H-^{1}H NOE NMR spectrum (400 MHz, DMSO-d_6, 25 ℃). (b) ^{1}H NMR spectrum (400 MHz, DMSO-d_6, 25 ℃).

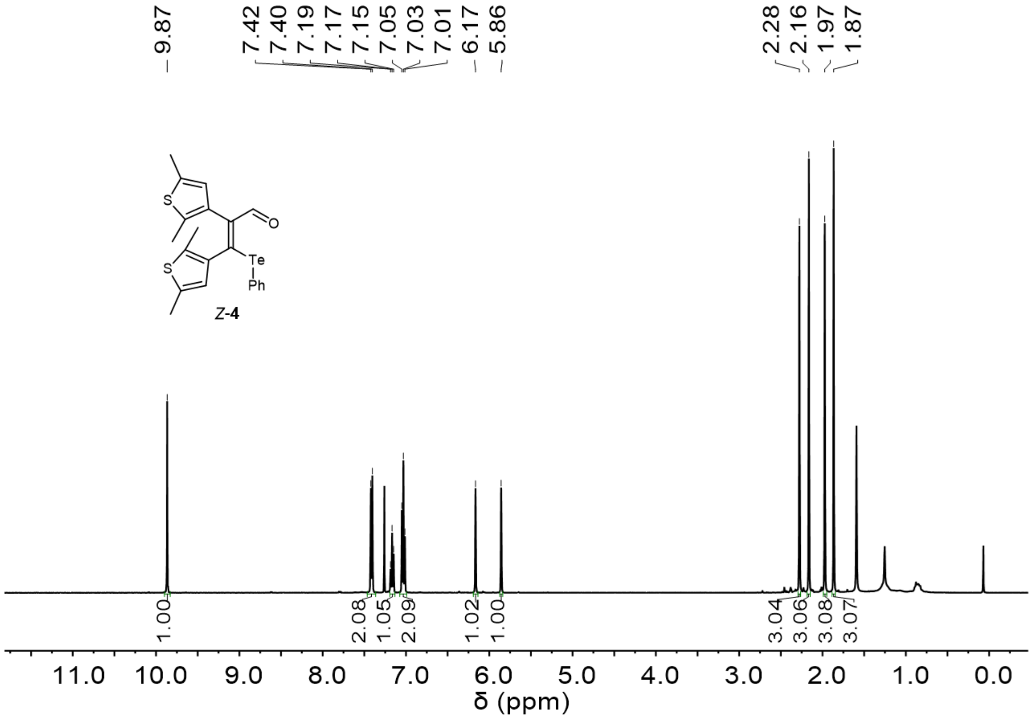

Supplementary Figure 29. ^{1}H NMR spectrum (400 MHz, 25 ℃) of *Z*-**4** in $CDCl_3$.

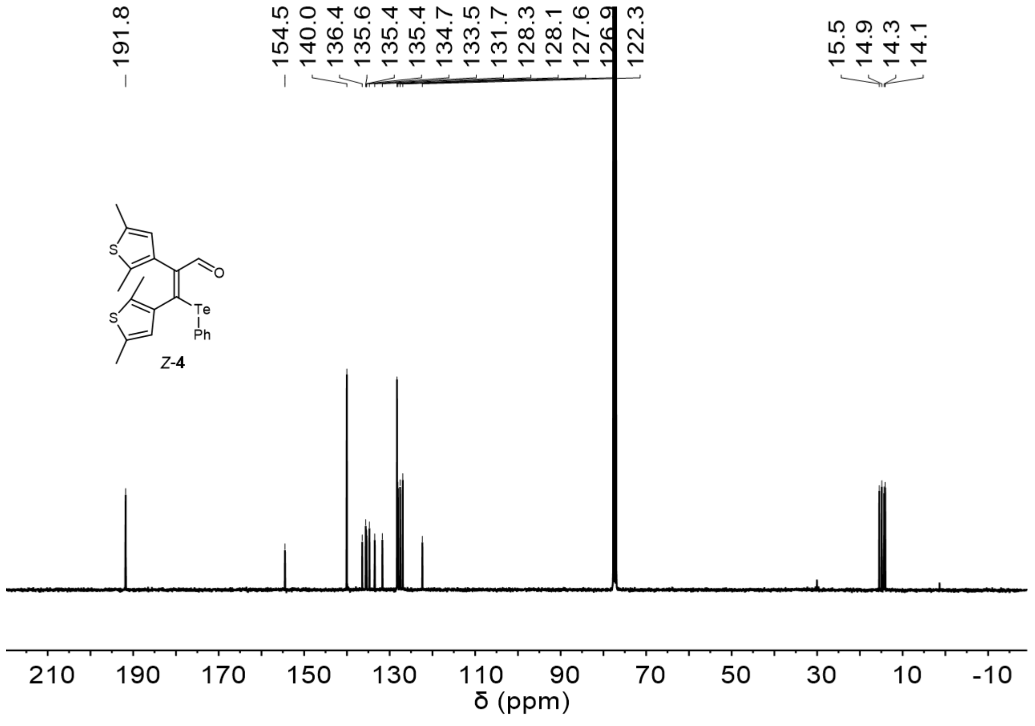

Supplementary Figure 30. ^{13}C NMR spectrum (100 MHz, 25 ℃) of *Z*-**4** in $CDCl_3$.

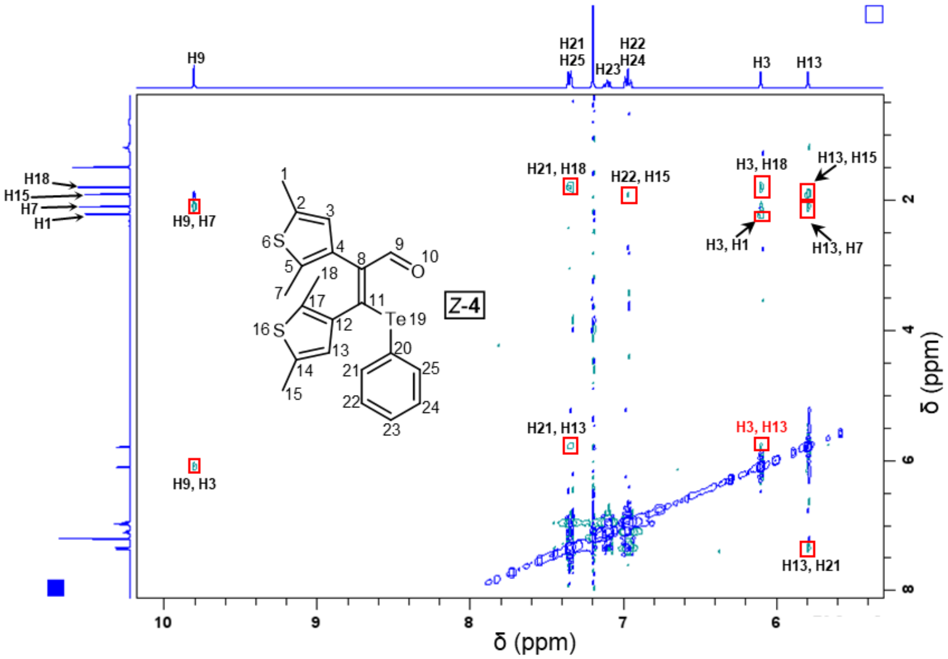

Supplementary Figure 31. Partial 2D ^{1}H-^{1}H NOESY NMR spectrum (400 MHz, 25 ℃) of *Z*-**4** in $CDCl_3$. The cross signals between the protons at 6.16 ppm (H3 of *Z*-**4**) and 5.86 ppm (H13 of *Z*-**4**) confirm that the isomer is the *Z* isomer.

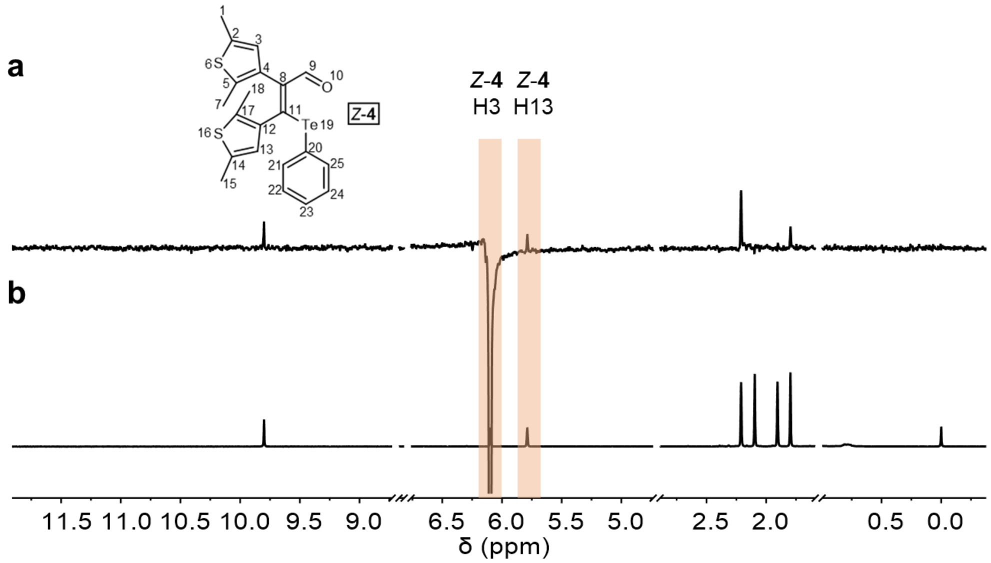

Supplementary Figure 32. 1D ^{1}H-^{1}H NOE NMR experiment of *Z*-**4**. Irradiating the protons at 6.16 ppm (H3 of *Z*-**3**) leads to signal increase at 5.86 ppm (H13 of *Z*-**4**). This confirms that the isomer is the *Z* isomer. (a) 1D ^{1}H-^{1}H NOE NMR spectrum (400 MHz, $CDCl_3$, 25 ℃). (b) ^{1}H NMR spectrum (400 MHz, $CDCl_3$, 25 ℃).

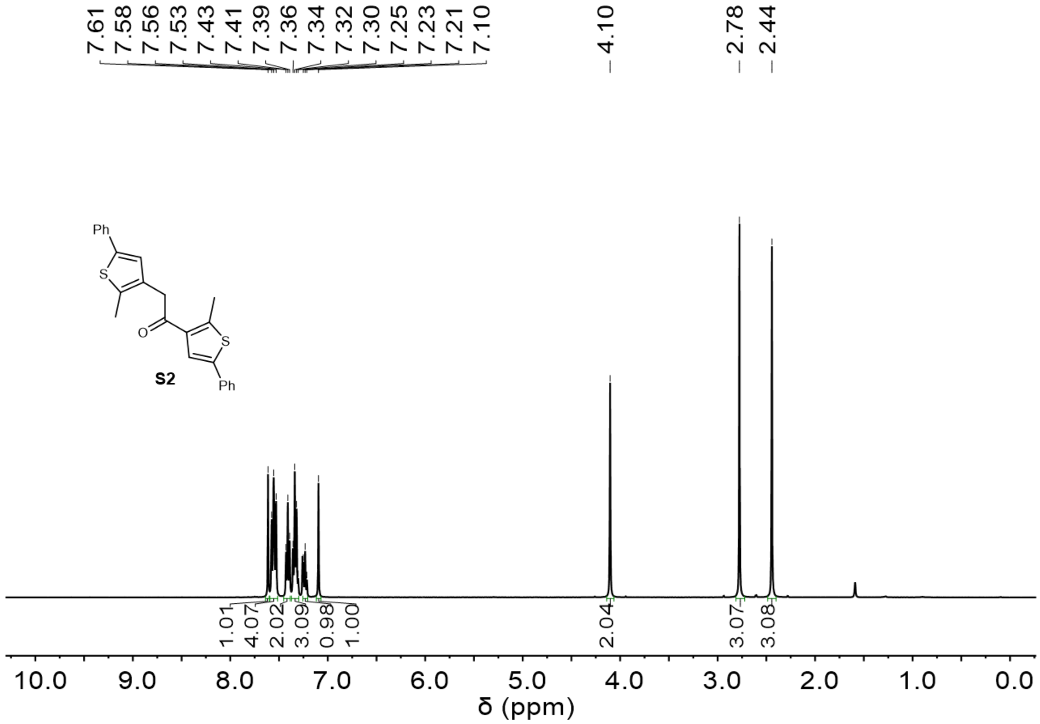

Supplementary Figure 33. ^{1}H NMR spectrum (400 MHz, 25 ℃) of **S2** in $CDCl_3$.

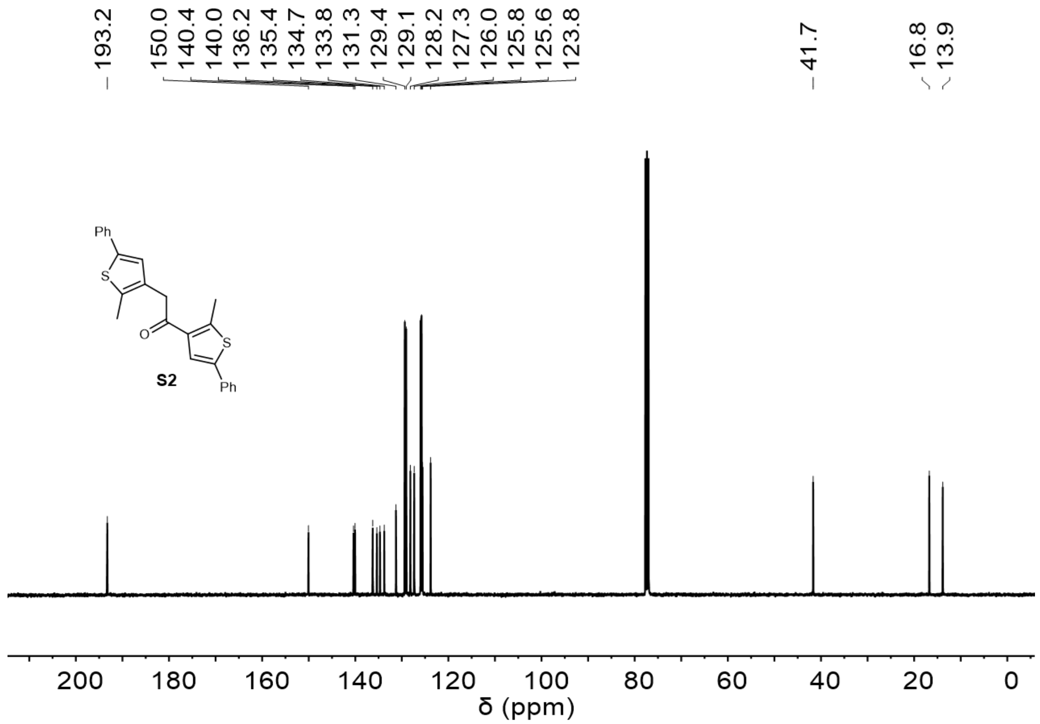

Supplementary Figure 34. ^{13}C NMR spectrum (100 MHz, 25 ℃) of **S2** in $CDCl_3$.

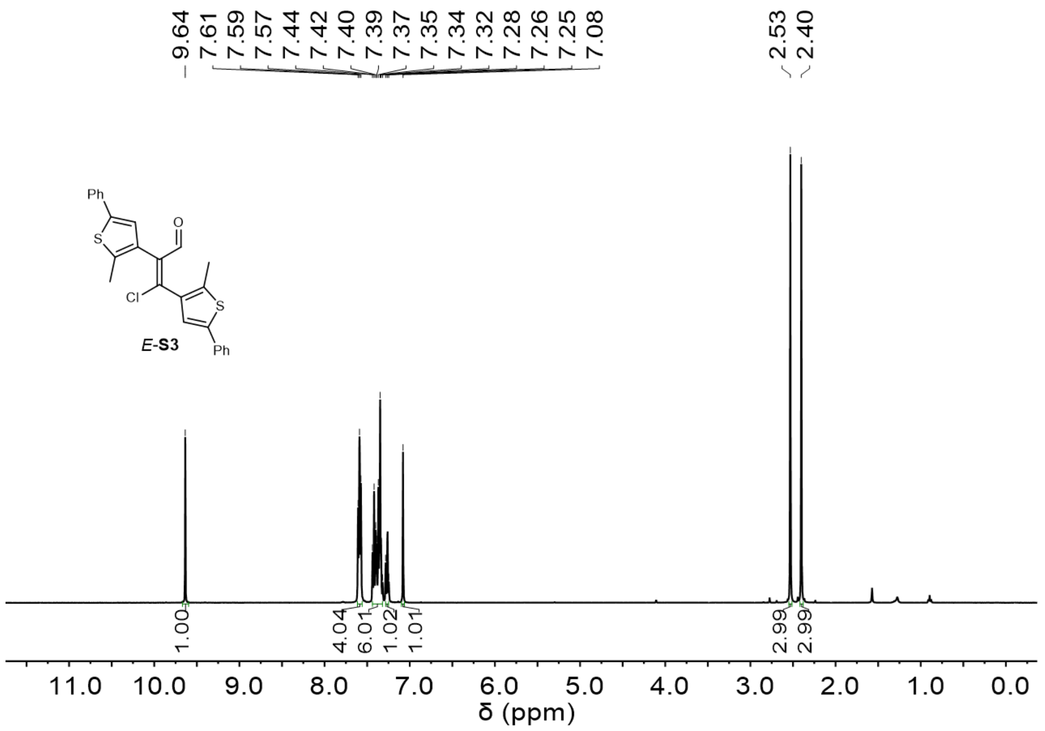

Supplementary Figure 35. ^{1}H NMR spectrum (400 MHz, 25 ℃) of *E*-**S3** in $CDCl_3$.

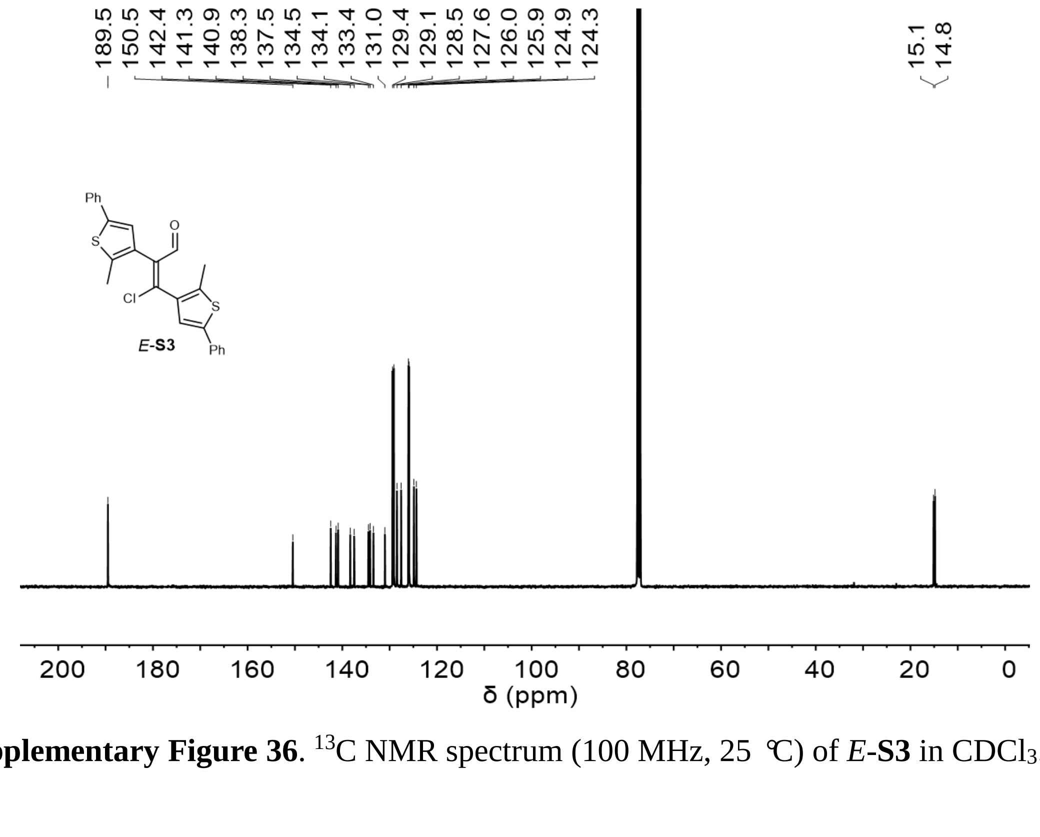

Supplementary Figure 36. ^{13}C NMR spectrum (100 MHz, 25 ℃) of *E*-**S3** in $CDCl_3$.

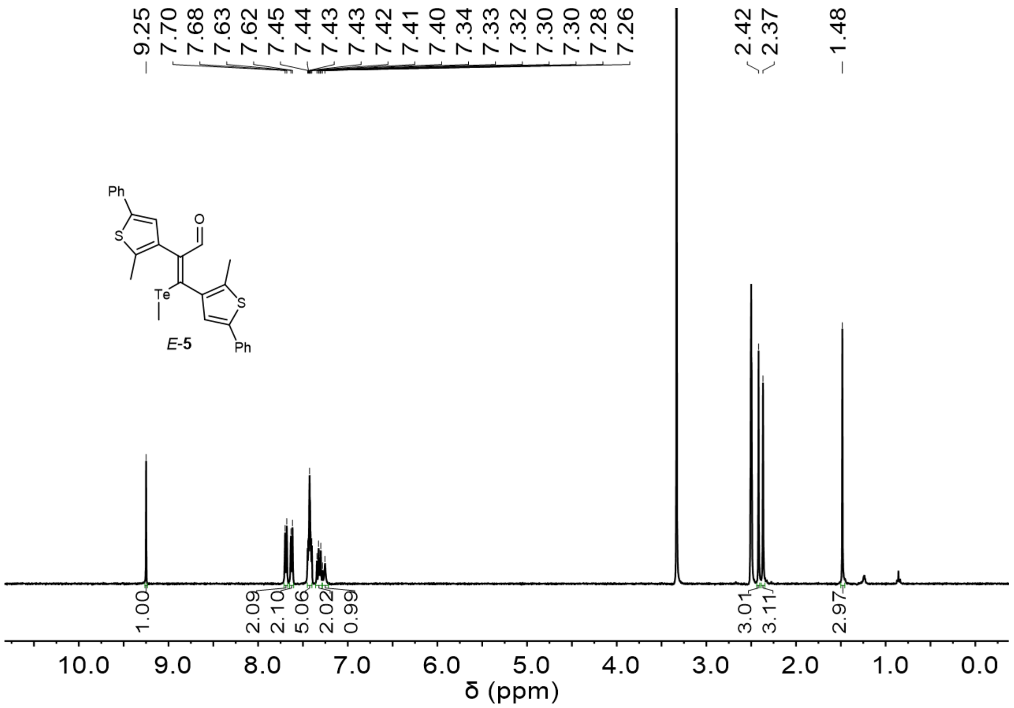

Supplementary Figure 37. ^{1}H NMR spectrum (400 MHz, 25 ℃) of *E*-**5** in DMSO-d_6.

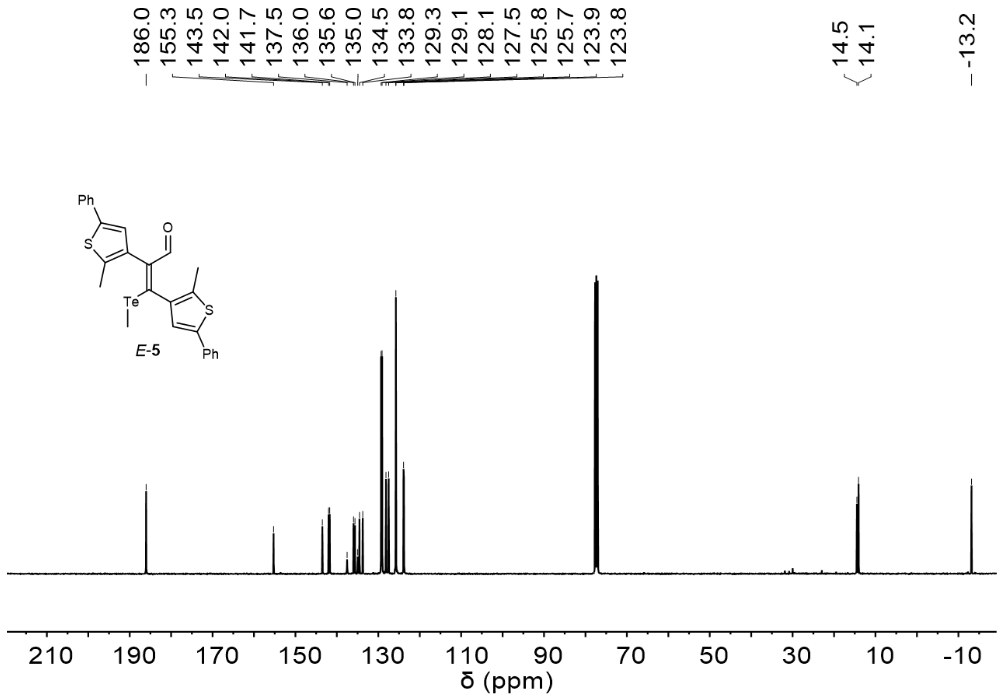

Supplementary Figure 38. ^{13}C NMR spectrum (100 MHz, 25 ℃) of *E*-**5** in $CDCl_3$.

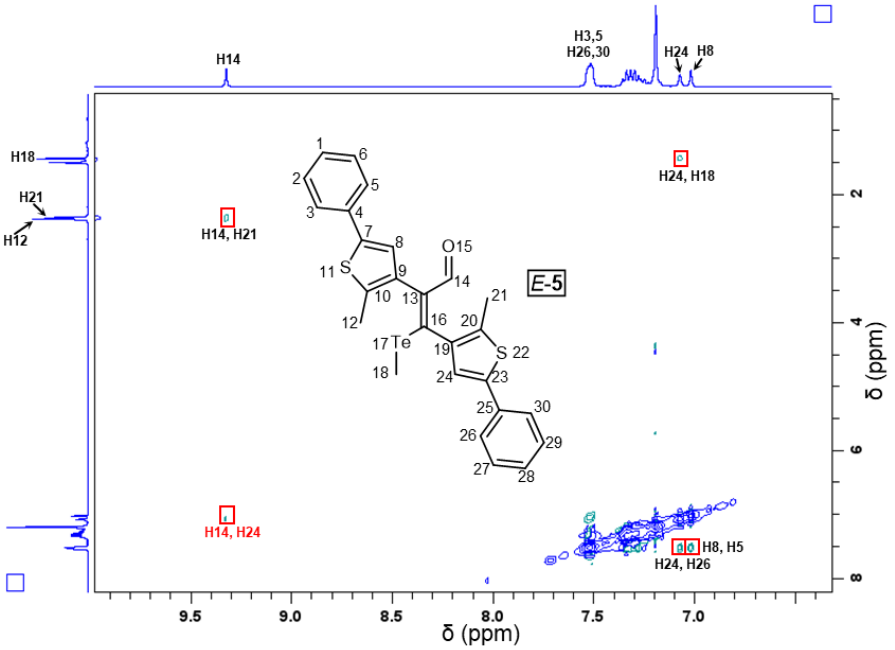

Supplementary Figure 39. Partial 2D ^1H-^1H NOESY NMR spectrum (400 MHz, 25 ℃) of *E*-**5** in $CDCl_3$. The cross signals between the protons at 9.39 ppm (H14 of *E*-**5**) and 7.14 ppm (H24 of *E*-**5**) confirm that the isomer is the *E* isomer.

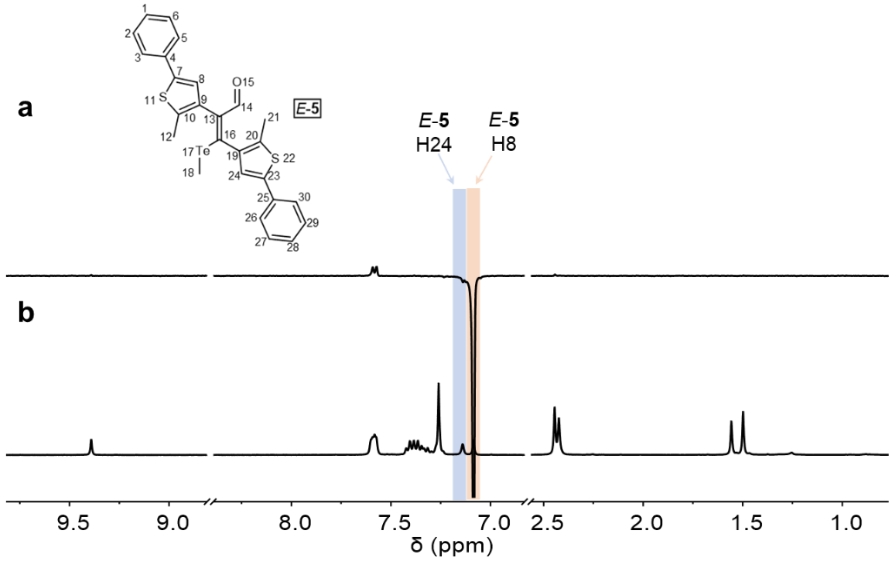

Supplementary Figure 40. 1D ^1H-^1H NOE NMR experiment of *E*-**5**. Irradiating the protons at 7.08 ppm (H8 of *E*-**5**) can't lead to signal increase at 7.14 ppm (H24 of *E*-**5**). This confirms that the isomer is the *E* isomer. (a) 1D ^1H-^1H NOE NMR spectrum (400 MHz, $CDCl_3$, 25 ℃). (b) ^1H NMR spectrum (400 MHz, $CDCl_3$, 25 ℃).

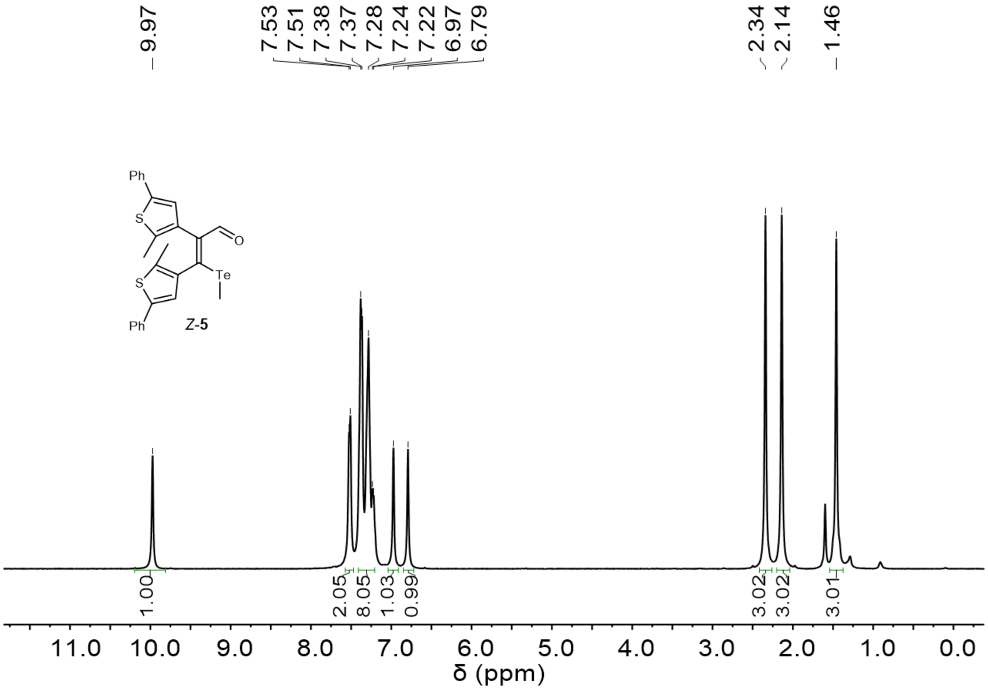

Supplementary Figure 41. ^{1}H NMR spectrum (400 MHz, 25 ℃) of *Z*-**5** in $CDCl_3$.

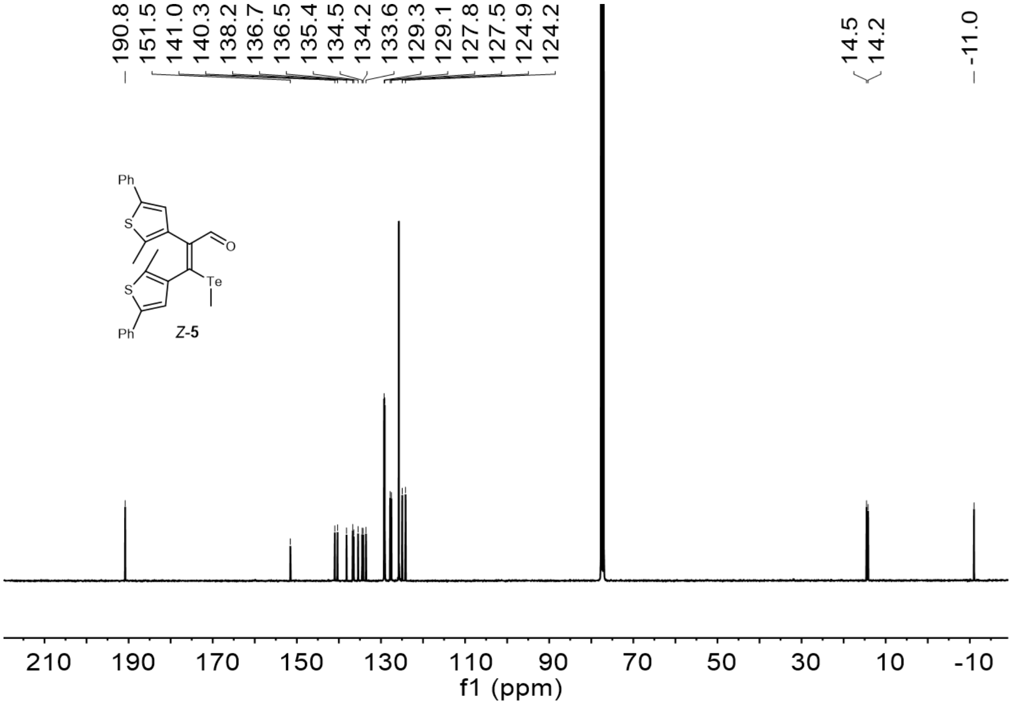

Supplementary Figure 42. ^{13}C NMR spectrum (100 MHz, 25 ℃) of *Z*-**5** in $CDCl_3$.

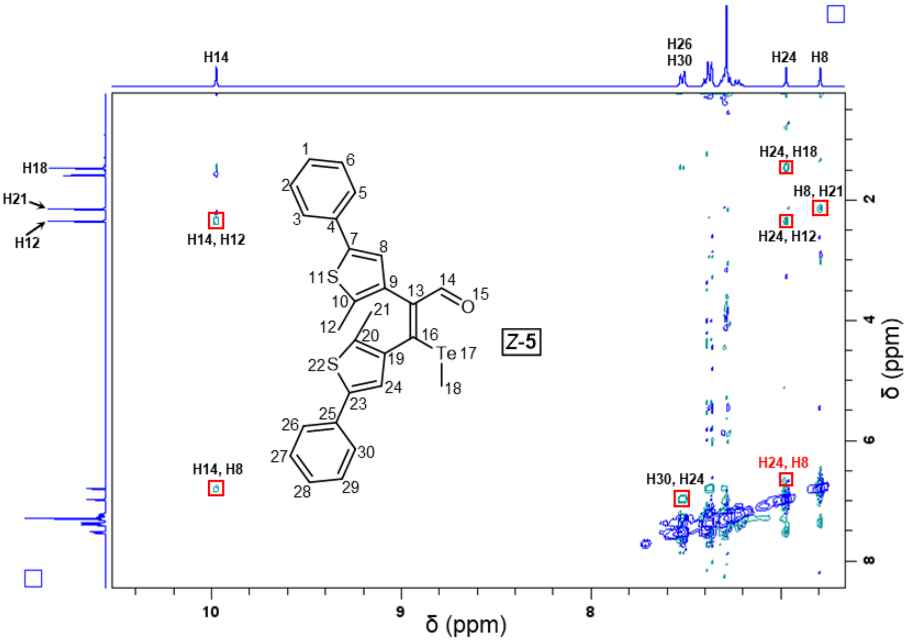

Supplementary Figure 43. Partial 2D ^1H-^1H NOESY NMR spectrum (400 MHz, 25 ℃) of *Z*-**5** in $CDCl_3$. The cross signals between the protons at 6.95 ppm (H24 of *Z*-**5**) and 6.77 ppm (H8 of *Z*-**5**) confirm that the isomer is the *Z* isomer.

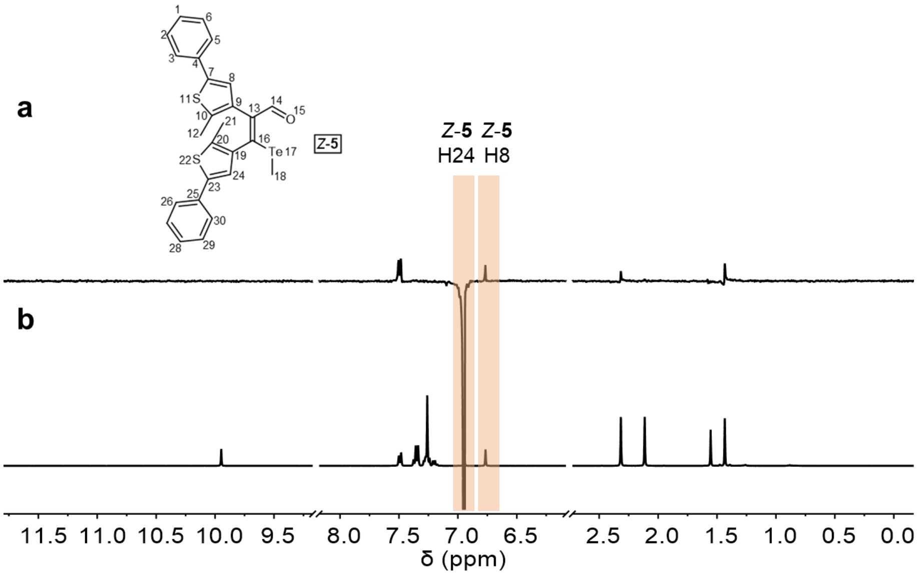

Supplementary Figure 44. 1D ^1H-^1H NOE NMR experiment of *Z*-**5**. Irradiating the protons at 6.95 ppm (H24 of *Z*-**5**) leads to signal increase at 6.77 ppm (H8 of *Z*-**5**). This confirms that the isomer is the *Z* isomer. (a) 1D ^1H-^1H NOE NMR spectrum (400 MHz, $CDCl_3$, 25 ℃). (b) ^1H NMR spectrum (400 MHz, $CDCl_3$, 25 ℃).

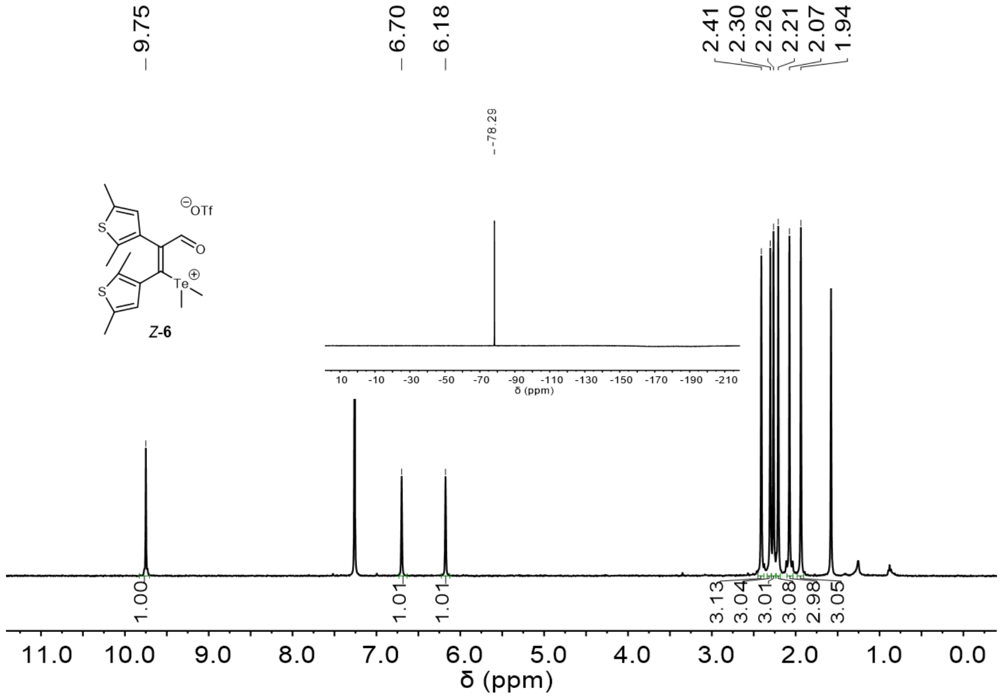

Supplementary Figure 45. ^{1}H NMR spectrum (400 MHz, 25 ℃) of *Z*-**6** in $CDCl_3$. Inset: ^{19}F NMR spectrum (376 MHz, 25 ℃) of *Z*-**6** in $CDCl_3$.

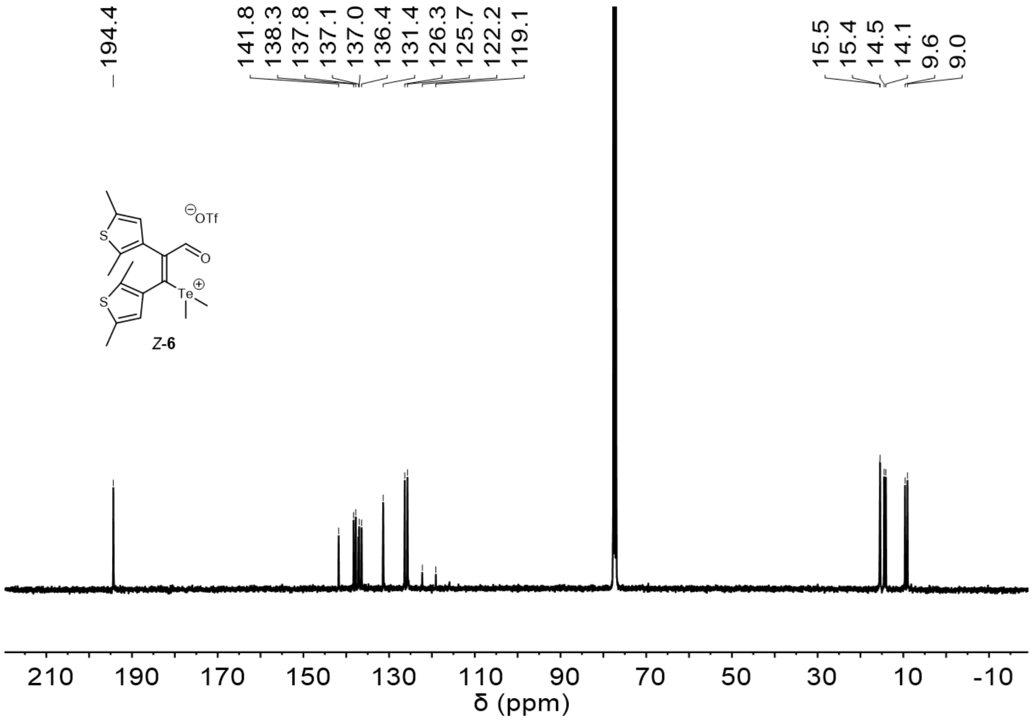

Supplementary Figure 46. ^{13}C NMR (100 MHz, 25 ℃) spectrum of *Z*-**6** in $CDCl_3$.

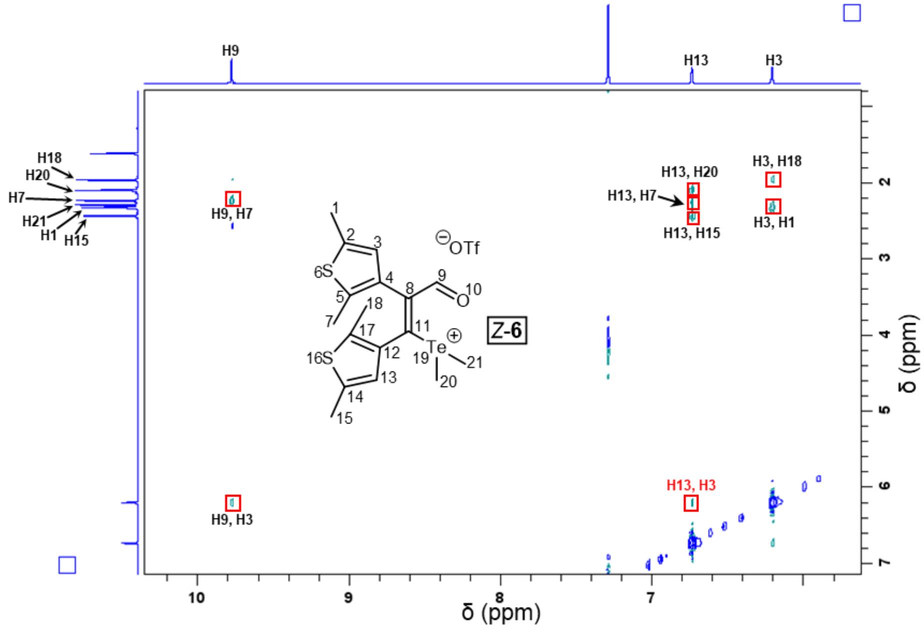

Supplementary Figure 47. Partial 2D ^1H-^1H NOESY NMR spectrum (400 MHz, 25 ℃) of *Z*-**6** in $CDCl_3$. The cross signals between the protons at 6.71 ppm (H13 of *Z*-**6**) and 6.18 ppm (H3 of *Z*-**6**) confirm that the isomer is the *Z* isomer.

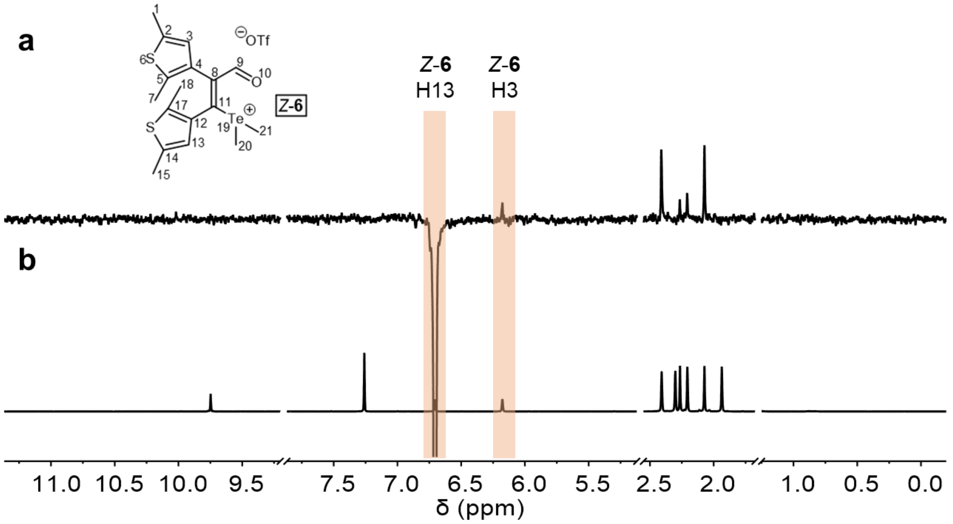

Supplementary Figure 48. 1D ^1H-^1H NOE NMR experiment of *Z*-**6**. Irradiating the protons at 6.71 ppm (H13 of *Z*-**6**) leads to signal increase at 6.18 ppm (H3 of *Z*-**6**). This confirms that the isomer is the *Z* isomer. (a) 1D ^1H-^1H NOE NMR spectrum (400 MHz, $CDCl_3$, 25 ℃). (b) ^1H NMR spectrum (400 MHz, $CDCl_3$, 25 ℃).

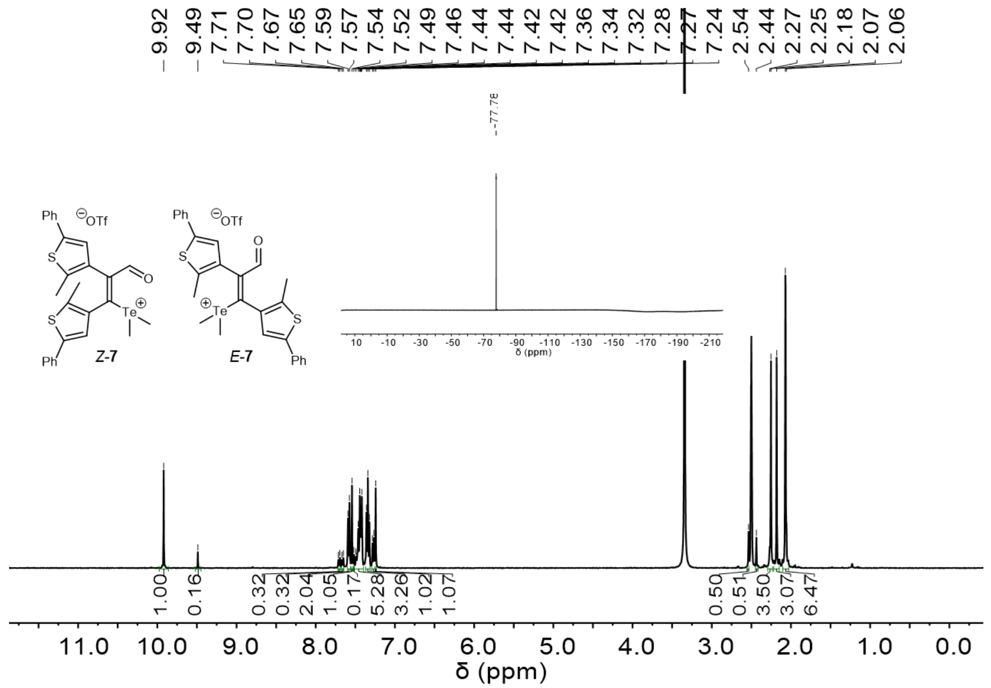

Supplementary Figure 49. ^{1}H NMR spectrum (400 MHz, 25 ℃) of **7** in DMSO-d_6. Inset: ^{19}F NMR spectrum (376 MHz, 25 ℃) of **7** in DMSO-d_6. 16% of *E* and 84 % of *Z* isomers are present.

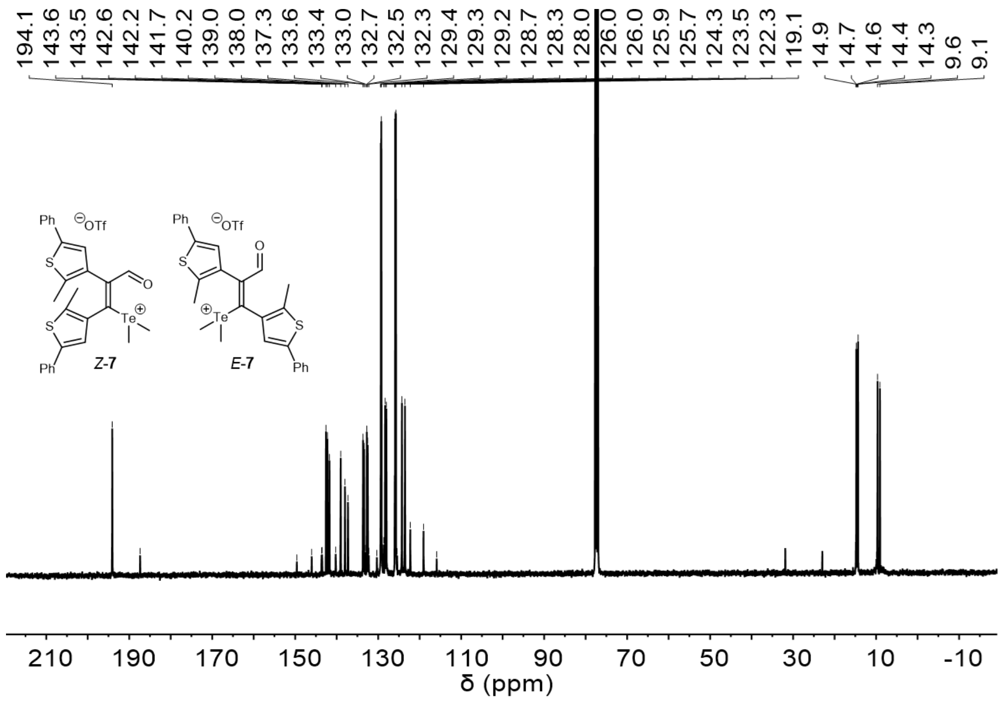

Supplementary Figure 50. ^{13}C NMR spectrum (100 MHz, 25 ℃) of **7** in $CDCl_3$. 16% of *E* and 84 % of *Z* isomers are present.

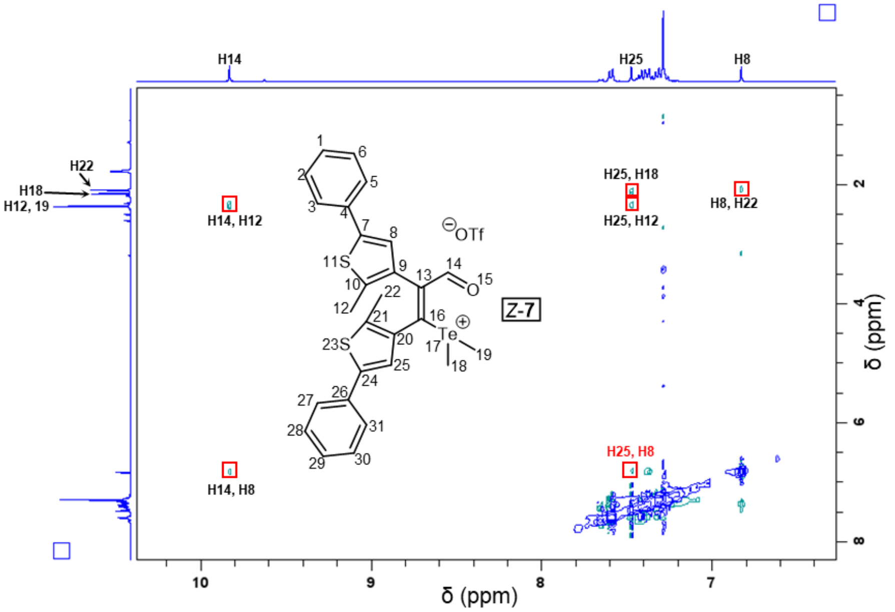

Supplementary Figure 51. Partial 2D ^{1}H-^{1}H NOESY NMR spectrum (400 MHz, 25 ℃) of **7** in $CDCl_3$. The cross signals between the protons at 7.45 ppm (H25 of *Z*-**7**) and 6.80 ppm (H8 of *Z*-**7**) confirm that the isomer is the *Z* isomer.

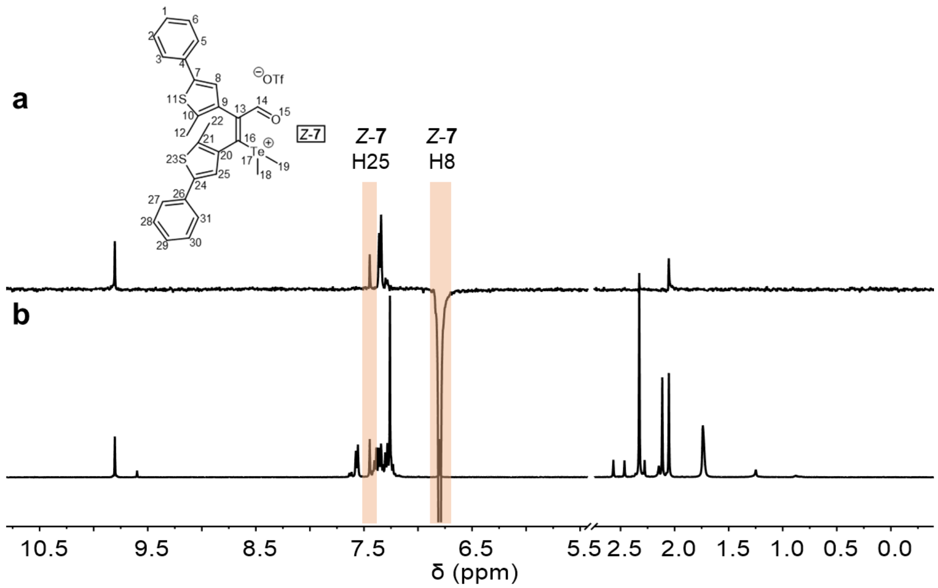

Supplementary Figure 52. 1D ^{1}H-^{1}H NOE NMR experiment of **7**. Irradiating the protons at 6.80 ppm (H8 of *Z*-**7**) leads to signal increase at 7.45 ppm (H25 of *Z*-**7**). This confirms that the isomer is the *Z* isomer. (a) 1D ^{1}H-^{1}H NOE NMR spectrum (400 MHz, $CDCl_3$, 25 ℃). (b) ^{1}H NMR spectrum (400 MHz, $CDCl_3$, 25 ℃).

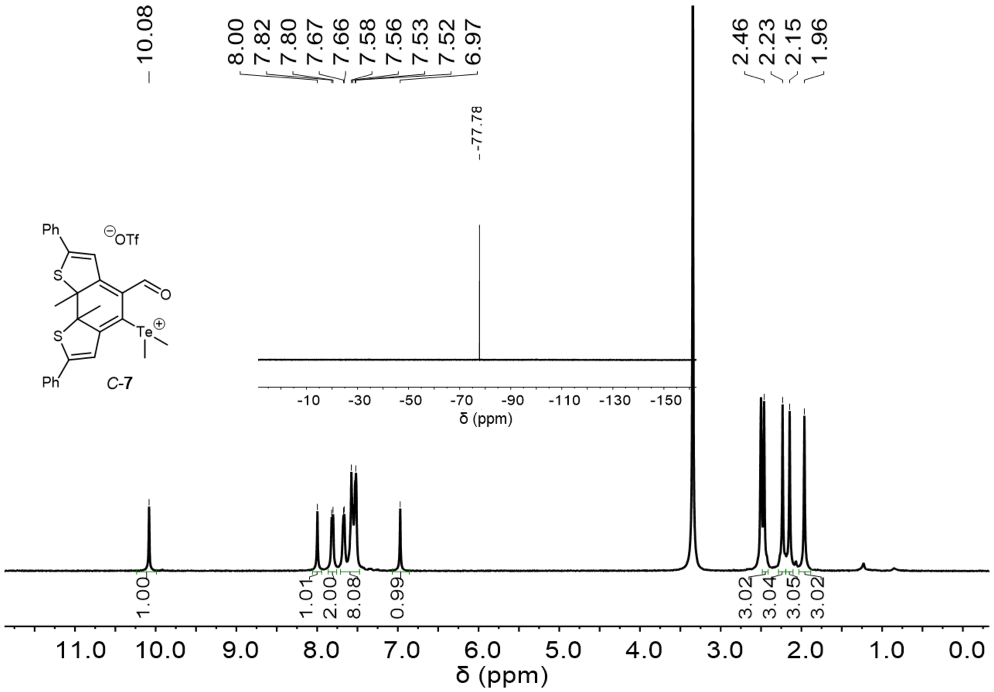

Supplementary Figure 53. ^{1}H NMR spectrum (400 MHz, 25 ℃) of *C*-**7** in DMSO-d_6. Inset: ^{19}F NMR spectrum (376 MHz, 25 ℃) of *C*-**7** in DMSO-d_6.

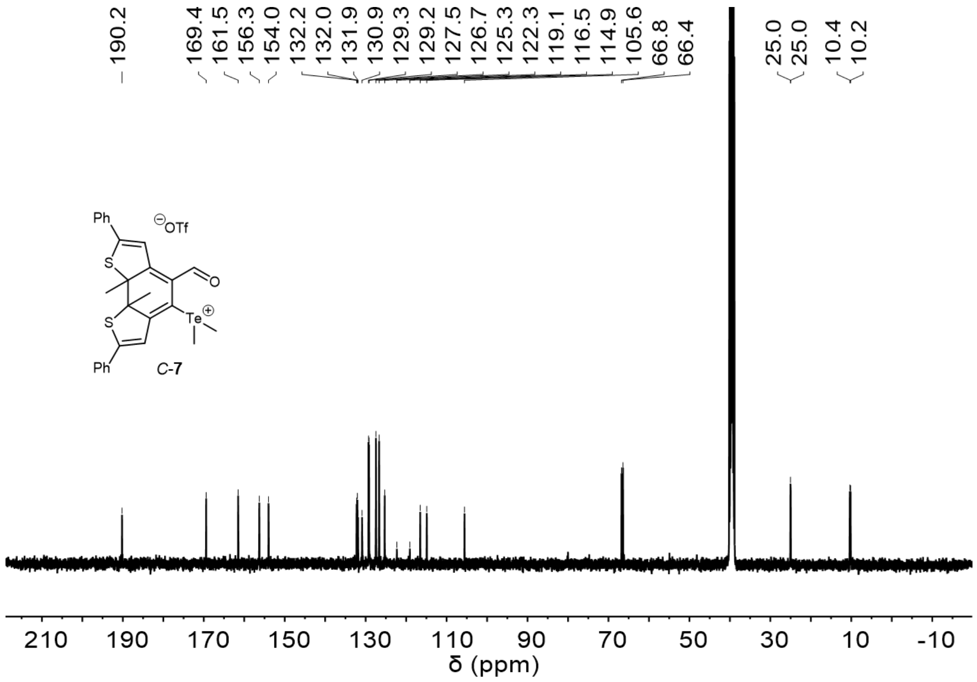

Supplementary Figure 54. ^{13}C NMR spectrum (100 MHz, 25 ℃) of *C*-**7** in DMSO-d_6.

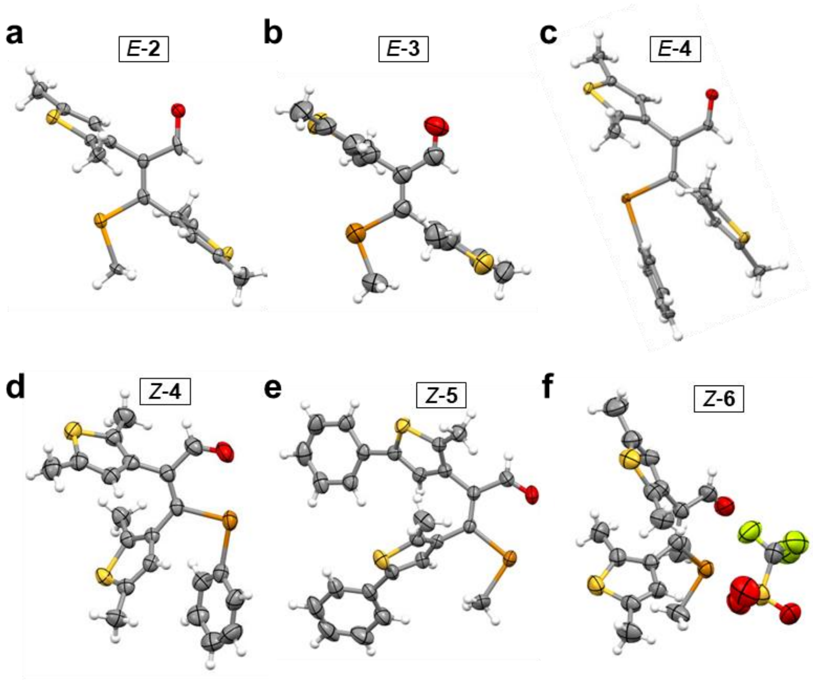

Supplementary Figure 55. Crystal structures of *E*-**2** (a), *E*-**3** (b), *E*-**4** (c), *Z*-**4** (d), *Z*-**5** (e), and *Z*-**6** (f). The thermal ellipsoids were scaled to the 50% probability level for *E*-**2**, *E*-**3**, *E*-**4**, *Z*-**4**, *Z*-**5**, and *Z*-**6**. The crystallization solvent for *E*-**2**, *E*-**3**, *Z*-**4**, and *Z*-**5** is $CHCl_3$. The crystallization solvent for *E*-**4** and *Z*-**6** is CH_3CN.

Supplementary Table 1. Summary of crystallographic data for *E*-**2**, *E*-**3**, and *E*-**4**.

	E-**2**	*E*-**3**	*E*-**4**
CCDC	2260917	2260918	2249588
Formula	$C_{16}H_{18}OS_2Se$	$C_{16}H_{18}OS_2Te$	$C_{21}H_{20}OS_2Te$
Formula weight	369.38	418.02	480.09
T / K	100	293	150
Crystallization solvent	$CHCl_3$	$CHCl_3$	CH_3CN
Color	Yellow	Yellow	Yellow
Crystal system	Triclinic	triclinic	Triclinic
Space group	*P*-1	*P*-1	*P*-1
a / Å	8.8694(4)	9.4807(3)	7.3757(2)
b / Å	8.8746(4)	12.7129(3)	9.0943(2)
c / Å	22.4929(9)	16.7679(4)	15.4231(4)
α / °	78.720(4)	71.389(2)	98.916(2)
β / °	78.705(4)	75.843(2)	95.241(2)
γ / °	82.200(4)	70.807(2)	98.120(2)
V / Å^3	1693.89(13)	1786.69(9)	1004.98(4)
Z	4	4	2
D_x / g cm^{-3}	1.449	1.554	1.587
μ / mm^{-1}	3.466	15.267	13.661
F(000)	752	824	476
θ range / °	1.77 to 60.53	2.81 to 76.85	2.92 to 72.29
GOF on F^2	1.164	1.040	1.071
R_1 [$I > 2\sigma(I)$]	0.1017	0.0482	0.0206
wR_2 (all data)	0.2405	0.1348	0.0529

Supplementary Table 2. Summary of crystallographic data for *Z*-**4**, *Z*-**5**, and *Z*-**6**.

	Z-**4**	*Z*-**5**	*Z*-**6**
CCDC	2249589	2249590	2249591
Formula	$C_{21}H_{20}OS_2Te$	$C_{26}H_{22}OS_2Te$	$C_{28}H_{25}F_3O_4S_3Te$
Formula weight	480.09	542.15	707.99
T / K	293	293	293
Crystallization solvent	$CHCl_3$	$CHCl_3$	CH_3CN
Color	Yellow	Orange	Yellow
Crystal system	monoclinic	monoclinic	Triclinic
Space group	*I*2/a	$P2_1/n$	*P*-1
a / Å	16.5926(2)	10.8843(3)	7.9152(3)
b / Å	11.85840(10)	7.7619(2)	9.0993(3)
c / Å	22.3665(3)	27.9061(7)	17.3547(6)
α / °	90	90.00	92.914(3)
β / °	110.9500(10)	94.859(2)	96.980(3)
γ / °	90	90.00	108.489(3)
V / Å^3	4109.94(9)	2349.12(11)	1171.32(7)
Z	8	4	2
D_x / g cm^{-3}	1.552	1.533	1.651
μ / mm^{-1}	13.362	11.765	12.934
$F(000)$	1904	1080	576
θ range / °	4.23 to 78.49	3.20 to 76.12	2.64 to 77.05
GOF on F^2	1.050	1.040	1.091
R_1 [$I > 2\sigma(I)$]	0.0305	0.0286	0.0455
wR_2 (all data)	0.0729	0.0713	0.1162

Supplementary Note 3. DFT Calculations

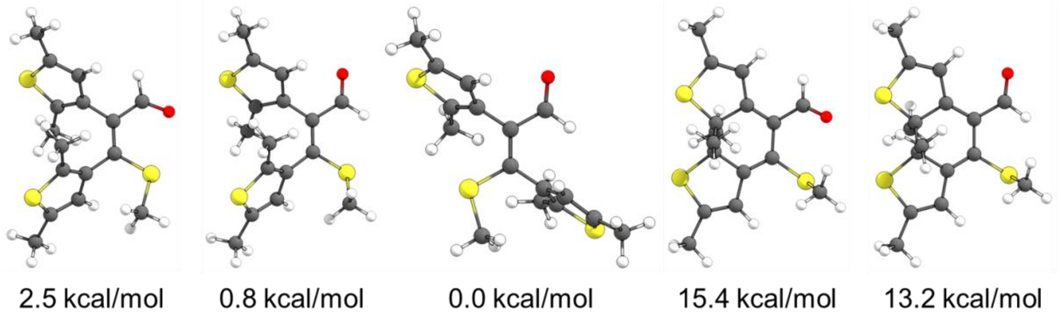

Supplementary Figure 56. Calculated structures of ***Z*-1**(isomer 1), ***Z*-1**(isomer 2), ***E*-1**, ***C*-1**(isomer 1), and ***C*-1**(isomer 2), with the relative Gibbs energy listed below.

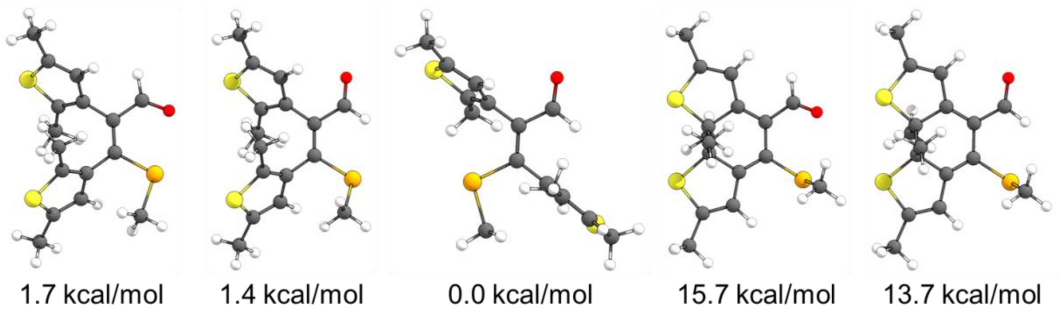

Supplementary Figure 57. Calculated structures of ***Z*-2**(isomer 1), ***Z*-2**(isomer 2), ***E*-2**, ***C*-2**(isomer 1), and ***C*-2**(isomer 2), with the relative Gibbs energy listed below.

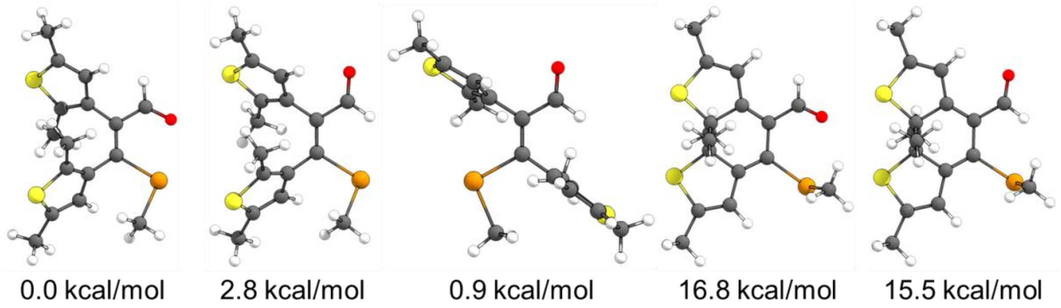

Supplementary Figure 58. Calculated structures of ***Z*-3**(isomer 1), ***Z*-3**(isomer 2), ***E*-3**, ***C*-3**(isomer 1), and ***C*-3**(isomer 2), with the relative Gibbs energy listed below.

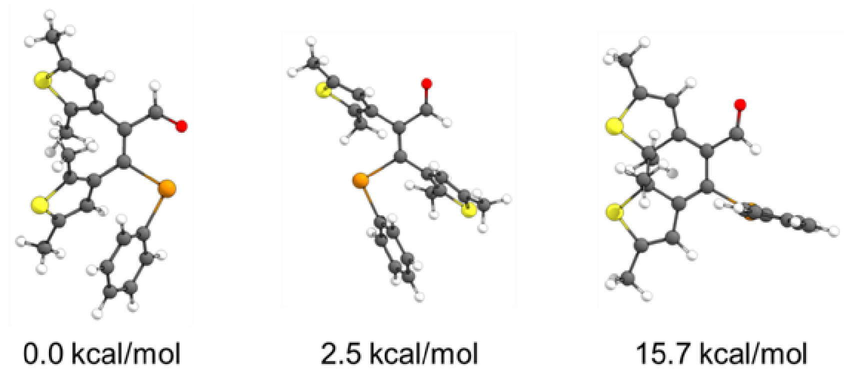

Supplementary Figure 59. Calculated structures of ***Z*-4**, ***E*-4**, and ***C*-4**, with the relative Gibbs energy listed below.

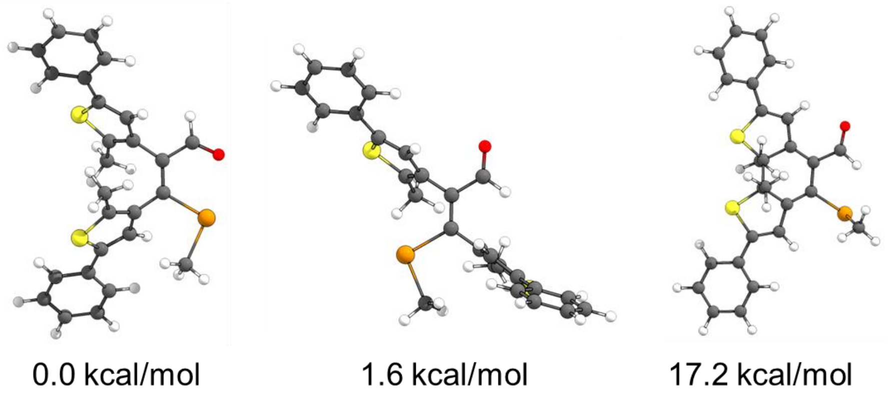

Supplementary Figure 60. Calculated structures of ***Z*-5**, ***E*-5**, and ***C*-5**, with the relative Gibbs energy listed below.

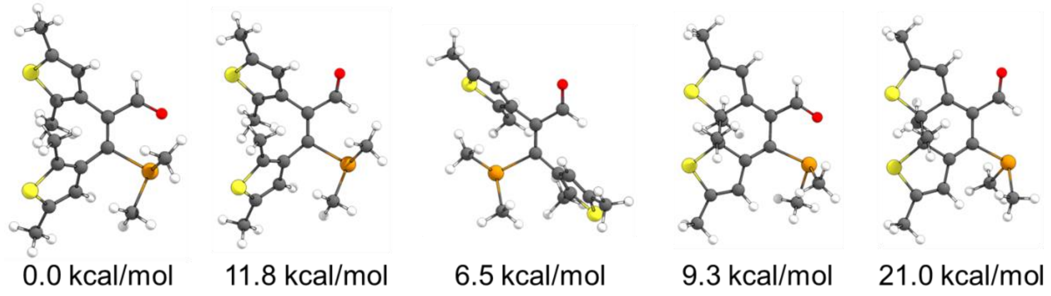

Supplementary Figure 61. Calculated structures of ***Z*-6**(isomer 1), ***Z*-6**(isomer 2), ***E*-6**, ***C*-6**(isomer 1), and ***C*-6**(isomer 2), with the relative Gibbs energy listed below.

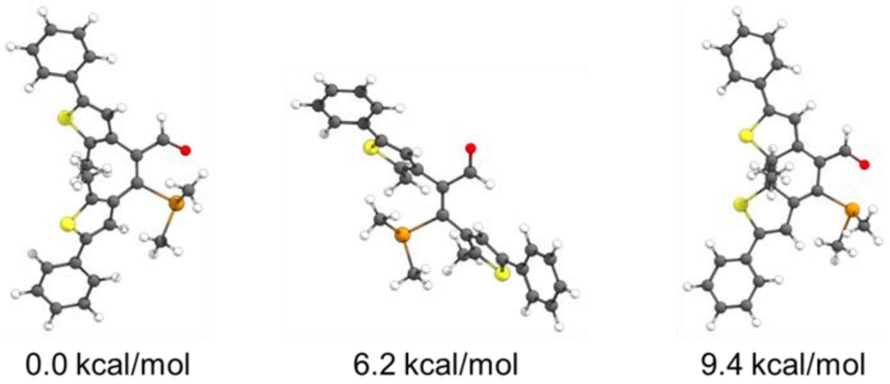

Supplementary Figure 62. Calculated structures of ***Z*-7**, ***E*-7**, and ***C*-7**, with the relative Gibbs energy listed below.

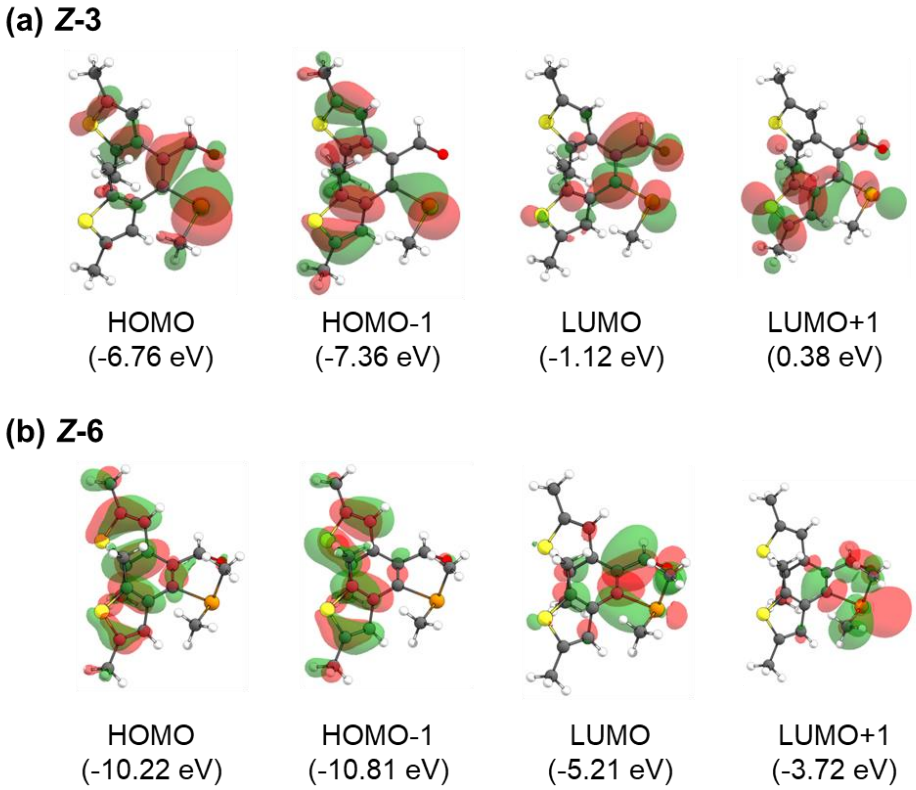

Supplementary Figure 63. The HOMO and LUMO orbitals of representative compounds ***Z*-3** (a) and ***Z*-6** (b)**.**

Supplementary Table 3. The structural and electronic properties including Len (Å), total electronic density ρ(r) (a. u.), Wiberg bond index (WBI), and n→σ* NBO stabilization energies (kcal/mol) for the atoms involved with chalcogen bonding in ***Z*** form (**1-7**).

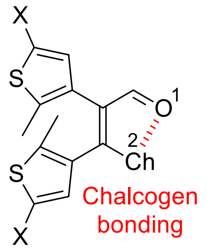

Z-form

1 X = Me Ch = SMe
2 X = Me Ch = SeMe
3 X = Me Ch = TeMe
4 X = Me Ch = TePh
5 X = Ph Ch = TeMe
6 X = Me Ch = $TeMe_2$
7 X = Ph Ch = $TeMe_2$

	**Len (Å)**	***ρ*(r)**	**WBI**	**NBO**
***Z*-1**	2.712	0.02155	0.048	3.74
***Z*-2**	2.717	0.02366	0.060	5.69
***Z*-3**	2.695	0.02813	0.091	9.55
***Z*-4**	2.672	0.02930	0.095	10.52
***Z*-5**	2.698	0.02798	0.091	9.47
***Z*-6**	2.592	0.03531	0.080	11.35
***Z*-7**	2.596	0.03503	0.079	11.19

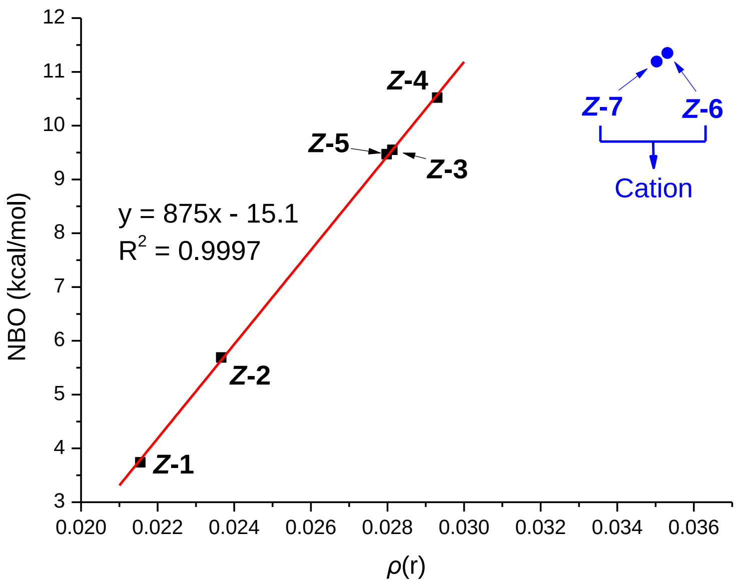

Supplementary Figure 64. Correlation between total electronic density ρ(r) and n→σ* NBO energies (kcal/mol) of chalcogen bonding in ***Z*** form (**1-7**).

Supplementary Table 4. WBI and the length (Len, Å) for the bonds involved with chalcogen bonding in ***Z*** form (**1-7**), as well as the structural features without chalcogen bonding in ***E*** form (**1-7**).

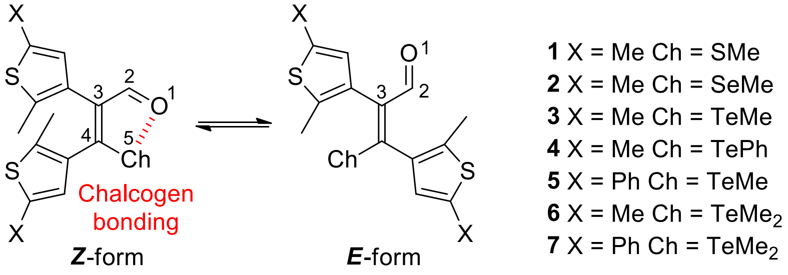

***Z*-1**	**WBI**	**Len (Å)**	***E*-1**	**WBI**	**Len (Å)**
O1-C2	1.796	1.208	O1-C2	1.813	1.206
C2-C3	1.059	1.473	C2-C3	1.037	1.478
C3-C4	1.624	1.362	C3-C4	1.679	1.353
C4-S5	1.141	1.757	C4-S5	1.109	1.757
S5-C6	1.008	1.810	S5-C6	1.022	1.804
***Z*-2**	**WBI**	**Len (Å)**	***E*-2**	**WBI**	**Len (Å)**
O1-C2	1.782	1.209	O1-C2	1.820	1.205
C2-C3	1.061	1.472	C2-C3	1.027	1.480
C3-C4	1.638	1.359	C3-C4	1.707	1.349
C4-Te5	1.100	1.910	C4-Se5	1.060	1.911
Te5-C6	0.983	1.957	Se5-C6	1.005	1.949
***Z*-3**	**WBI**	**Len (Å)**	***E*-3**	**WBI**	**Len (Å)**
O1-C2	1.742	1.214	O1-C2	1.825	1.205
C2-C3	1.076	1.466	C2-C3	1.019	1.482
C3-C4	1.641	1.359	C3-C4	1.735	1.346
C4-Te5	1.033	2.107	C4-Te5	0.986	2.112
Te5-C6	0.927	2.152	Te5-C6	0.973	2.139
***Z*-4**	**WBI**	**Len (Å)**	***E*-4**	**WBI**	**Len (Å)**
O1-C2	1.738	1.214	O1-C2	1.827	1.204
C2-C3	1.076	1.466	C2-C3	1.015	1.483
C3-C4	1.646	1.358	C3-C4	1.743	1.345
C4-Te5	1.013	2.120	C4-Te5	0.963	2.127
Te5-C6	0.891	2.131	Te5-C6	0.937	2.116

***Z*-5**	**WBI**	**Len (Å)**	***E*-5**	**WBI**	**Len (Å)**
O1-C2	1.744	1.214	O1-C2	1.826	1.203
C2-C3	1.075	1.466	C2-C3	1.018	1.487
C3-C4	1.641	1.359	C3-C4	1.735	1.348
C4-Te5	1.034	2.106	C4-Te5	0.987	2.118
Te5-C6	0.928	2.152	Te5-C6	0.974	2.139
***Z*-6**	**WBI**	**Len (Å)**	***E*-6**	**WBI**	**Len (Å)**
O1-C2	1.758	1.211	O1-C2	1.892	1.196
C2-C3	1.028	1.487	C2-C3	0.961	1.508
C3-C4	1.729	1.348	C3-C4	1.808	1.339
C4-Te5	0.890	2.149	C4-Te5	0.894	2.142
Te5-C6	0.932	2.124	Te5-C6	0.966	2.116
***Z*-7**	**WBI**	**Len (Å)**	***E*-7**	**WBI**	**Len (Å)**
O1-C2	1.758	1.211	O1-C2	1.890	1.196
C2-C3	1.028	1.486	C2-C3	0.962	1.508
C3-C4	1.729	1.348	C3-C4	1.809	1.338
C4-Te5	0.891	2.148	C4-Te5	0.893	2.142
Te5-C6	0.933	2.124	Te5-C6	0.966	2.117

Supplementary Note 4. Photoswitching Studies of Neutral Compounds

Supplementary Note 4.1 Photoswitching Experiments

Supplementary Table 5. Summary of the distribution at the photostationary state of chalcogen-containing photoswitches for *E/Z/C* isomers of **1-5**.

Compound	Ch<br>X	Solvent	λ_{light} (nm)	*E*[a] (%)	*Z*[a] (%)	*C*[a] (%)
E-**1**	SMe<br>Me	DMSO-d_6	313	30	21	49
			535[b]	30	70	0
		$CDCl_3$	313	88	3	9
			535[b]	97	3	0
E-**2**	SeMe<br>Me	DMSO-d_6	313	33	43	24
			535[b]	33	67	0
		$CDCl_3$	313	79	5	16
			535[b]	95	5	0
E-**3**	TeMe<br>Me	DMSO-d_6	365	4	96	/[c]
			450[b]	45	55	
		$CDCl_3$	365	4	96	/[c]
			450[b]	42(47)[d]	58(53)[d]	
E-**4**	TePh<br>Me	DMSO-d_6	365	11	89	/[c]
			450[b]	62	38	
		$CDCl_3$	365	10	90	/[c]
			450[b]	67	33	
E-**5**	TeMe<br>Ph	DMSO-d_6	365	3	96	1
			450[b]	39	60	1
		$CDCl_3$	365	3	96	1
			450[b]	40	59	1

[a] After irradiation with different light determined by NMR. [b] After UV light illumination of *E* isomer reached the PSS, the target light source was used for irradiation until reaching the PSS. [c] Not observed. [d] When light illumination experiment was carried out at 120 ℃, the ratio of *E*:*Z* is 47:53.

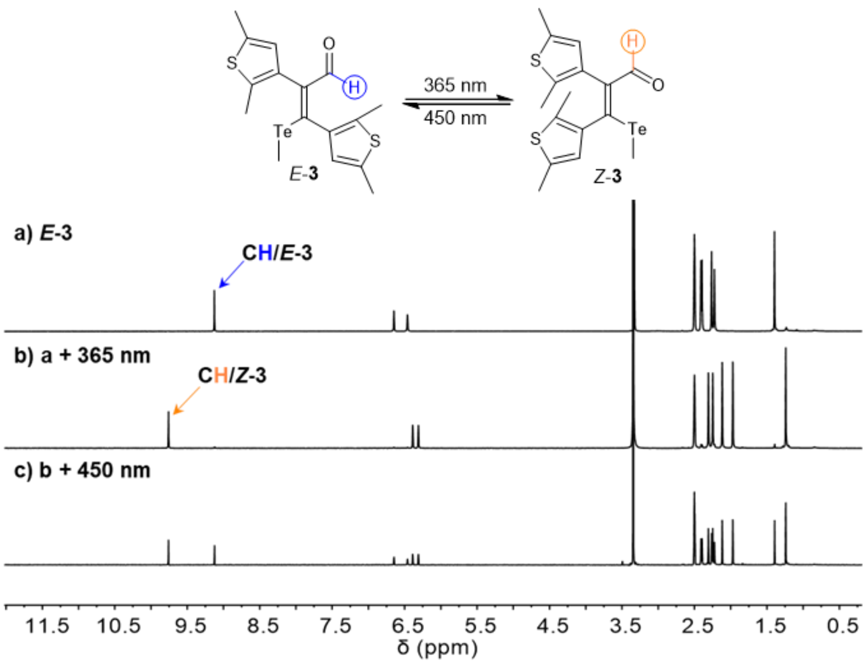

Supplementary Figure 65. (a) ^{1}H NMR spectrum of *E*-**3** (10 mM) in DMSO-d_6 at 25 °C; (b) Irradiation of *E*-**3** with UV light (365 nm, 24 min). The ratio of *E*:*Z* is 4:96; (c) Further irradiation with visible light (450 nm, 40 min). The ratio of *E*:*Z* is 45:55.

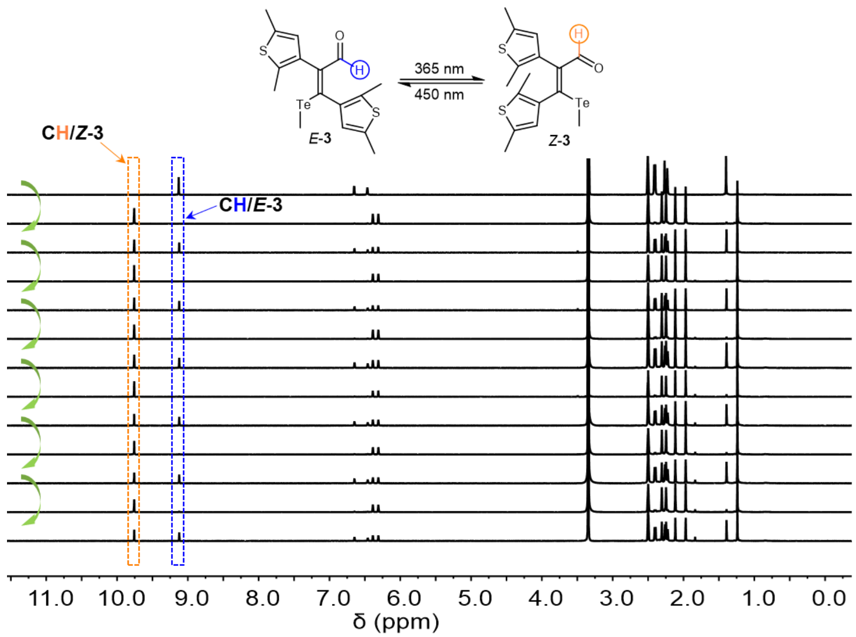

Supplementary Figure 66. ^{1}H NMR spectra of *Z*-**3** (10 mM) in DMSO-d_6 at 25 °C during repetitive photoswitching cycles consisting of alternating visible (450 nm, 40 min) and UV (365 nm, 24 min) light.

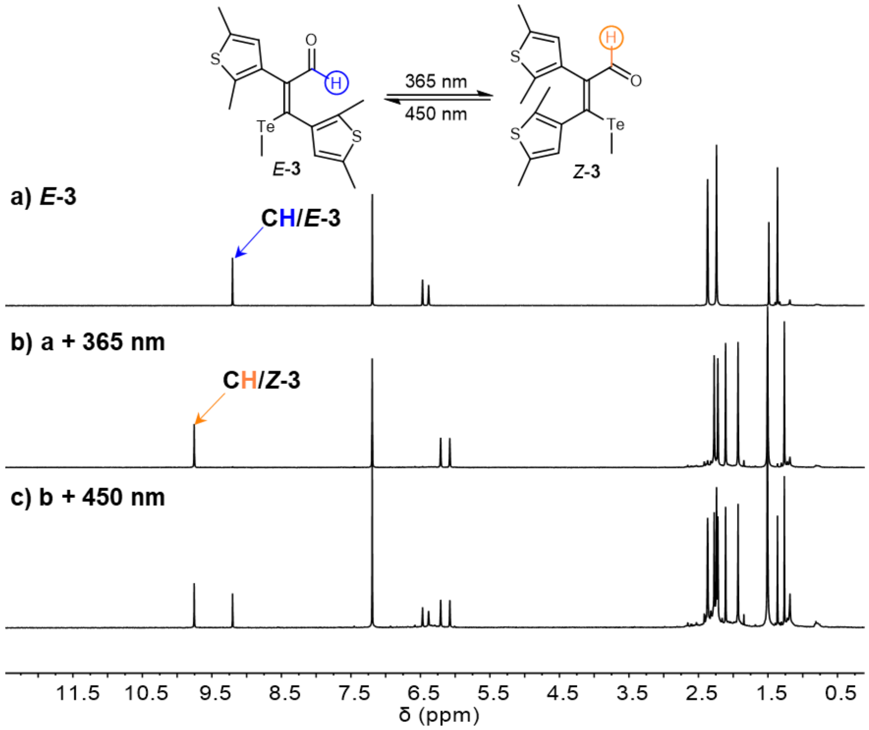

Supplementary Figure 67. (a) ^{1}H NMR spectrum of *E*-**3** (10 mM) in $CDCl_3$ at 25 °C; (b) Irradiation of *E*-**3** with UV light (365 nm, 25 min). The ratio of *E*:*Z* is 4:96; (c) Further irradiation with visible light (450 nm, 50 min). The ratio of *E*:*Z* is 42:58.

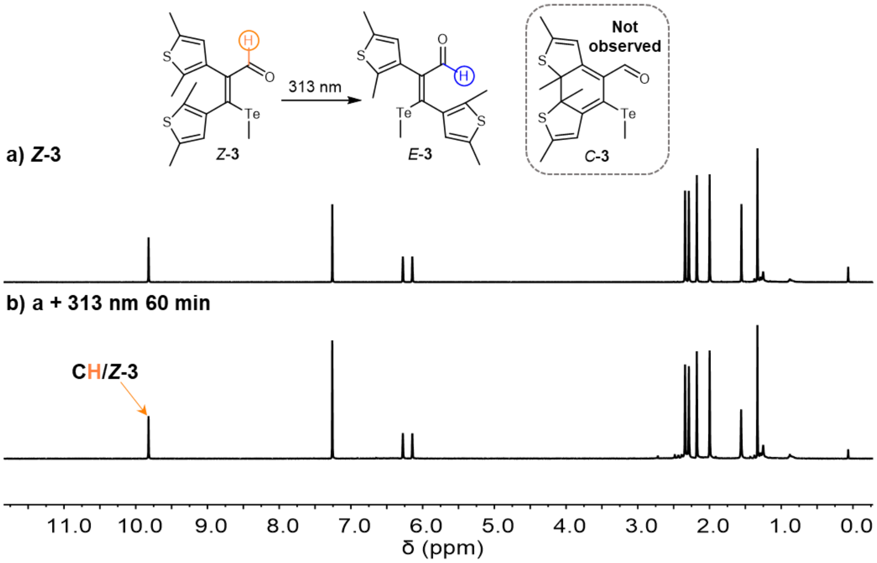

Supplementary Figure 68. (a) ^{1}H NMR spectrum of *Z*-**3** (10 mM, 25 ℃) in $CDCl_3$; (b) Irradiation of *Z*-**3** with UV light (313 nm, 60 min). The ratio of *E*:*Z* is 2:98.

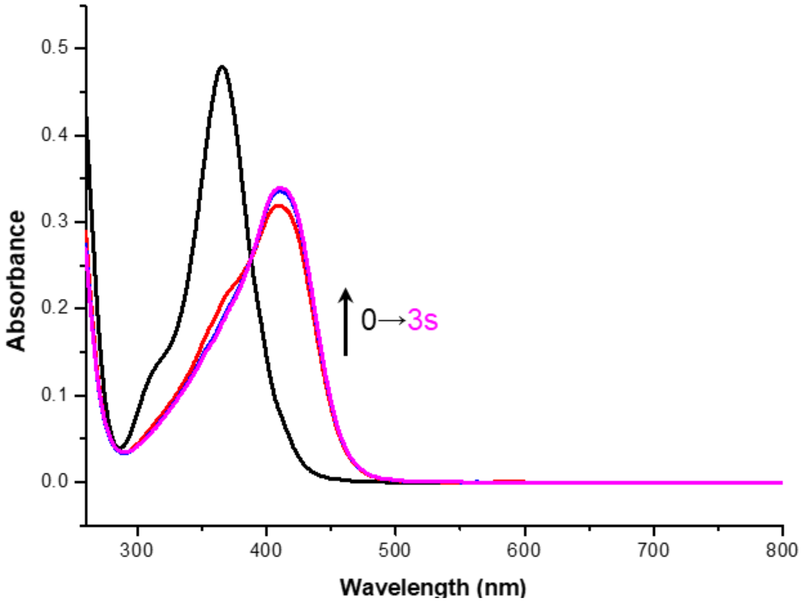

Supplementary Figure 69. UV-vis absorption spectra of *E*-**3** (60 μM in DMSO) in the pristine state and upon irradiation with 365 nm light after total irradiation of 0, 1, 2, and 3 s. The photostationary state was reached after 3 s.

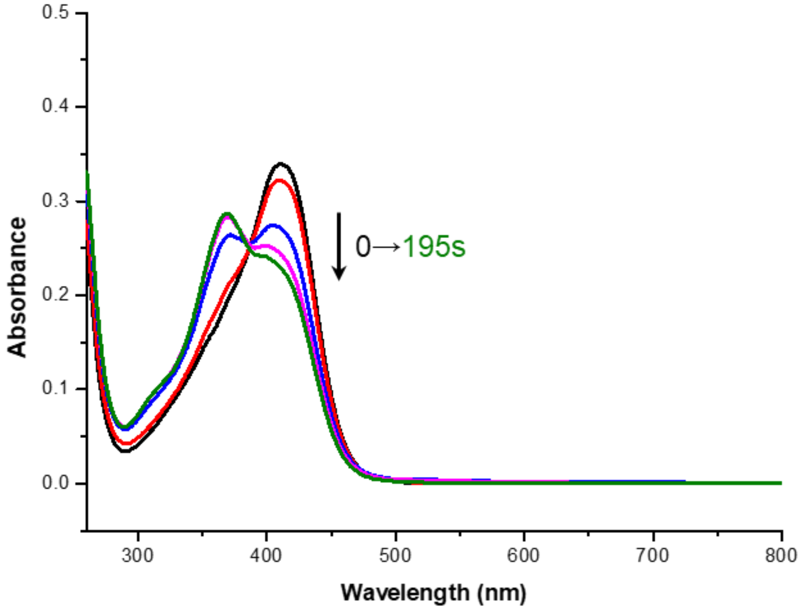

Supplementary Figure 70. UV-vis absorption spectra of *E*-**3** (60 μM in DMSO) at the PSS (365 nm) and upon irradiation with 450 nm light after total irradiation of 0, 18, 60, 155, and 195 s. The photostationary state was reached after 195 s.

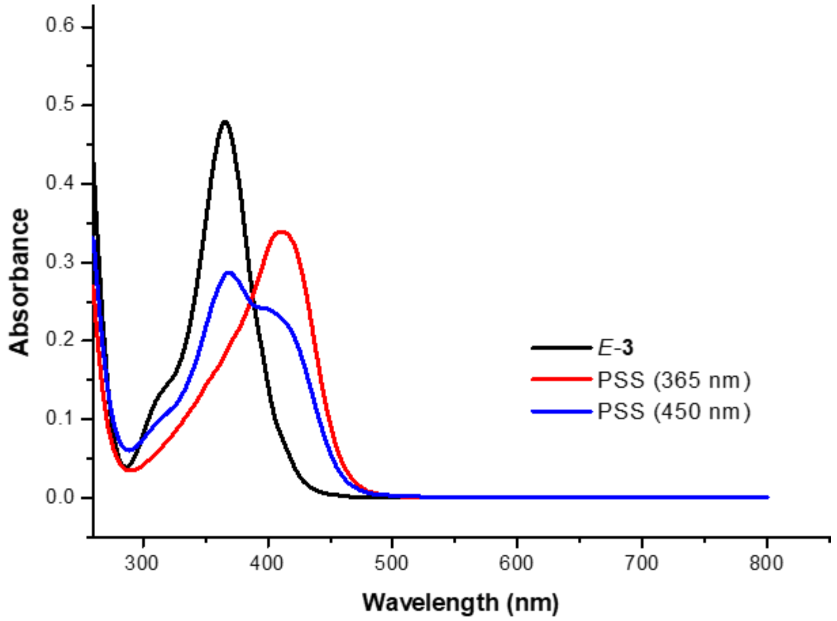

Supplementary Figure 71. UV-vis absorption spectra of *E*-**3** (60 μM in DMSO) in the pristine state and at the PSS upon irradiation with different wavelength of light (365 then 450 nm).

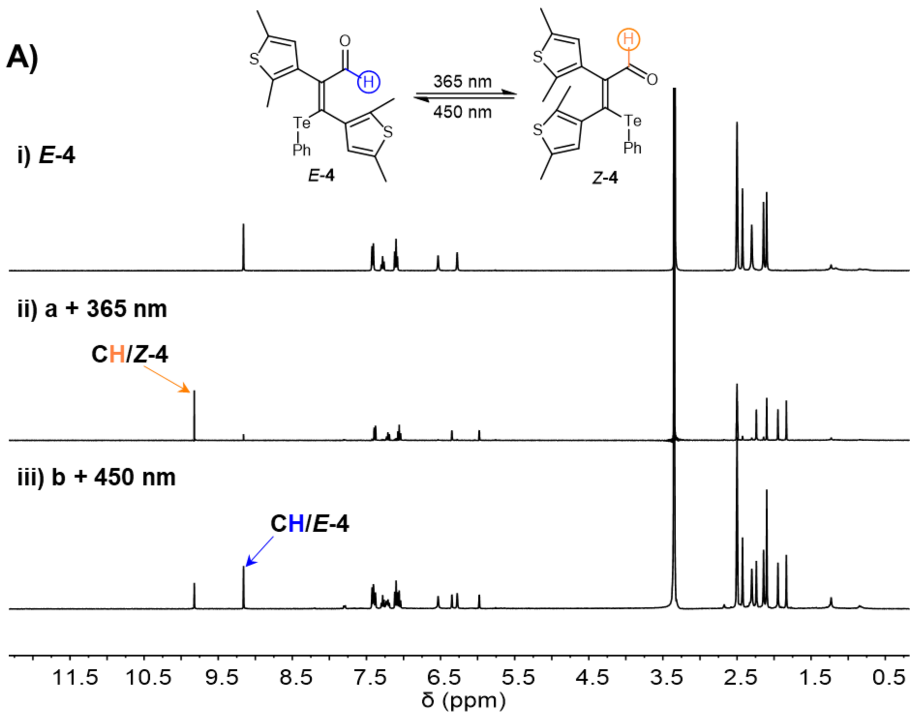

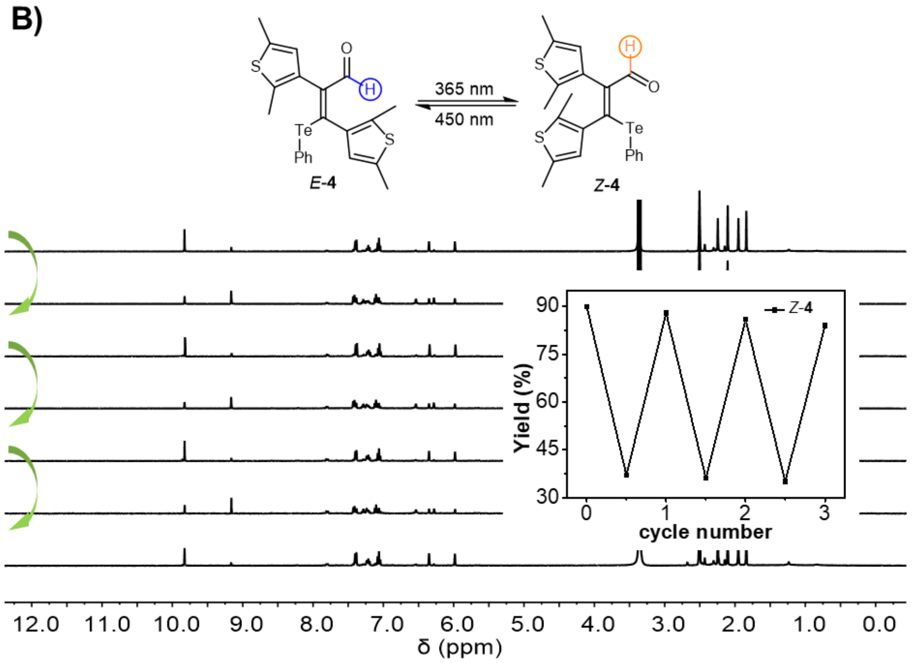

Supplementary Figure 72. (**A**) (Ai) ^{1}H NMR spectrum of *E*-**4** (10 mM) in DMSO-d_6 at 25 °C; (Aii) Irradiation of *E*-**4** with UV light (365 nm, 30 min). The ratio of *E*:*Z* is 11:89; (Aiii) Further irradiation with visible light (450 nm, 70 min). The ratio of *E*:*Z* is 62:38. (**B**) ^{1}H NMR spectra of **4** (10 mM) in DMSO-d_6 at 25 °C during repetitive photoswitching cycles consisting of alternating visible (450 nm, 70 min) and UV (365 nm, 30 min) light.

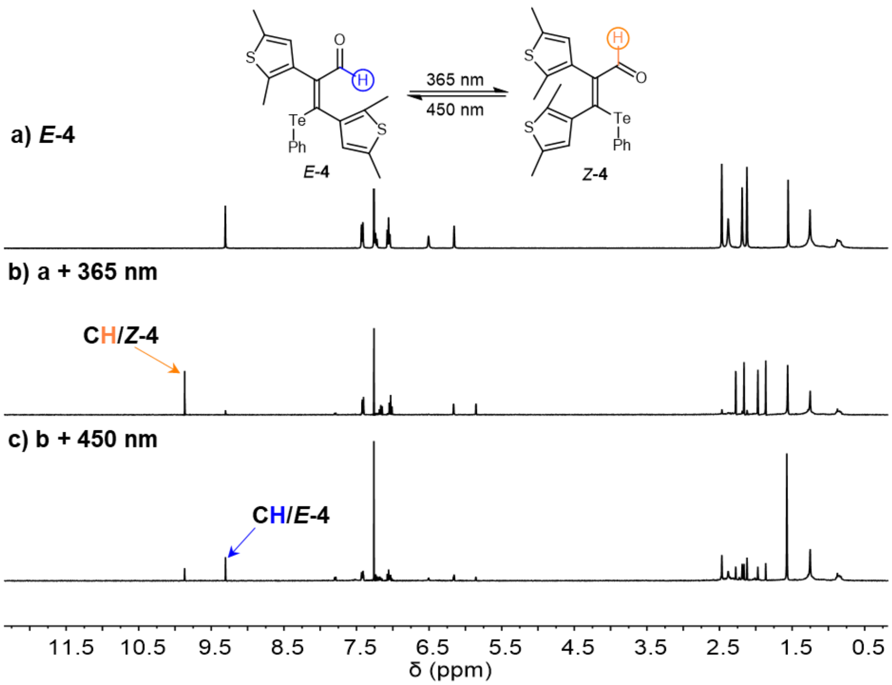

Supplementary Figure 73. (a) ^{1}H NMR spectrum of *E*-**4** (10 mM) in $CDCl_3$ at 25 °C; (b) Irradiation of *E*-**4** with UV light (365 nm, 25 min). The ratio of *E*:*Z* is 10:90; (c) Further irradiation with visible light (450 nm, 50 min). The ratio of *E*:*Z* is 67:33.

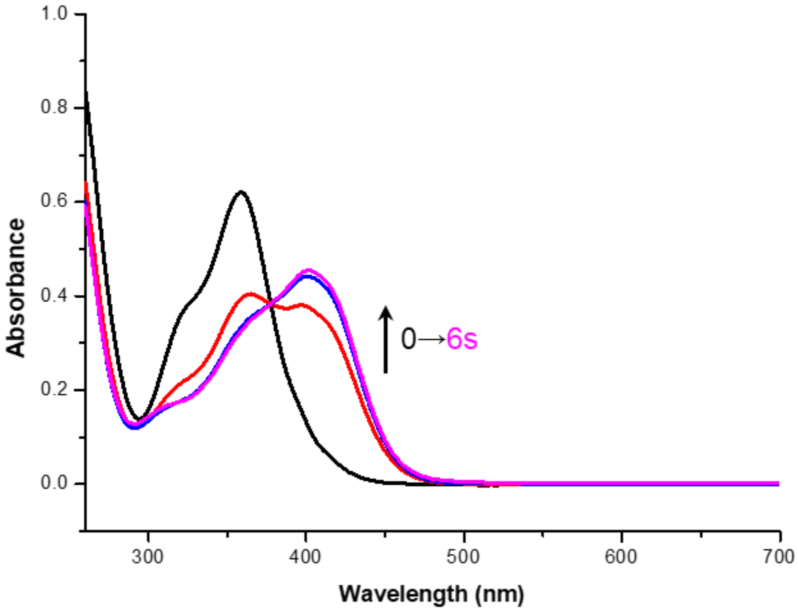

Supplementary Figure 74. UV-vis absorption spectra of *E*-**4** (60 μM in DMSO) in the pristine state and upon irradiation with 365 nm light after total irradiation of 0, 2, 4, and 6 s. The photostationary state was reached after 6 s.

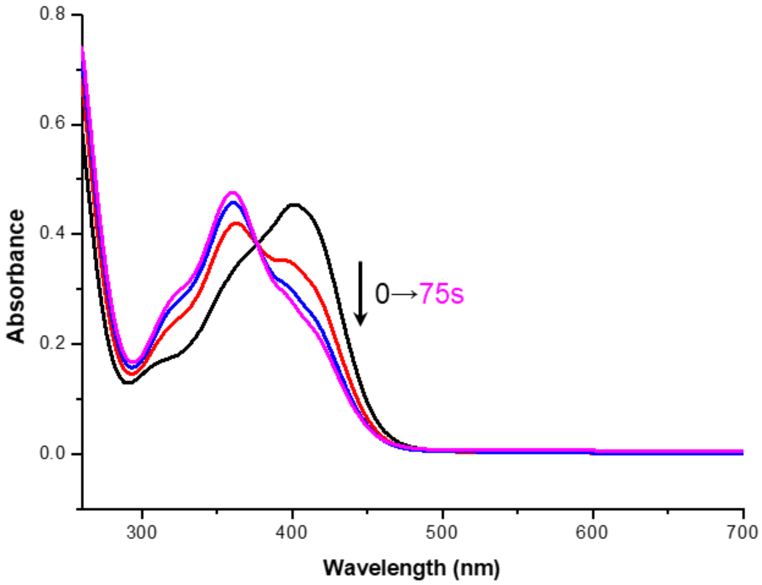

Supplementary Figure 75. UV-vis absorption spectra of *E*-**4** (60 μM in DMSO) at the PSS (365 nm) and upon irradiation with 450 nm light after total irradiation of 0, 25, 49, and 75 s. The photostationary state was reached after 75 s.

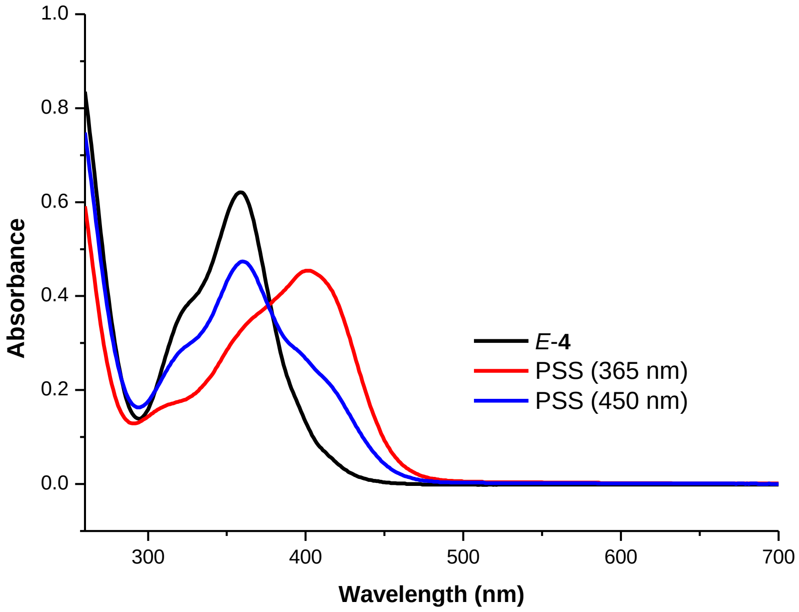

Supplementary Figure 76. UV-vis absorption spectra of *E*-**4** (60 μM in DMSO) in the pristine state and at the PSS upon irradiation with different wavelength of light (365 then 450 nm).

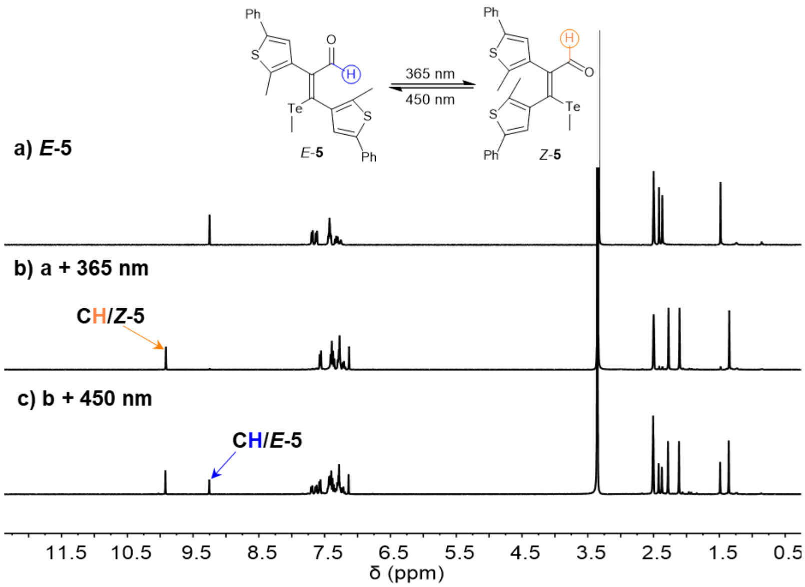

Supplementary Figure 77. (a) ^{1}H NMR spectrum of *E*-**5** (10 mM) in DMSO-d_6 at 25 °C; (b) Irradiation of *E*-**5** with UV light (365 nm, 30 min). The ratio of *E*:*Z*:*C* is 3:96:1; (c) Further irradiation with visible light (450 nm, 60 min). The ratio of *E*:*Z*:*C* is 39:60:1.

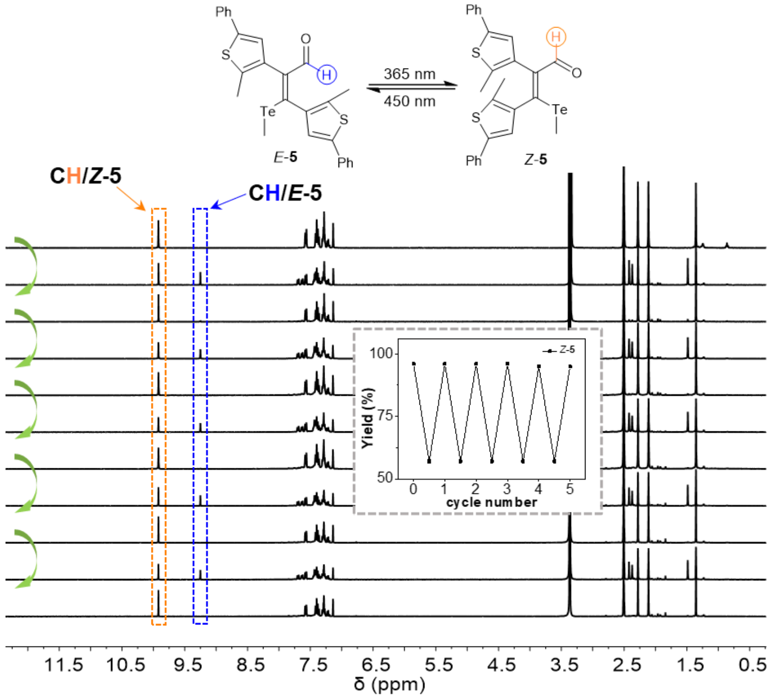

Supplementary Figure 78. ^{1}H NMR spectra of *Z*-**5** (10 mM) in DMSO-d_6 at 25 °C during repetitive photoswitching cycles consisting of alternating visible (450 nm, 60 min) and UV (365 nm, 25 min) light.

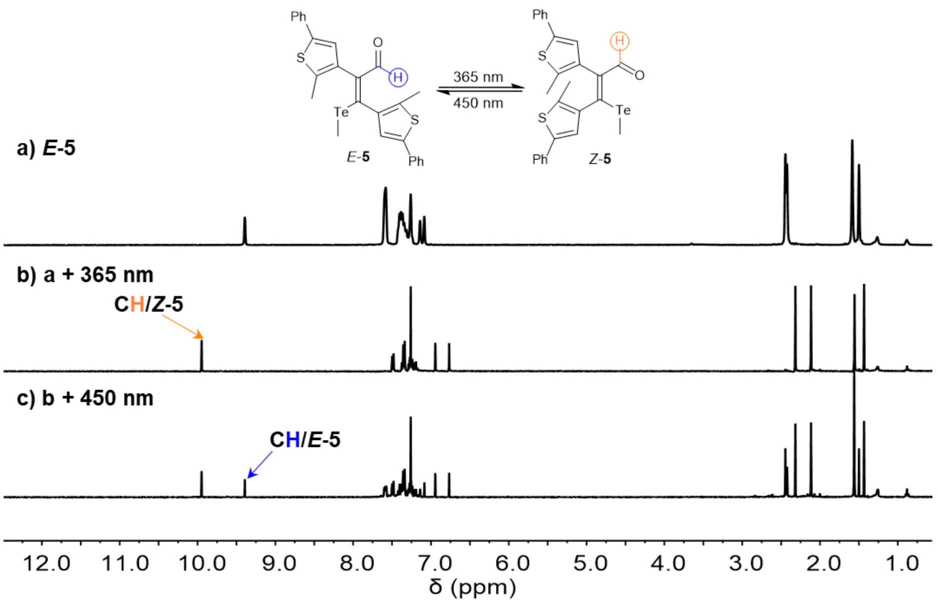

Supplementary Figure 79. (a) ^{1}H NMR spectrum of *E*-**5** (10 mM) in $CDCl_3$ at 25 °C; (b) Irradiation of *E*-**5** with UV light (365 nm, 25 min). The ratio of *E*:*Z* is *E*:*Z*:*C* is 3:96:1; (c) Further irradiation with visible light (450 nm, 50 min). The ratio of *E*:*Z*:*C* is 40:59:1.

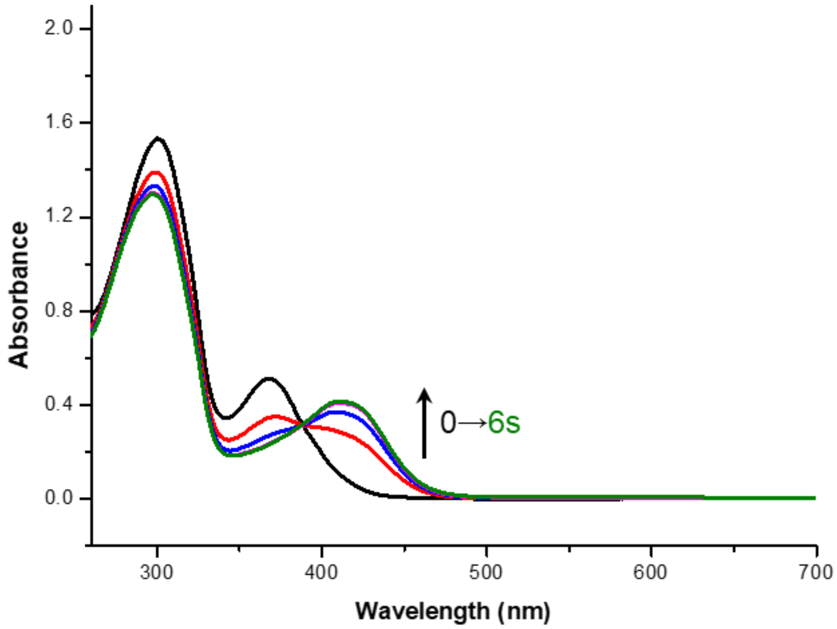

Supplementary Figure 80. UV-vis absorption spectra of *E*-**5** (60 μM in DMSO) in the pristine state and upon irradiation with 365 nm light after total irradiation of 0, 1, 2, 4, and 6 s. The photostationary state was reached after 6 s.

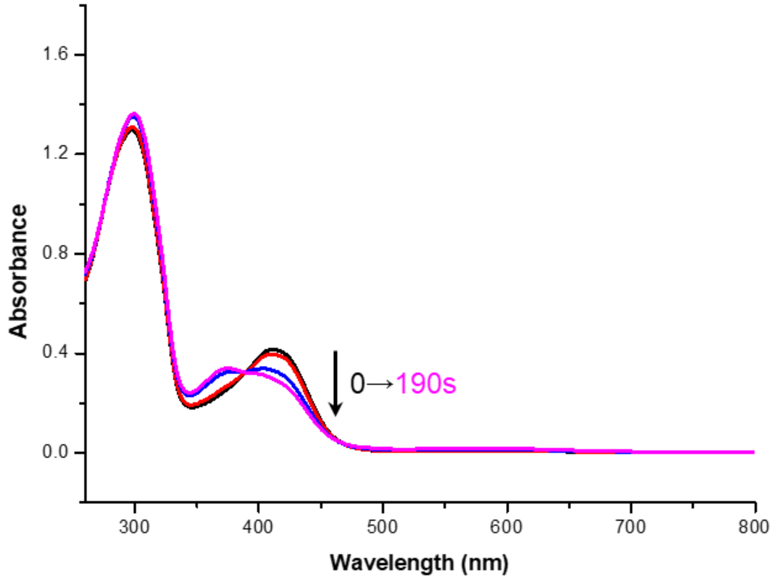

Supplementary Figure 81. UV-vis absorption spectra of *E*-**5** (60 μM in DMSO) at the PSS (365 nm) and upon irradiation with 450 nm light after total irradiation of 0, 40, 80, and 190 s. The photostationary state was reached after 190 s.

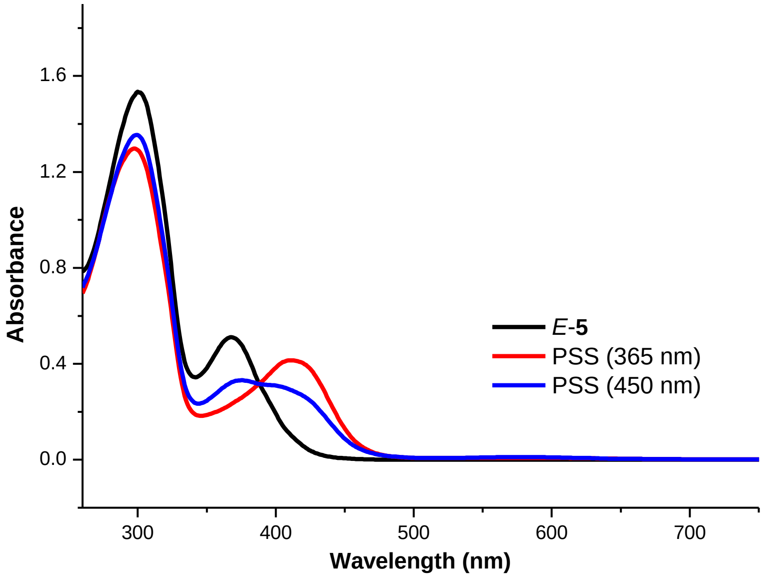

Supplementary Figure 82. UV-vis absorption spectra of *E*-**5** (60 μM in DMSO) in the pristine state and at the PSS upon irradiation with different wavelength of light (365 then 450 nm).

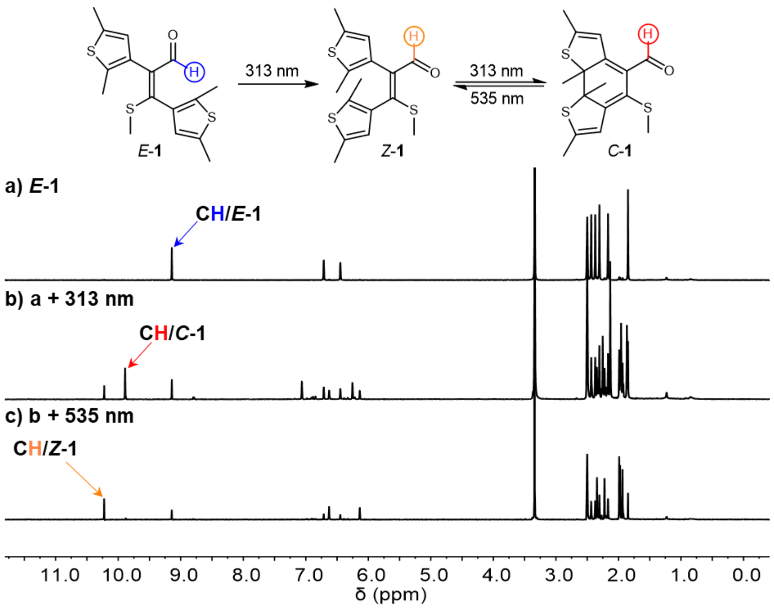

Supplementary Figure 83. (a) ^{1}H NMR spectrum of *E*-**1** (10 mM) in DMSO-d_6 at 25 °C; (b) Irradiation of *E*-**1** with UV light (313 nm, 120 min). The ratio of *E*:*Z*:*C* is 30:21:49; (c) Further irradiation with visible light (535 nm, 50 min). The ratio of *E*:*Z* is 30:70.

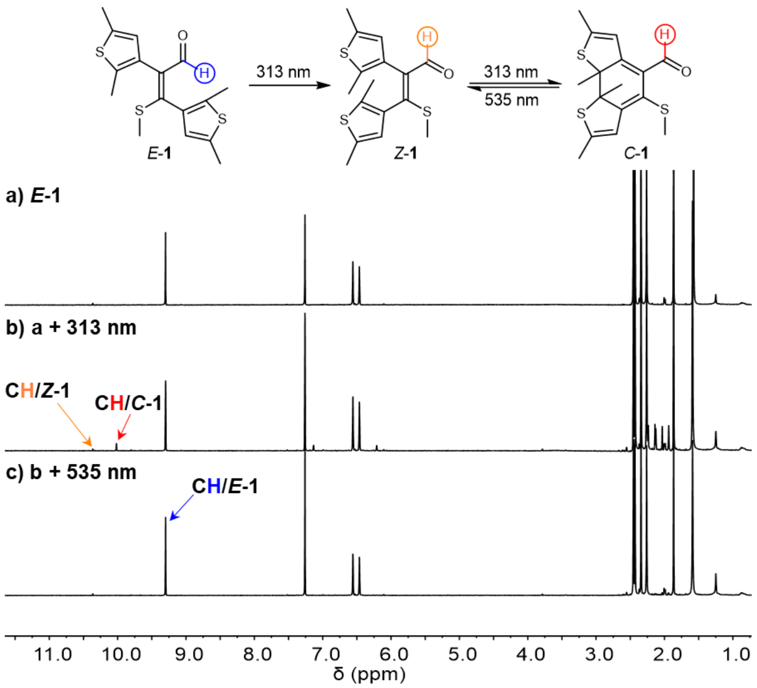

Supplementary Figure 84. (a) ^{1}H NMR spectrum of *E*-**1** (10 mM) in $CDCl_3$ at 25 °C; (b) Irradiation of *E*-**1** with UV light (313 nm, 50 min). The ratio of *E*:*Z*:*C* is 88:3:9; (c) Further irradiation with visible light (535 nm, 30 min). The ratio of *E*:*Z* is 97:3.

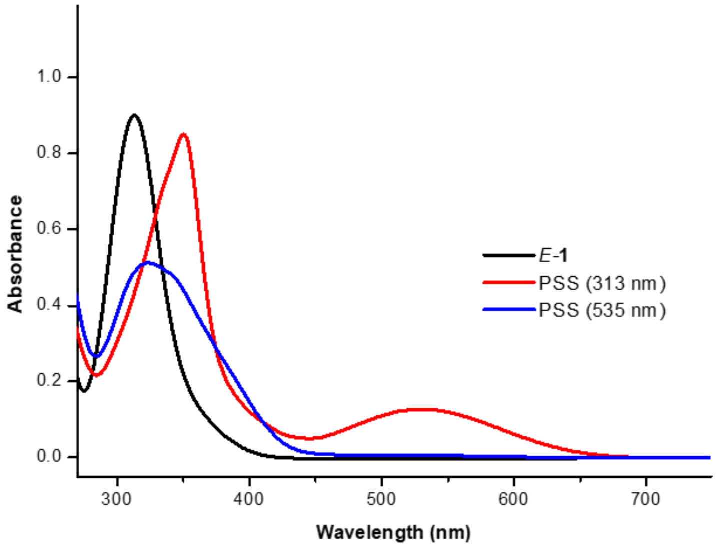

Supplementary Figure 85. UV-vis absorption spectra of *E*-**1** (60 μM in DMSO) in the pristine state and at the PSS upon irradiation with different wavelength of light (313 then 535 nm).

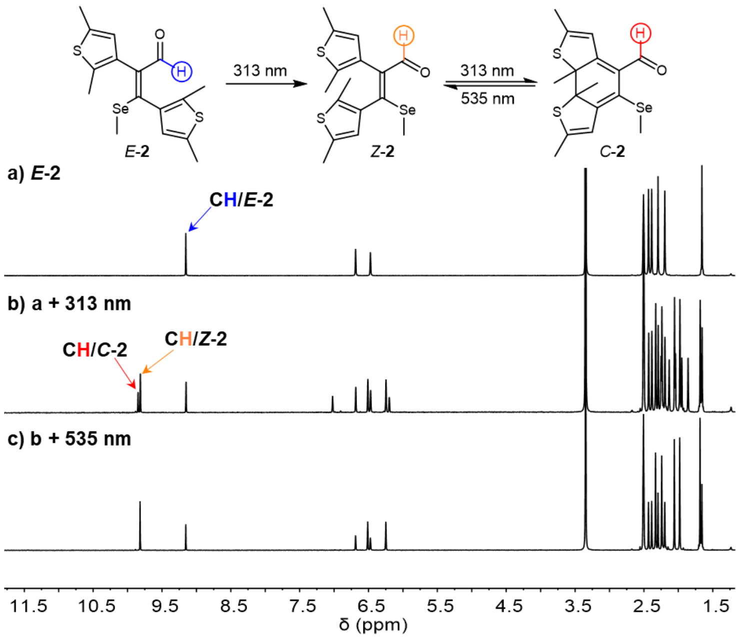

Supplementary Figure 86. (a) ^{1}H NMR spectrum of *E*-**2** (10 mM) in DMSO-d_6 at 25 ℃; (b) Irradiation of *E*-**2** with UV light (313 nm, 90 min). The ratio of *E*:*Z*:*C* is 33:43:24; (c) Further irradiation with visible light (535 nm, 30 min). The ratio of *E*:*Z* is 33:67.

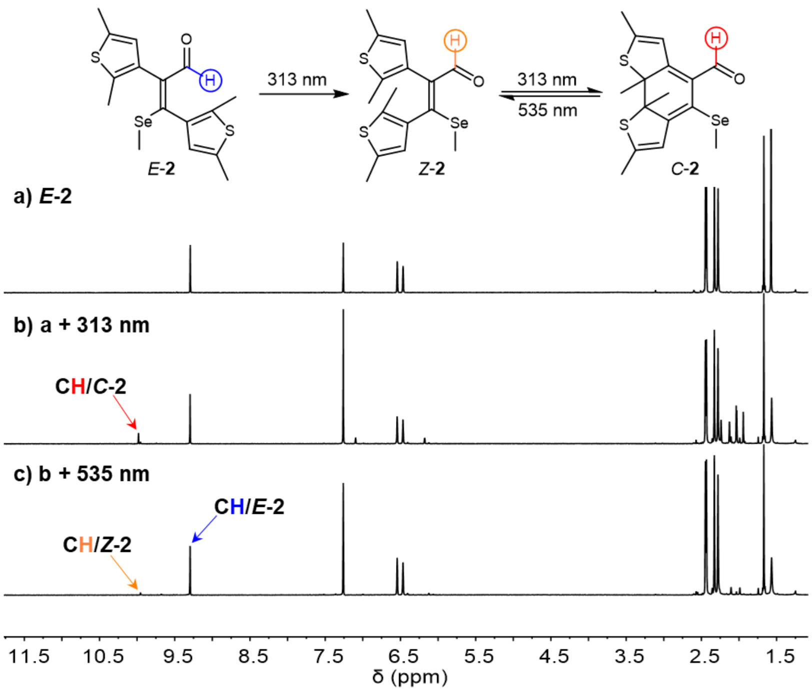

Supplementary Figure 87. (a) ^{1}H NMR spectrum of *E*-**2** (10 mM) in $CDCl_3$ at 25 °C; (b) Irradiation of *E*-**2** with UV light (313 nm, 70 min). The ratio of *E*:*Z*:*C* is 79:5:16; (c) Further irradiation with visible light (535 nm, 20 min). The ratio of *E*:*Z* is 95:5.

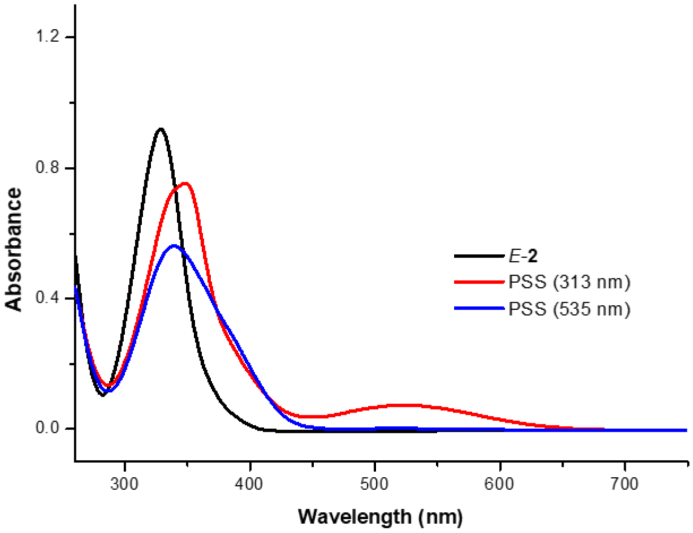

Supplementary Figure 88. UV-vis absorption spectra of *E*-**2** (60 µM in DMSO) in the pristine state and at the PSS upon irradiation with different wavelength of light (313 then 535 nm).

Molar absorption coefficient. The molar absorption coefficients *ε* of *E*/*Z*/*C* isomers were obtained directly from isomerically pure material by using the Lambert-Beer law.

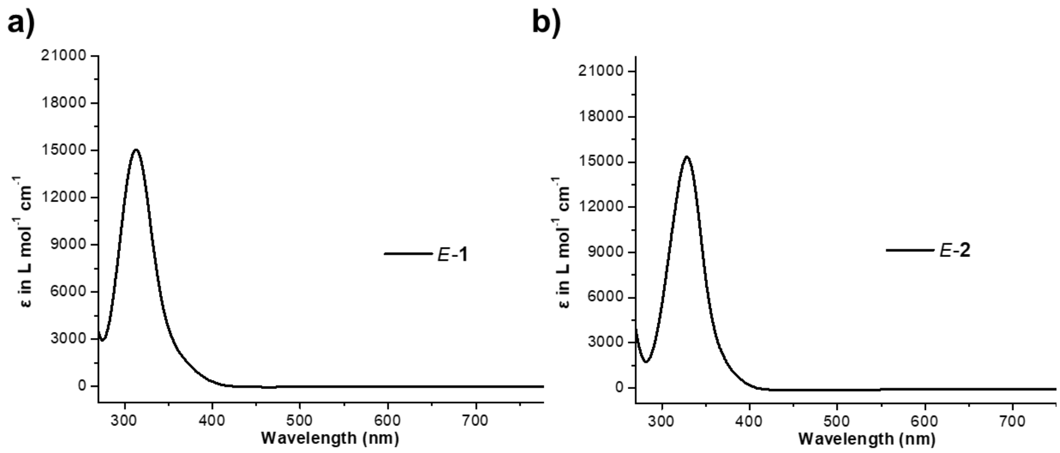

Supplementary Figure 89. Molar absorption coefficients *ε* of (a) *E*-**1**, (b) *E*-**2** in DMSO at 25 ℃.

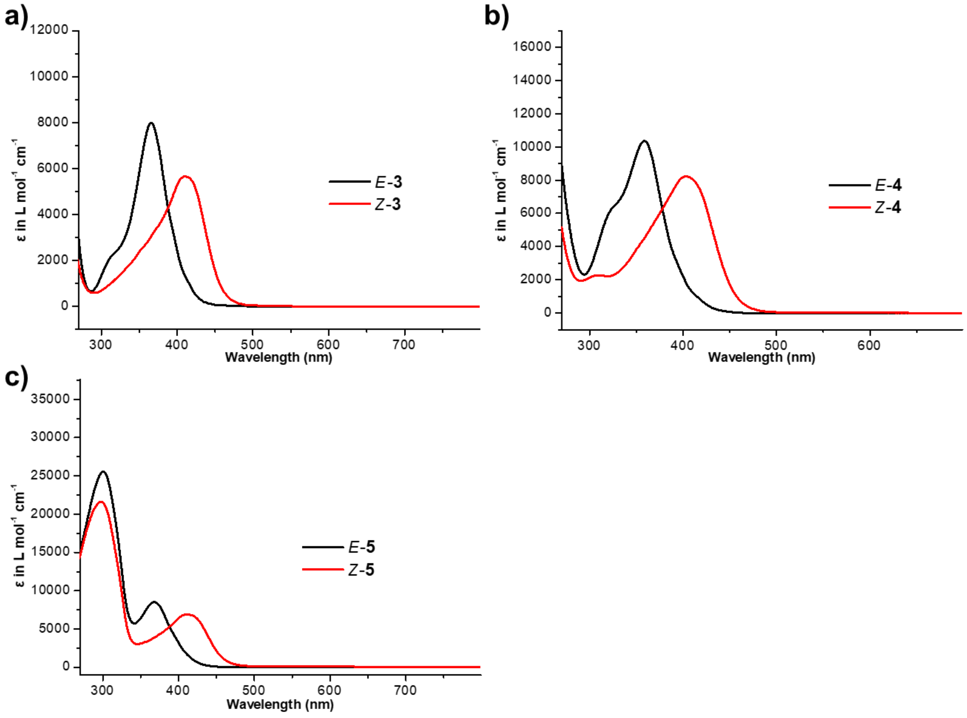

Supplementary Figure 90. Molar absorption coefficients *ε* of (a) **3**, (b) **4**, **(**c) **5** in DMSO at 25 ℃. *Z* isomers are displayed in red, and *E* isomers are displayed in black.

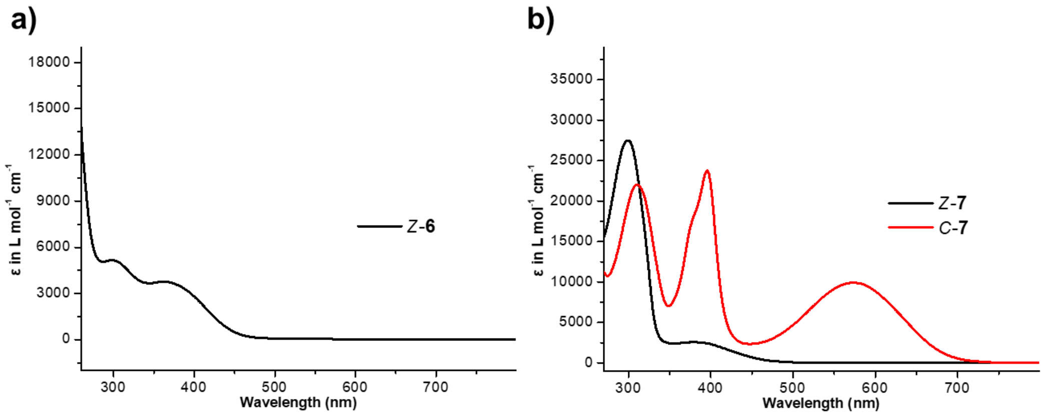

Supplementary Figure 91. Molar absorption coefficients ε of (a) **6**, (b) **7** in DMSO at 25 ℃. *C* isomer is displayed in red, and *Z* isomers are displayed in black.

Determination of quantum yields. The extinction coefficients of the open/closed diarylethene isomers were determined upon precise preparation of stock solutions with defined concentrations and subsequent measurement of the absorption spectra.

The light intensity at 313 nm and 405 nm was determined by using ferrioxalate actinometry.[3] Therefore 3 mL of the ferrioxalate actinometry solution (0.006 M) in 0.05 M H_2SO_4 was irradiated, and 0.5 mL of phenanthroline (0.1 wt% in 0.5 M H_2SO_4/1.6 M NaOAc) were added subsequently. The resulting absorbance at 510 nm was used to calculate the light intensity via formula (1):

$$I_0 = \frac{\Delta A_{510nm}}{\Delta t * \varepsilon_{510nm} * \Phi_{irr} * 1000} * \frac{3.5ml}{3mL} \quad \textbf{(1)}$$

ΔA_{510nm} is the difference of the absorption at 510 nm for an irradiated versus a nonirradiated solution, Δt is the irradiation time, ε_{510nm} is 11100 M^{-1} cm^{-1}, and Φ_{irr} is the quantum yield at the used irradiation wavelength (1.24 for 313 nm, 1.21 for 365 nm, and 1.11 for 425 nm). For the ring-opening a wavelength was needed that exceed the range for ferrioxalate actinometry. Therefore actinometry via aberchrome 670 was performed.[4] A toluene solution of aberchrome 670 (0.0001 M) was first irradiated with 365 nm. The formed isomer was irradiated back with the wavelength of interest. The decay of the band at 519 nm indicates the light intensity via formula (2):

$$I_0 = \frac{\Delta A_{519nm}}{\Delta t * \varepsilon_{519nm} * \Phi_{irr} * 1000 * (1-10^{-A'})} \quad (2)$$

ΔA_{519nm} is the difference of the absorption at 519 nm for an irradiation interval Δt. ε_{519nm} is 7760 M^{-1} cm^{-1}, and Φ_{irr} is the quantum yield at the used irradiation wavelength 546 nm (0.28).

For the determination of the quantum yield formula (3) was used:

$$\Phi = \frac{\Delta A / \Delta t}{\left(1-10^{-A'}\right) * \varepsilon * I_0 * 1000} \quad (3)$$

$\Delta A/\Delta t$ is the change of absorbance at a wavelength, which changes upon irradiation within the time. $1 - 10^{-A'}$ is the percentage of the absorbed photons by the solution at irradiation wavelength, ε is the extinction coefficient at observed wavelength, and I_0 the light intensity.

Supplementary Table 6. Experimentally determined molar absorption coefficients (ε) and quantum yields (Φ) for the photoisomerization of isomers in DMSO solution at 25 ℃.

Compound	ε (L mol^{-1} cm^{-1})	$\Phi_{E\to Z+C}$ (%)	$\Phi_{E\to Z}$ (%)	$\Phi_{Z\to E}$ (%)	$\Phi_{Z\to C+E}$ (%)	$\Phi_{C\to Z}$ (%)
E-**1**	15033(313 nm)	0.12	/	/	/	/
E-**2**	10983(313 nm)	0.03	/	/	/	/
E-**3**	7983 (365 nm)	/	11.9	0.45	/	/
Z-**3**	1550 (450 nm)					
E-**4**	9750 (365 nm)	/	3.50	1.07	/	/
Z-**4**	1716 (450 nm)					
E-**5**	8433 (365 nm)	/	7.85	0.39	/	/
Z-**5**	2166 (450 nm)					
Z-**6**	1470 (425 nm)	/	/	/	1.87	/
Z-**7**	1500 (425 nm)	/	/	/	1.73	1.21
C-**7**	8860 (600 nm)					

Supplementary Note 4.2 Thermal Experiments

Thermal isomerziation. At elevated temperatures in the dark metastable *Z* isomers thermally convert to corresponding *E* isomers. The thermal *Z* to *E* isomerization kinetics was determined by heating samples enriched in metastable *Z* isomer in an oil-bath and monitoring the conversion process by ^{1}H NMR spectroscopy at 25 ℃ in selected time intervals.

To calculate the relative energy difference (ΔG) between the two isomeric states (*Z* and *E*), the equilibrium constant *K* must be determined at a given temperature and can then be inserted into formula (4) ($K = ([Z]/[E])$, $[Z]$ the concentration of the *Z* isomer in thermal equilibrium and $[E]$ the concentration of the *E* isomer in thermal equilibrium).

$$-\Delta G = ln(K)\ R\ T \qquad \mathbf{(4)}$$

R = ideal gas constant (8.314 $J \cdot K^{-1} \cdot mol^{-1}$)

T = temperature in K

Half-life times $t_{1/2}$ of *Z* isomers and relative energy differences ΔG between the two isomeric states of photoswitches **1-5** are given in Table S7.

Supplementary Table 7. Half-life times $t_{1/2}$ of the *Z* isomers of photoswitches **1-5** at the indicated temperatures and relative energy differences Δ*G* between the two isomeric states. Thermal *Z* to *E* isomerization reactions were measured in DMSO-d_6 at 80 or 125 ℃. Isomeric ratios *E*:*Z* after thermal isomerization is finished as well as the rates of thermal isomerization were determined by ^{1}H NMR spectroscopy.

Compounds	solvent	Δ*G* / kcal mol^{-1}	$t_{1/2}$ (h, T)	*E*:*Z* (%)
**1** (*E*/*Z* = 30:70)	DMSO-d_6	2.1	2.4 h, 353 K	95:5
**2** (*E*/*Z* = 33:67)	DMSO-d_6	1.9	8.6 h, 398 K	91:9
Z-**3**	DMSO-d_6	0.32	49 h, 398 K	60:40
Z-**4**	DMSO-d_6	0.16	48 h, 398 K	55:45
Z-**5**[a]	DMSO-d_6	/	/	/

[a] decomposition during heating.

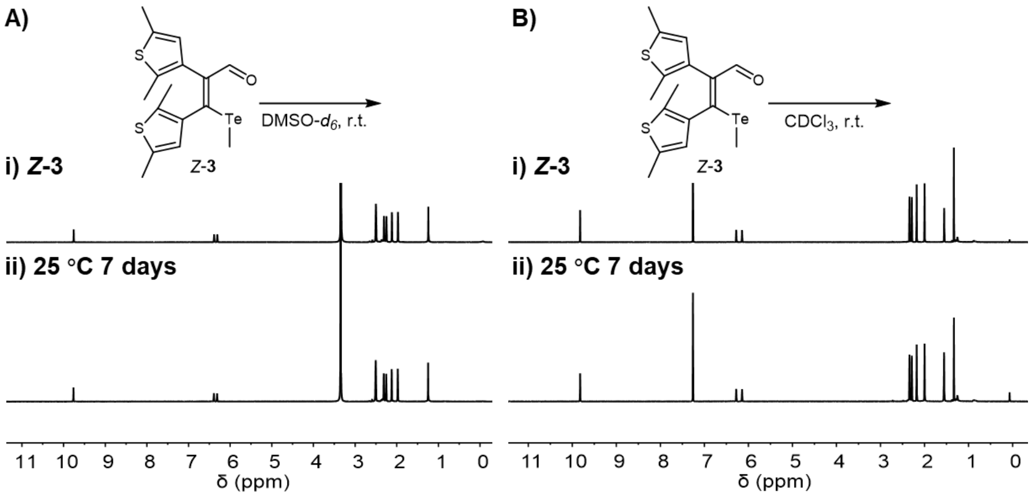

Supplementary Figure 92. (**A**) (Ai) ^{1}H NMR spectrum of *Z*-**3** (10 mM) in DMSO-d_6; (Aii) Waiting at 25 ℃ for 7 days. (**B**) (Bi) ^{1}H NMR spectrum of *Z*-**3** (10 mM) in $CDCl_3$; (Bii) Waiting at 25 ℃ for 7 days.

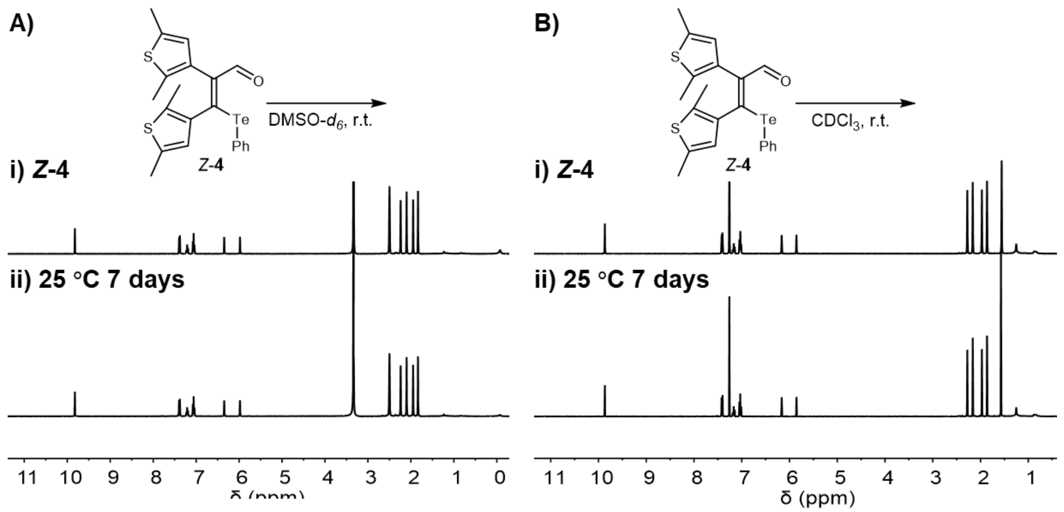

Supplementary Figure 93. (**A**) (Ai) ^{1}H NMR spectrum of *Z*-**4** (10 mM) in DMSO-d_6; (Aii) Waiting at 25 ℃ for 7 days. (**B**) (Bi) ^{1}H NMR spectrum of *Z*-**4** (10 mM) in $CDCl_3$; (Bii) Waiting at 25 ℃ for 7 days.

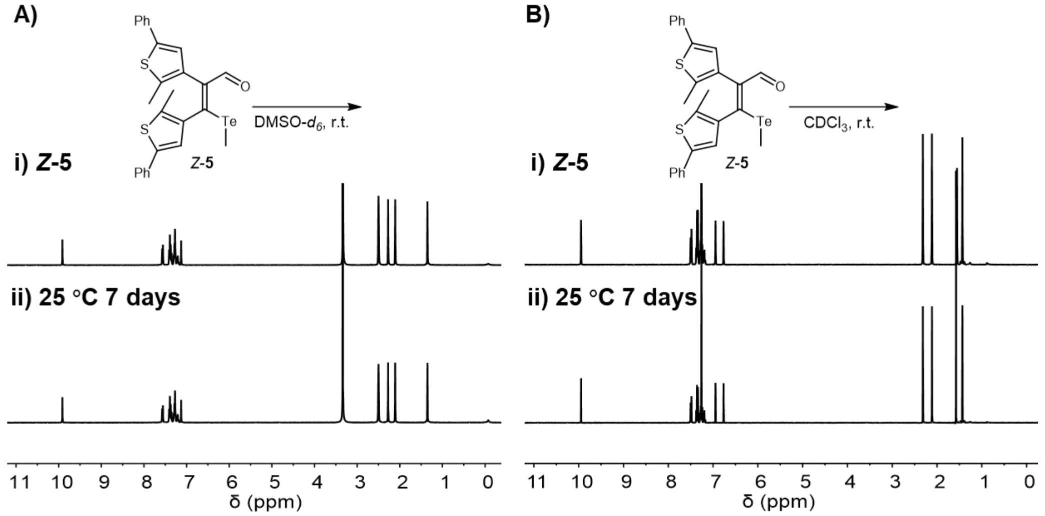

Supplementary Figure 94. (**A**) (Ai) ^{1}H NMR spectrum of *Z*-**5** (10 mM) in DMSO-d_6; (Aii) Waiting at 25 ℃ for 7 days. (**B**) (Bi) ^{1}H NMR spectrum of *Z*-**5** (10 mM) in $CDCl_3$; (Bii) Waiting at 25 ℃ for 7 days.

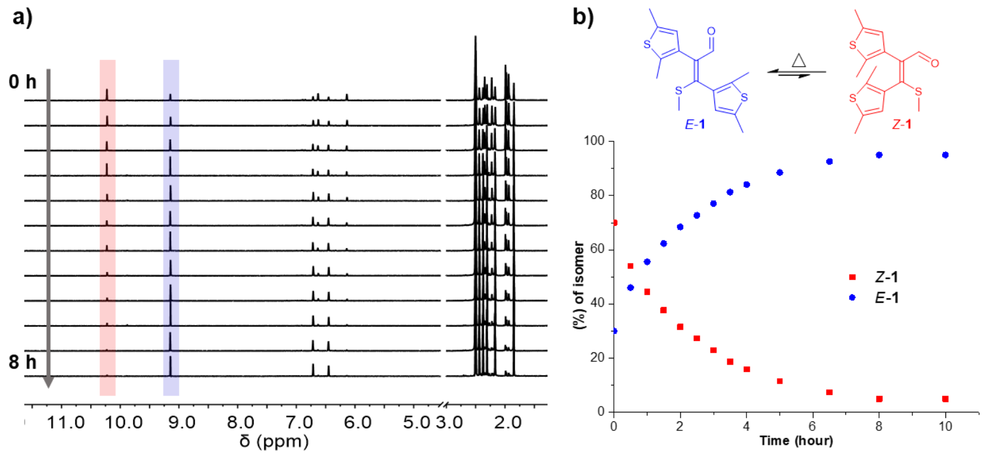

Supplementary Figure 95. Thermal isomerization experiments followed by ^{1}H NMR spectroscopy in regular time intervals. The initial sample (*E*:*Z* = 30:70) was prepared by first illuminating *E*-**1** with UV light (313 nm, 90 min) and then illuminating it with visible light (535 nm, 30 min). (a) Partial ^{1}H NMR spectra (400 MHz, DMSO-d_6, 25 ℃) recorded during the conversion of metastable isomer *Z*-**1** to stable isomer *E*-**1** at 80 ℃. (b) Schematic representation of the thermal isomerization of *Z*-**1** to *E*-**1** and the conversion of isomers of **1** in % at 80 ℃.

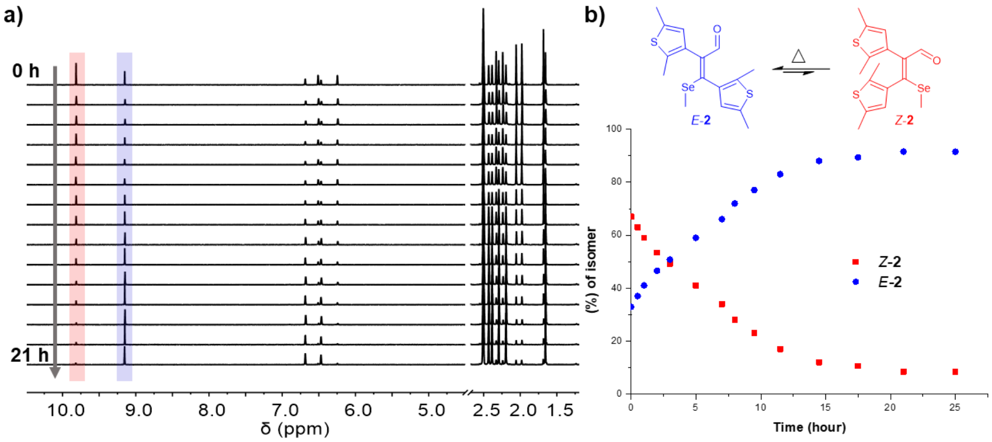

Supplementary Figure 96. Thermal isomerization experiments followed by ^{1}H NMR spectroscopy in regular time intervals. The initial sample (*E*:*Z* = 33:67) was prepared by first illuminating *E*-**2** with UV light (313 nm, 70 min) and then illuminating it with visible light (535 nm, 20 min). (a) Partial ^{1}H NMR spectra (400 MHz, DMSO-d_6, 25 ℃) recorded during the conversion of metastable isomer *Z*-**2** to stable isomer *E*-**2** at 125 ℃. (b) Schematic representation of the thermal isomerization of *Z*-**2** to *E*-**2** and the conversion of isomers of **2** in % at 125 ℃.

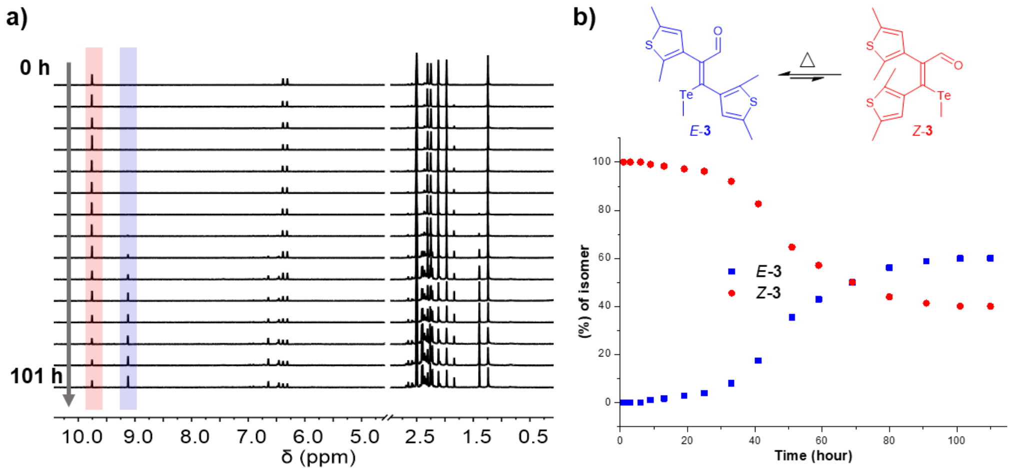

Supplementary Figure 97. Thermal isomerization experiments followed by ^{1}H NMR spectroscopy in regular time intervals. (a) Partial ^{1}H NMR spectra (400 MHz, DMSO-d_6, 25 ℃) recorded during the conversion of isomer *Z*-**3** to isomer *E*-**3** at 125 ℃. (b) Schematic representation of the thermal isomerization of *Z*-**3** to *E*-**3** and the conversion of isomers of **3** in % at 125 ℃.

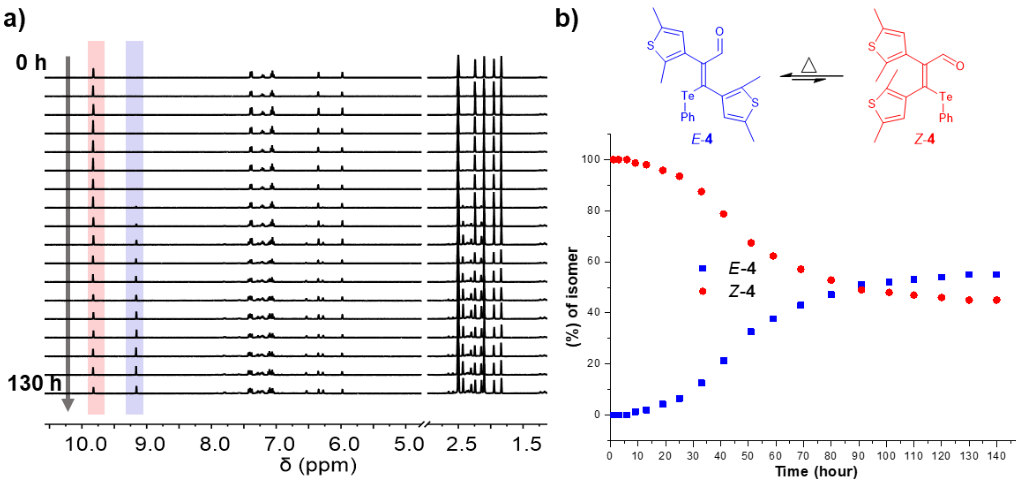

Supplementary Figure 98. Thermal isomerization experiments followed by ^{1}H NMR spectroscopy in regular time intervals. (a) Partial ^{1}H NMR spectra (400 MHz, DMSO-d_6, 25 ℃) recorded during the conversion of isomer *Z*-**4** to isomer *E*-**4** at 125 ℃. (b) Schematic representation of the thermal isomerization of *Z*-**4** to *E*-**4** and the conversion of isomers of **4** in % at 125 ℃.

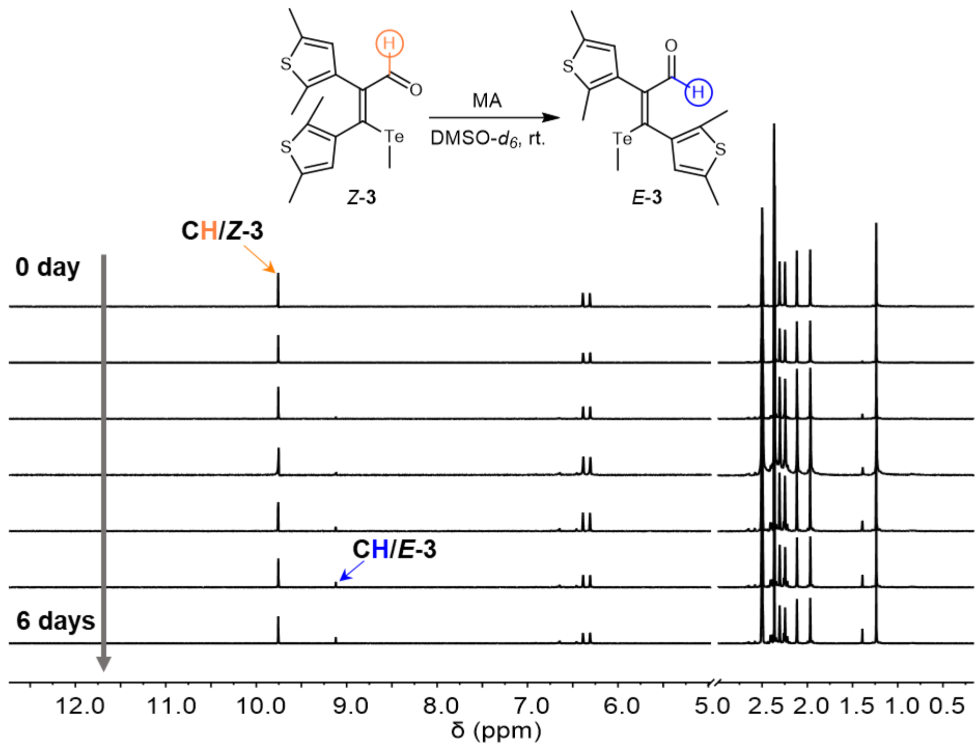

Supplementary Figure 99. ^{1}H NMR spectrum of the reaction of *Z*-**3** (10 mM, 25 ℃) and MA (1.0 equiv.) in DMSO-d_6 at varied time. The ratio of *E*:*Z* is 18:82 after 6 days.

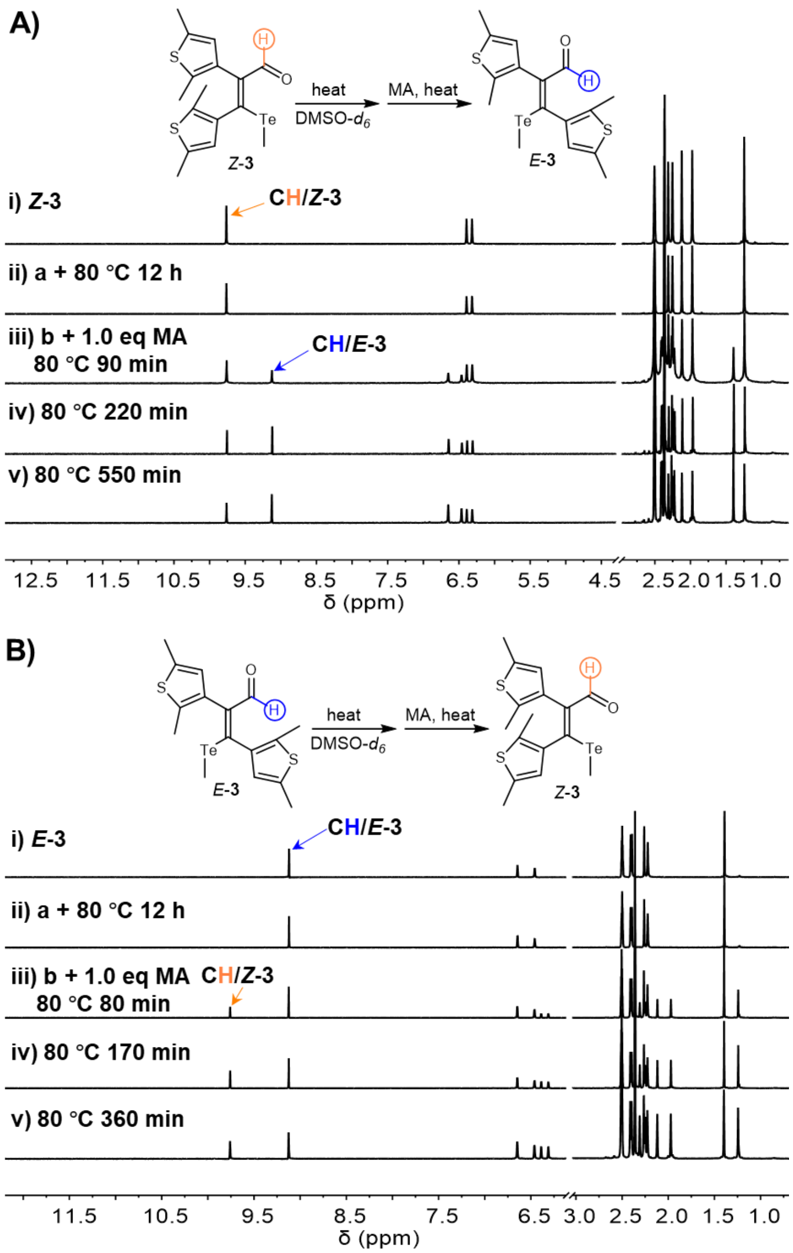

Supplementary Figure 100. (**A**): (Ai) ^{1}H NMR spectrum of *Z*-**3** (10 mM) in DMSO-d_6 at 25 ℃; (Aii) Heating at 80 ℃ for 12 h; (Aiii) Further addition of methanesulfonic acid (MA) (1.0 equiv.) and heating at 80 ℃ for 90 min; (Aiv) Further heating at 80 ℃ for 220 min; (Av) Further heating at 80 ℃ for 550 min. The ratio of *E*:*Z* is 58:42; (**B**): (Bi) ^{1}H NMR spectrum of *E*-**3** (10 mM) in DMSO-d_6; (Bii) Heating at 80 ℃ for 12 h; (Biii) Further addition of methanesulfonic acid (MA) (1.0 equiv.) and heating at 80 ℃ for 80 min; (Biv) Further heating at 80 ℃ for 170 min; (Bv) Further heating at 80 ℃ for 360 min. The ratio of *E*:*Z* is 58:42.

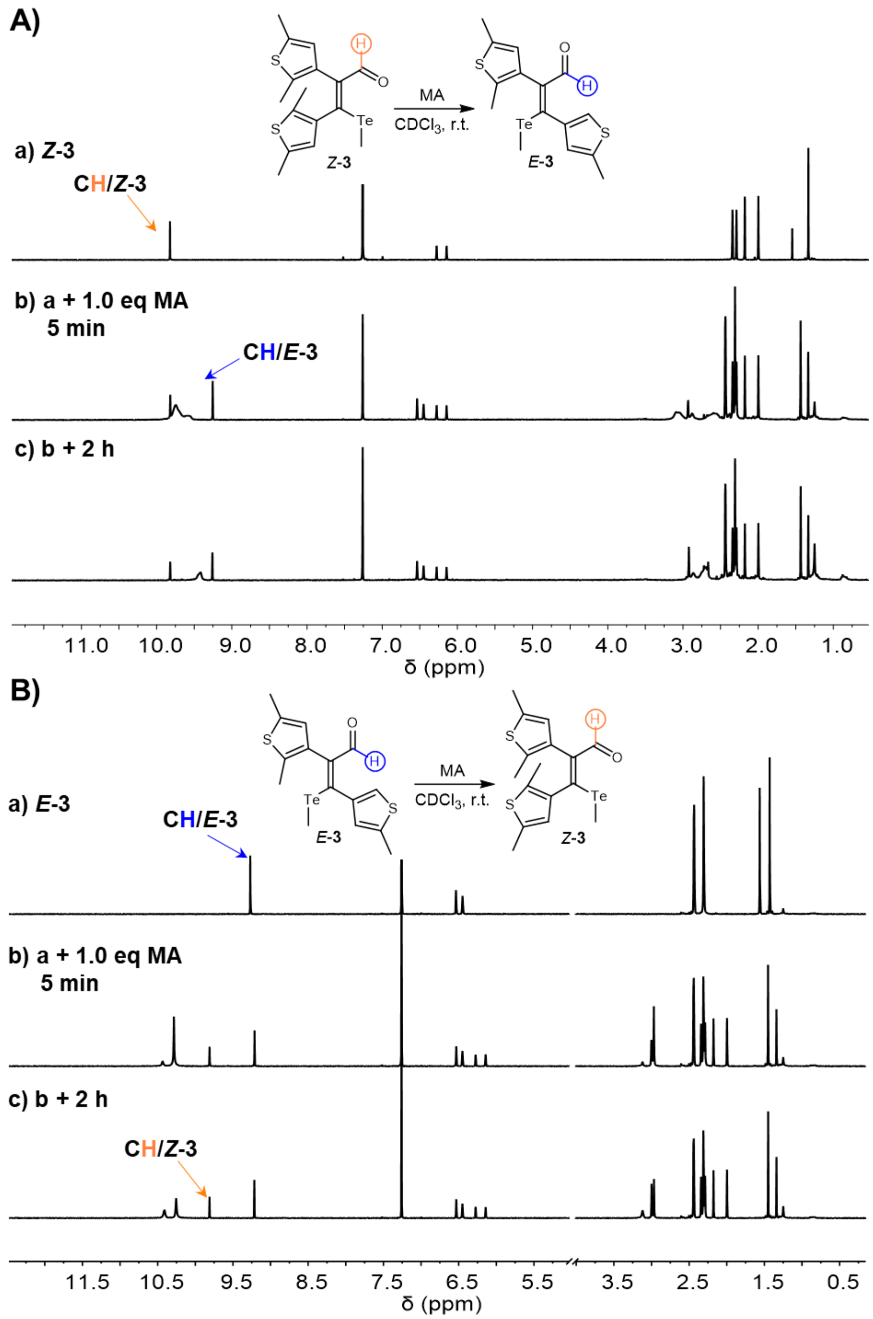

Supplementary Figure 101. (**A**): ^{1}H NMR spectrum of the reaction of *Z*-**3** (10 mM, 25 °C) and MA (1.0 equiv.) in $CDCl_3$ at varied time. The equilibrium was reached after 5 min. The ratio of *E*:*Z* is 60:40; (**B**): ^{1}H NMR spectrum of the reaction of *E*-**3** (10 mM, 25 °C) and MA (1.0 equiv.) in $CDCl_3$ at varied time. The equilibrium was reached after 5 min. The ratio of *E*:*Z* is 60:40.

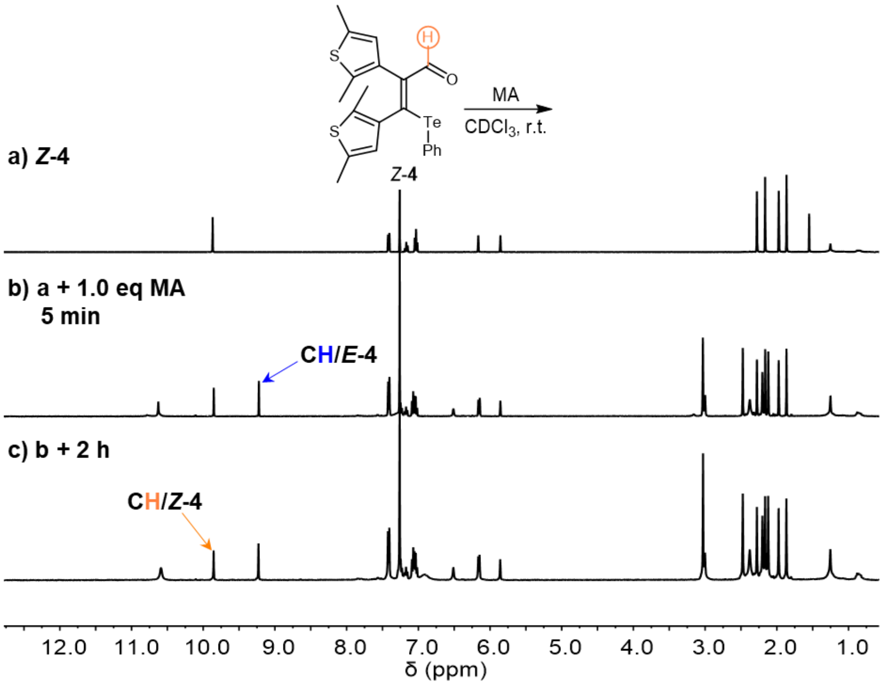

Supplementary Figure 102. ^{1}H NMR spectrum of the reaction of *Z*-**4** (10 mM, 25 ℃) and MA (1.0 equiv.) in $CDCl_3$ at varied time. The equilibrium was reached after 5 min. The ratio of *E*:*Z* is 57:43.

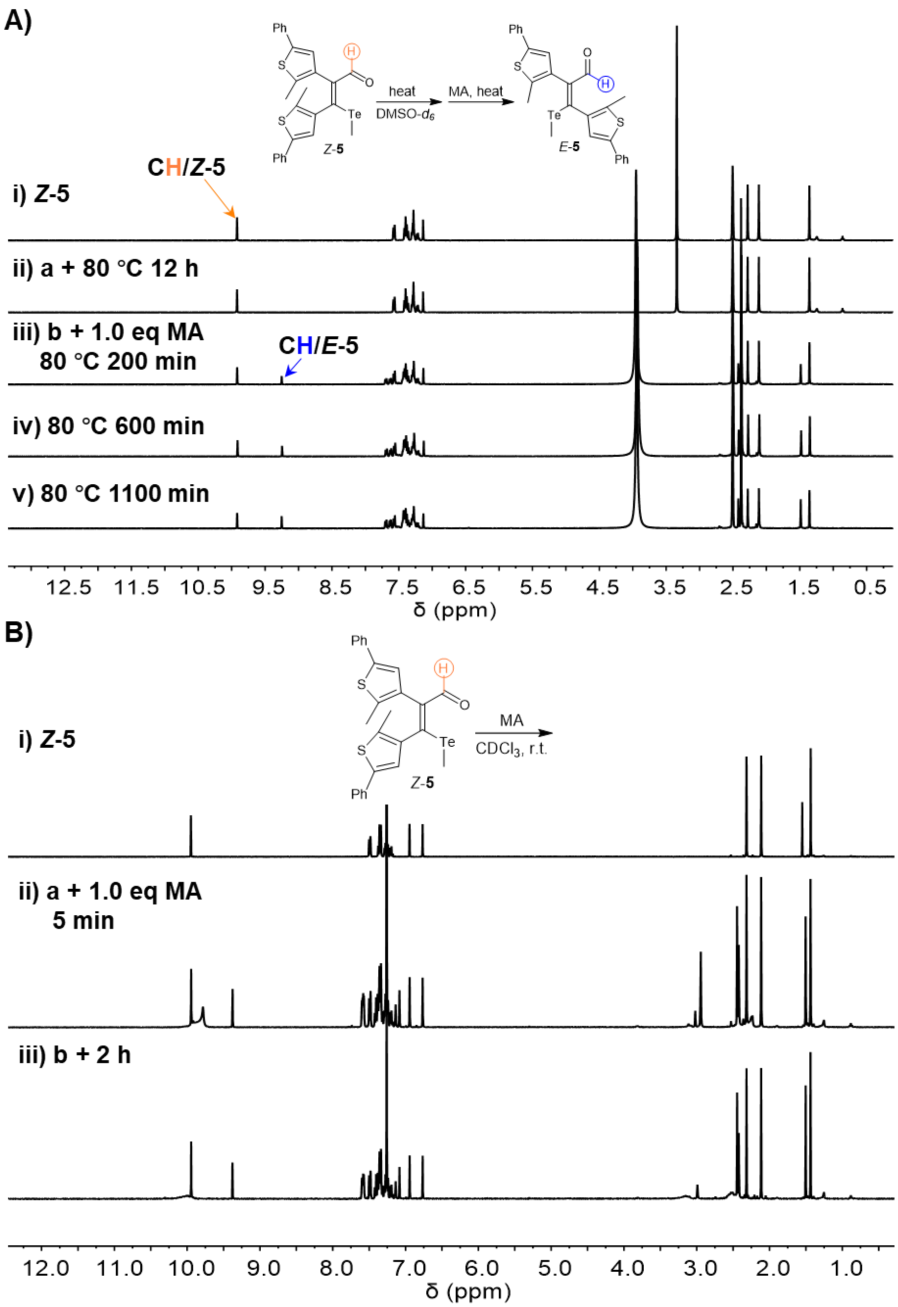

Supplementary Figure 103. (**A**): (Ai) ^{1}H NMR spectrum of *Z*-**5** (10 mM) in DMSO-d_6 at 25 ℃; (Aii) Heating at 80 ℃ for 12 h; (Aiii) Further addition of methanesulfonic acid (MA) (1.0 equiv.) and heating at 80 ℃ for 200 min; (Aiv) Further heating at 80 ℃ for 600 min; (Av) Further heating at 80 ℃ for 1100 min. The ratio of *E*:*Z* is 44:56; (**B**): ^{1}H NMR spectrum of the reaction of *Z*-**5** (10 mM, 25 ℃) and MA (1.0 equiv.) in $CDCl_3$ at varied time. The equilibrium was reached after 5 min. The ratio of *E*:*Z* is 45:55.

Supplementary Note 5. Photoswitching Studies of Cationic Telluronium

Supplementary Table 8. Summary of the distribution at the photostationary state of chalcogen-containing photoswitches for *E/Z/C* isomers of **3**, **5**, **6**, and **7**.

Compound	Ch X	Solvent	λ_{light} (nm)	*E*[a] (%)	*Z*[a] (%)	*C*[a] (%)
E-**3**	TeMe Me	DMSO-d_6	365	4	96	/[c]
			450[b]	45	55	
		$CDCl_3$	365	4	96	/[c]
			450[b]	42	58	
E-**5**	TeMe Ph	DMSO-d_6	365	3	96	1
			450[b]	39	60	1
		$CDCl_3$	365	3	96	1
			450[b]	40	59	1
Z-**6**	$TeMe_2$ Me	DMSO-d_6	425	31	27	42
			535[b]	31	69	0
		$CDCl_3$	425	51	8	41
			535[b]	51	49	0
Z-**7**:*E*-**7**[d] (86:14)	$TeMe_2$ Ph	DMSO-d_6	425	7	12	81
			650[b]	7	93	0

[a] After irradiation with different light determined by NMR. [b] After light illumination of compounds in the first column reached the PSS, the target light source was used for irradiation until reaching the PSS. [c] Not observed. [d] Precipitation in chloroform.

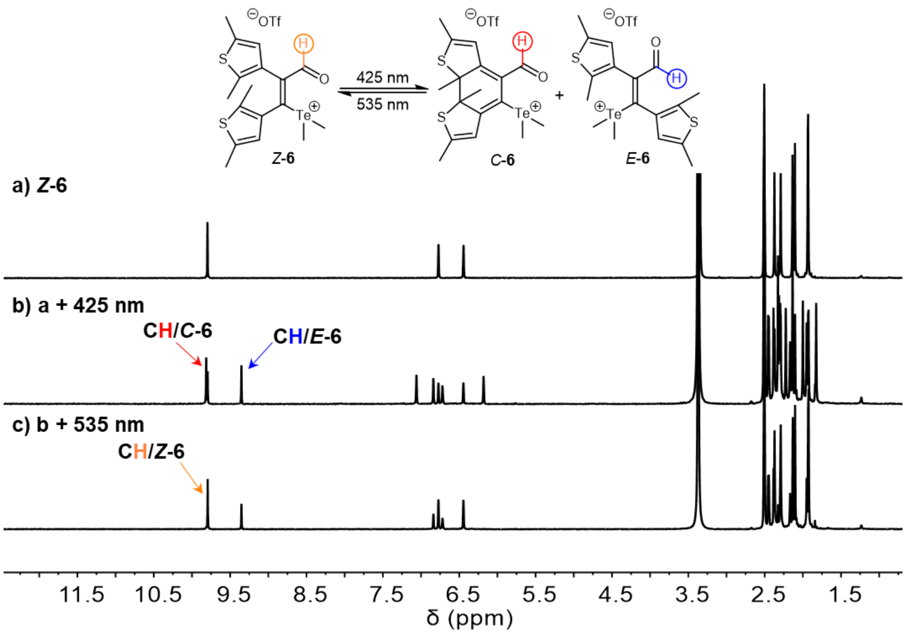

Supplementary Figure 104. (a) ^{1}H NMR spectrum of *Z*-**6** (10 mM) in DMSO-d_6 at 25 °C; (b) Irradiation of *Z*-**6** with visible light (425 nm, 10 min). The ratio of *E*:*Z*:*C* is 31:27:42 (*C*-**6** is stable for three days); (c) Further irradiation with visible light (535 nm, 20 min). The ratio of *E*:*Z* is 31:69.

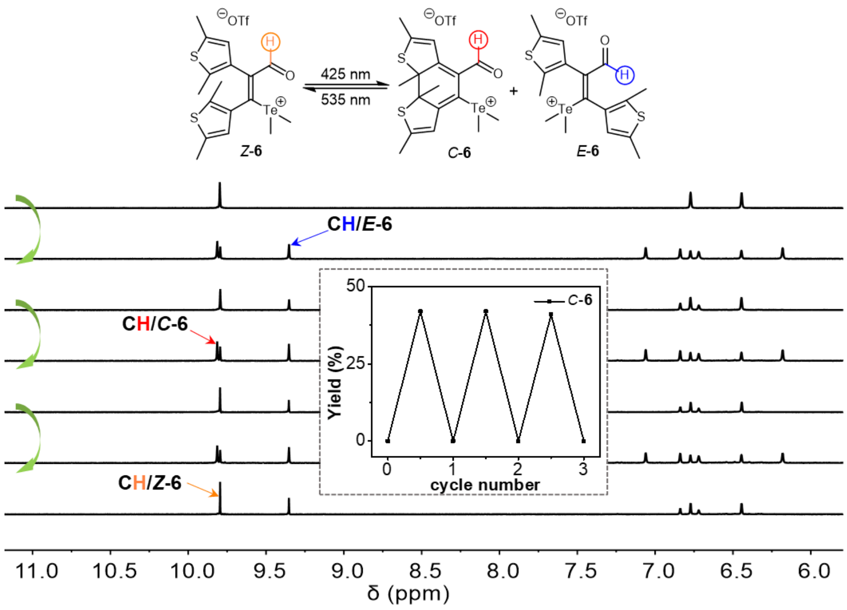

Supplementary Figure 105. ^{1}H NMR spectra of *Z*-**6** (10 mM) in DMSO-d_6 at 25 °C during repetitive photoswitching cycles consisting of alternating visible light (425 nm, 10 min) and visible light (535 nm, 20 min).

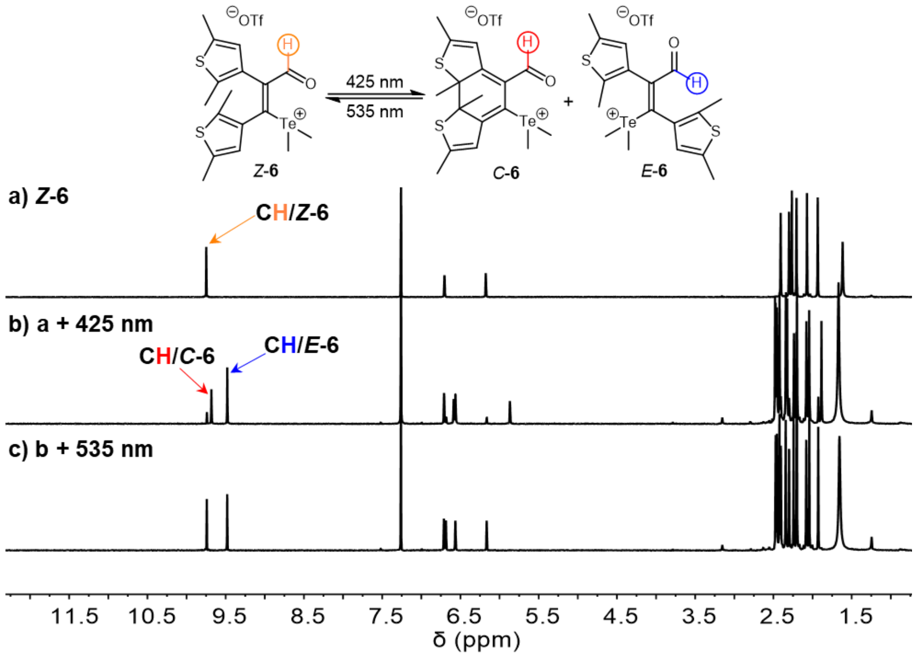

Supplementary Figure 106. (a) ^{1}H NMR spectrum of *Z*-**6** (10 mM) in $CDCl_3$ at 25 °C; (b) Irradiation of *Z*-**6** with visible light (425 nm, 5 min). The ratio of *E*:*Z*:*C* is 51:8:41; (c) Further irradiation with visible light (535 nm, 20 min). The ratio of *E*:*Z* is 51:49.

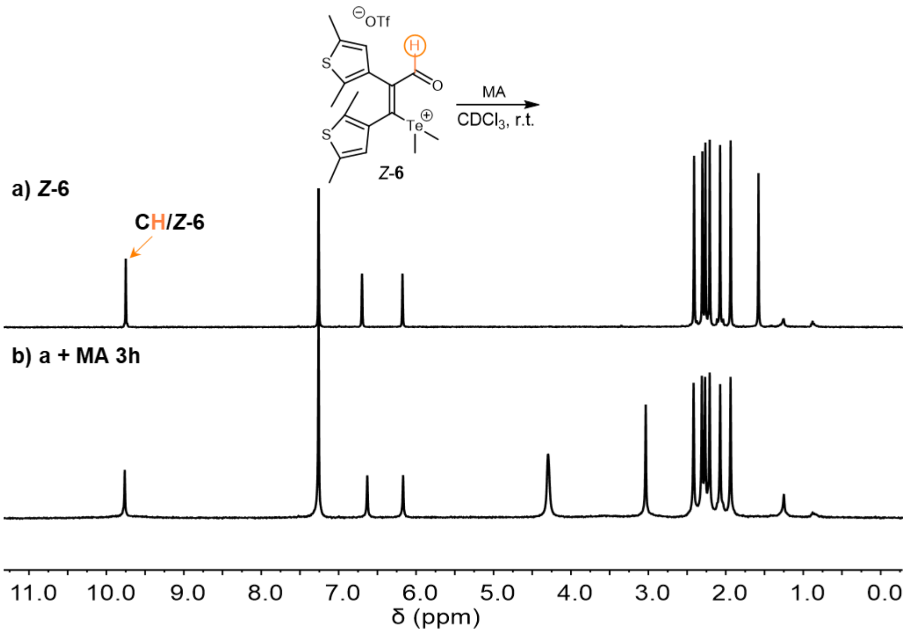

Supplementary Figure 107. (a) ^{1}H NMR spectrum of *Z*-**6** (10 mM) in $CDCl_3$ at 25 °C; (b) The addition of MA (1.0 equiv.) at 25 ℃ for 3 h.

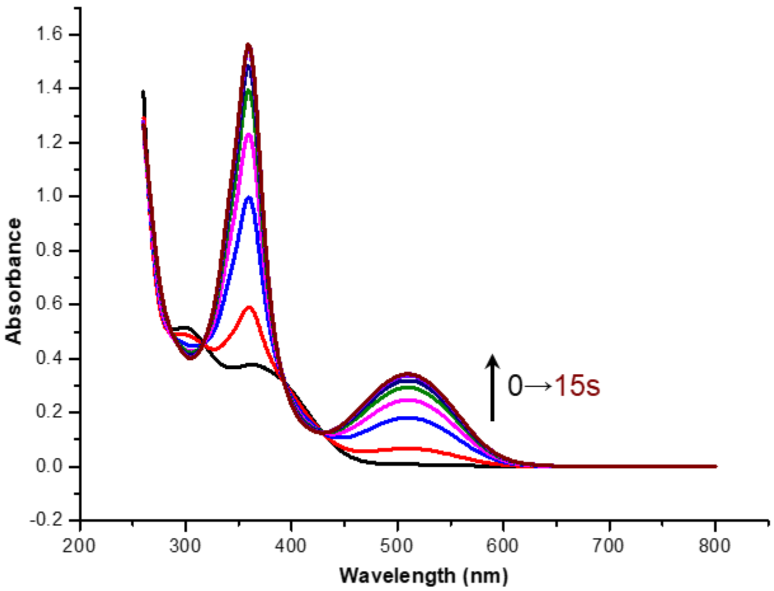

Supplementary Figure 108. UV-vis absorption spectra of *Z*-**6** (100 µM in DMSO) in the pristine state and upon irradiation with 425 nm light after total irradiation of 0, 1, 3, 5, 7, 9, 12 and 15 s. The photostationary state was reached after 15 s.

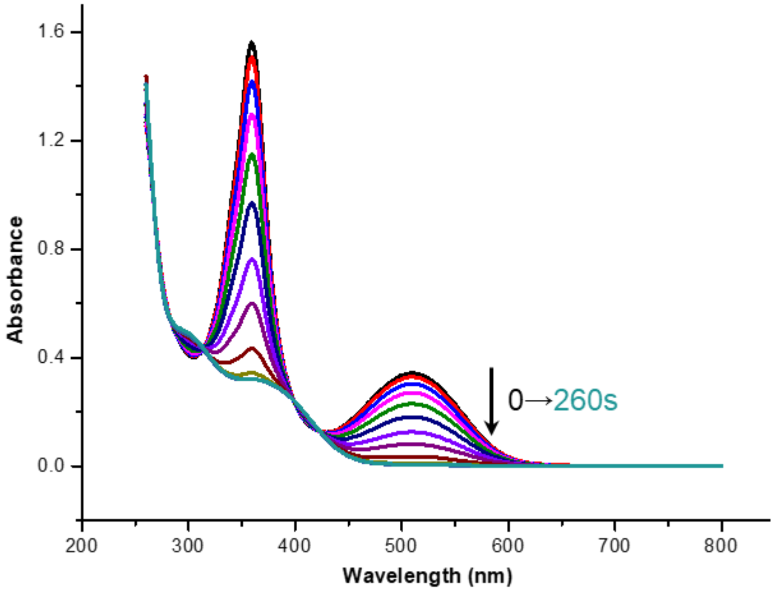

Supplementary Figure 109. UV-vis absorption spectra of *Z*-**6** (100 µM in DMSO) at the PSS (425 nm) and upon irradiation with 535 nm light after total irradiation of 0, 5, 15, 30, 50, 75, 105, 140, 180, 220, 260 s. The photostationary state was reached after 260 s.

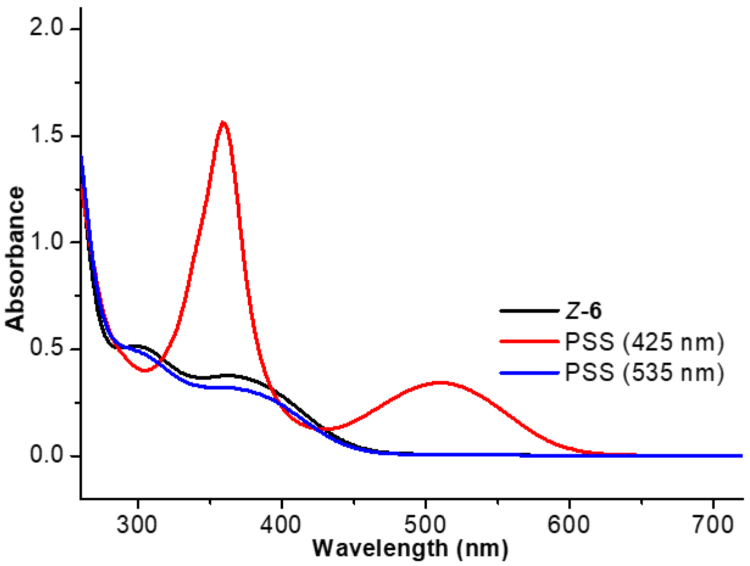

Supplementary Figure 110. UV-vis absorption spectra of *Z*-**6** (100 μM in DMSO) in the pristine state and at the PSS upon irradiation with different wavelength of light (425 then 535 nm).

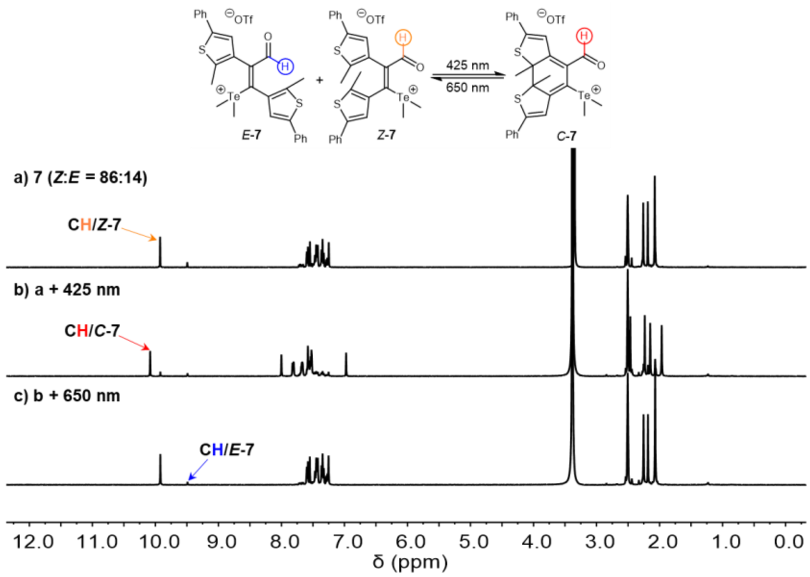

Supplementary Figure 111. (a) ^{1}H NMR spectrum of **7** (*Z*:*E* = 86:14, 10 mM) in DMSO-d_6 at 25 ℃; (b) Irradiation of **7** with visible light (425 nm, 70 min). The ratio of *E*:*Z*:*C* is 7:12:81(*C*-**7** is stable for seven days); (c) Further irradiation with visible light (650 nm, 180 min). The ratio of *E*:*Z* is 7:93.

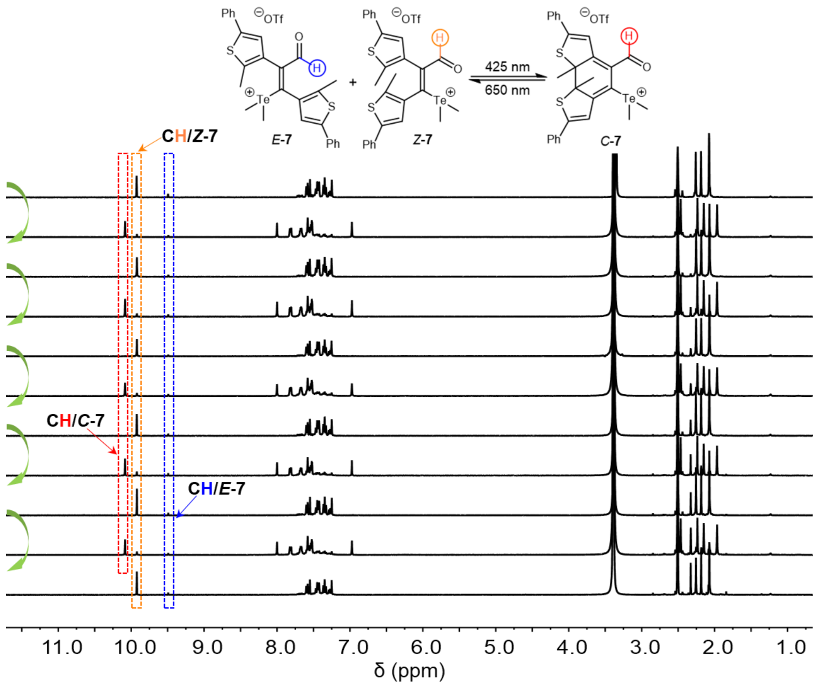

Supplementary Figure 112. ^{1}H NMR spectra of **7** (Z:E = 86:14, 10 mM) in DMSO-d_6 at 25 °C during repetitive photoswitching cycles consisting of alternating visible light (425 nm, 70 min) and visible (650 nm, 180 min) light.

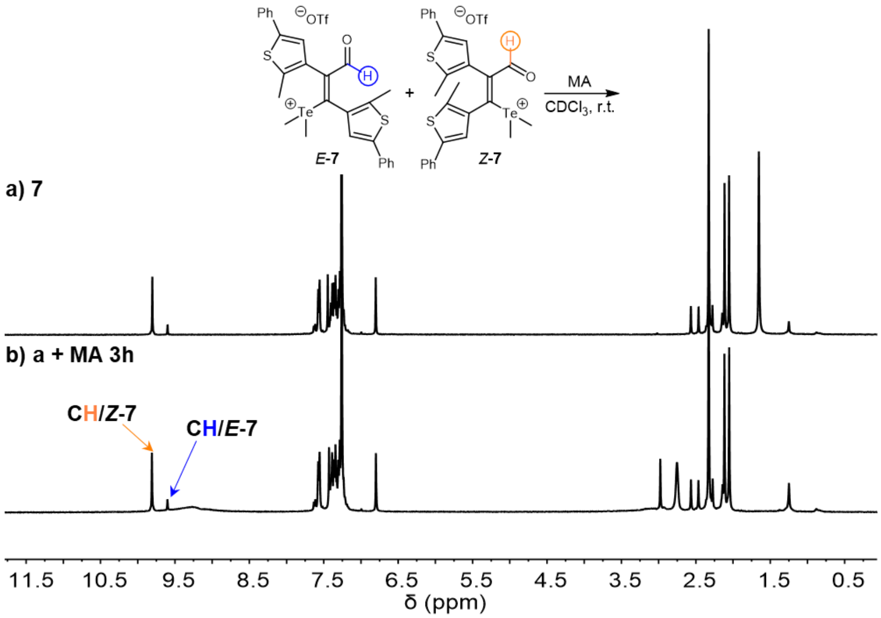

Supplementary Figure 113. (a) ^{1}H NMR spectrum of **7** (Z:E = 86:14, 10 mM) in $CDCl_3$ at 25 °C; (b) The addition of MA (1.0 equiv.) at 25 ℃ for 3 h.

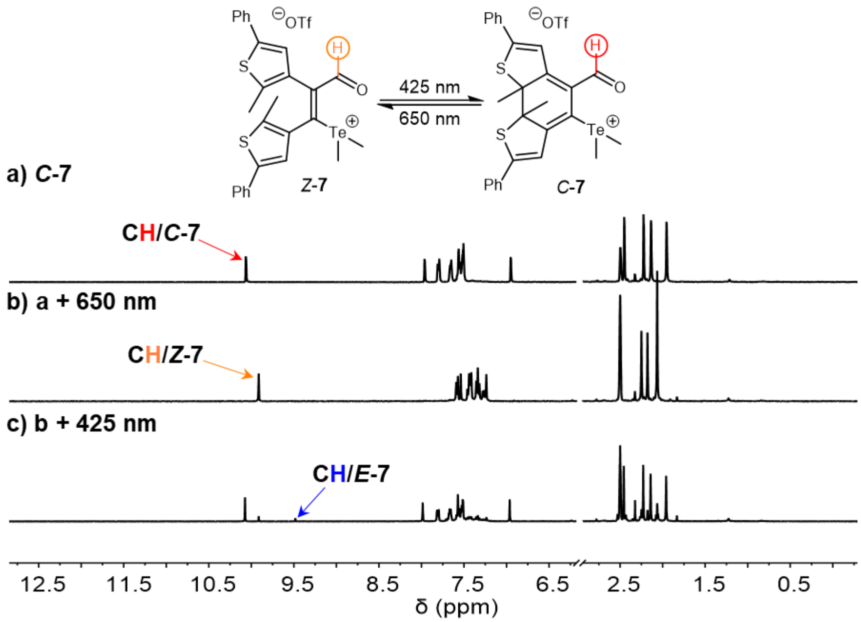

Supplementary Figure 114. (a) ^{1}H NMR spectrum of *C*-**7** in DMSO-d_6 at 25 °C; (b) Irradiation of *C*-**7** with visible light (650 nm, 200 min). The ratio of *E*:*Z*:*C* is 0:100:0; (c) Further irradiation with visible light (425 nm, 70 min). The ratio of *E*:*Z*:*C* is 7:12:81.

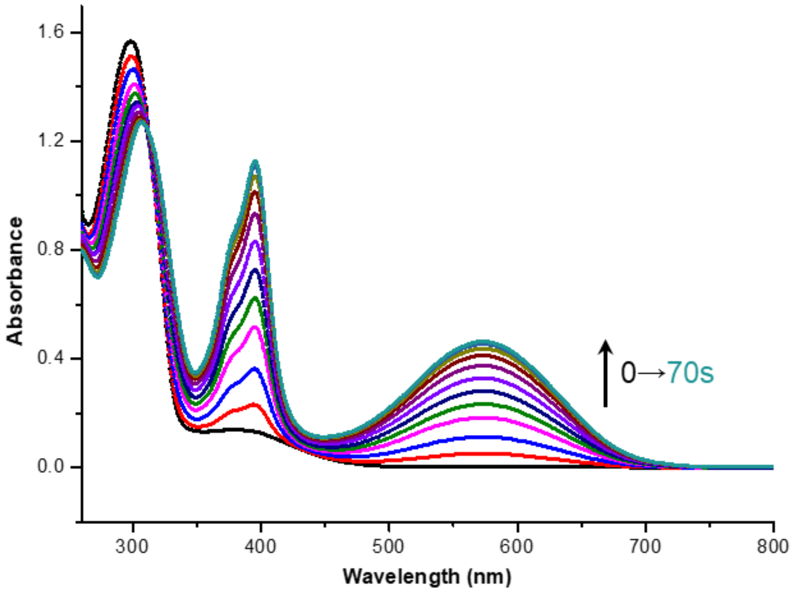

Supplementary Figure 115. UV-vis absorption spectra of **7** (50 μM in DMSO) in the pristine state and upon irradiation with 425 nm light after total irradiation of 0, 1, 3, 6, 9, 13, 18, 25, 34, 45, 58, and 70 s. The photostationary state was reached after 70 s.

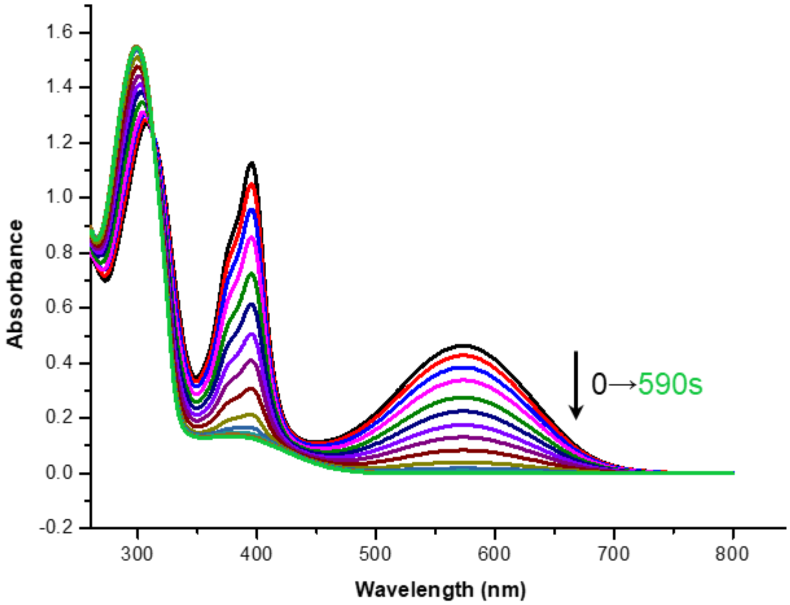

Supplementary Figure 116. UV-vis absorption spectra of **7** (50 µM in DMSO) at the PSS (425 nm) and upon irradiation with 650 nm light after total irradiation of 0, 10, 25, 45, 70, 100, 135, 170, 230, 300, 370, 440, 510, and 590 s. The photostationary state was reached after 590 s.

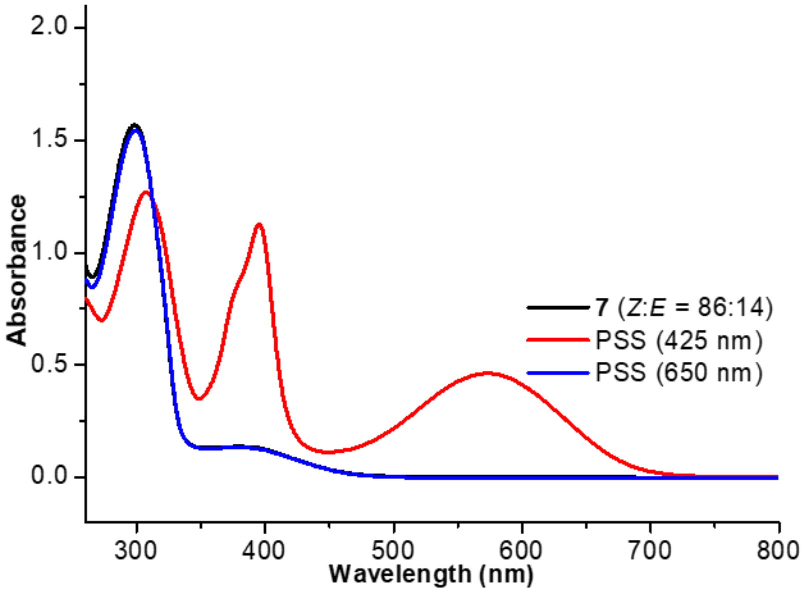

Supplementary Figure 117. UV-vis absorption spectra of **7** (50 µM in DMSO) in the pristine state and at the PSS upon irradiation with different wavelength of light (425 then 650 nm).

Supplementary Note 6. Dynamic Covalent Chemistry Gated Photoswitching

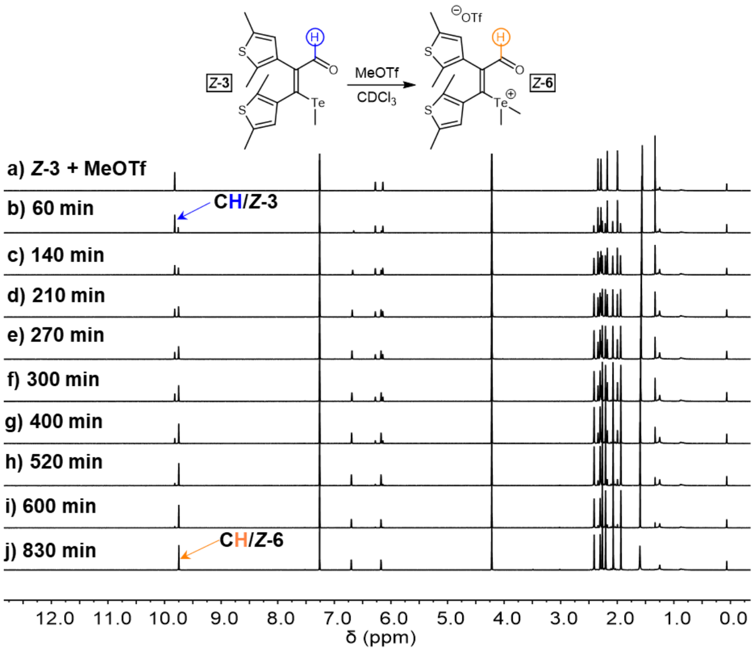

Supplementary Figure 118. ^{1}H NMR spectra of the reaction of *Z*-**3** (10 mM) and MeOTf (1.8 equiv.) in $CDCl_3$ at varied time. The yield of *Z*-**6** is 100% after reaching equilibrium at 25 °C.

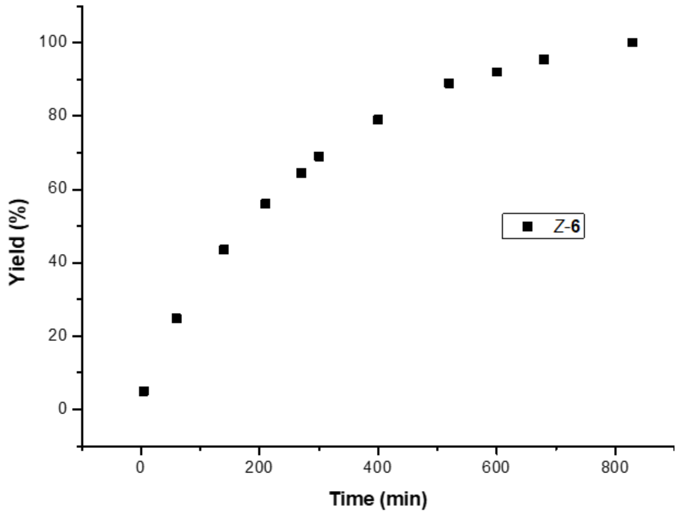

Supplementary Figure 119. Kinetic profile of the reaction of *Z*-**3** (10 mM) and MeOTf (1.8 equiv.) in $CDCl_3$ at 25 °C.

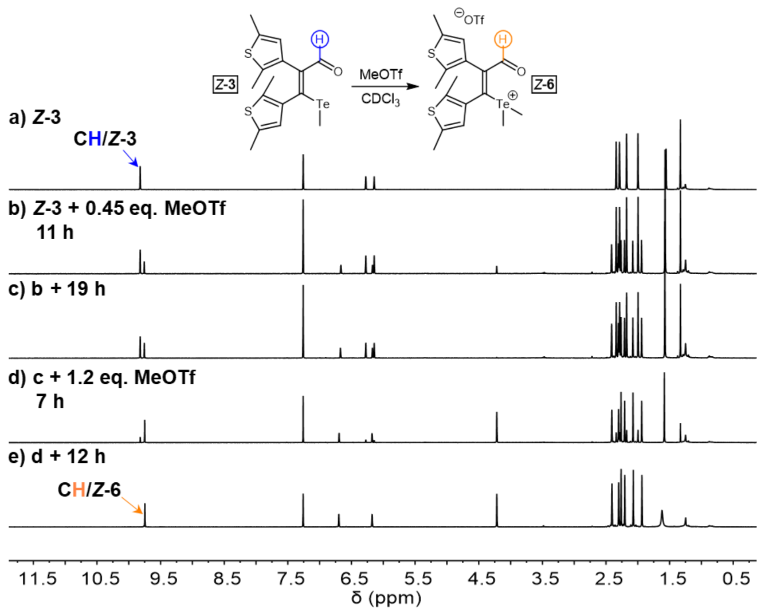

Supplementary Figure 120. (a) ^{1}H NMR spectrum of *Z*-**3** (10 mM) in $CDCl_3$ at 25 °C; (b) Addition of MeOTf (0.45 equiv.) and waiting for 11 h. The yield of *Z*-**6** is 31%; (c) Further waiting for 19 h. The yield of *Z*-**6** is 45%; (d) Further addition of MeOTf (1.2 equiv.) and waiting for 7 h. The yield of *Z*-**6** is 79%; (e) Further waiting for 12 h. The yield of *Z*-**6** is 100%.

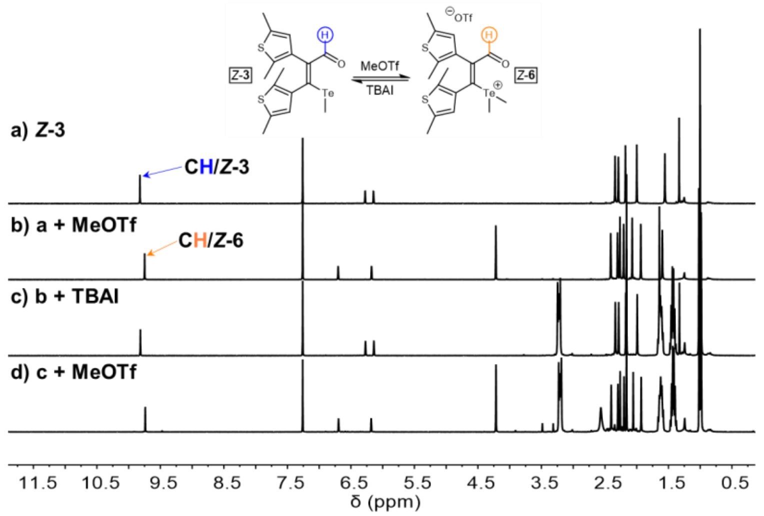

Supplementary Figure 121. (a) ^{1}H NMR spectrum of *Z*-**3** (10 mM) in $CDCl_3$ at 25 °C; (b) The addition of MeOTf (1.8 equiv.). The yield of *Z*-**6** is 100%; (c) Further addition of TBAI (2.0 equiv.). The yield of *Z*-**3** is 100%. (d) Further addition of MeOTf (2.2 equiv.). The yield of *Z*-**6** is 100%.

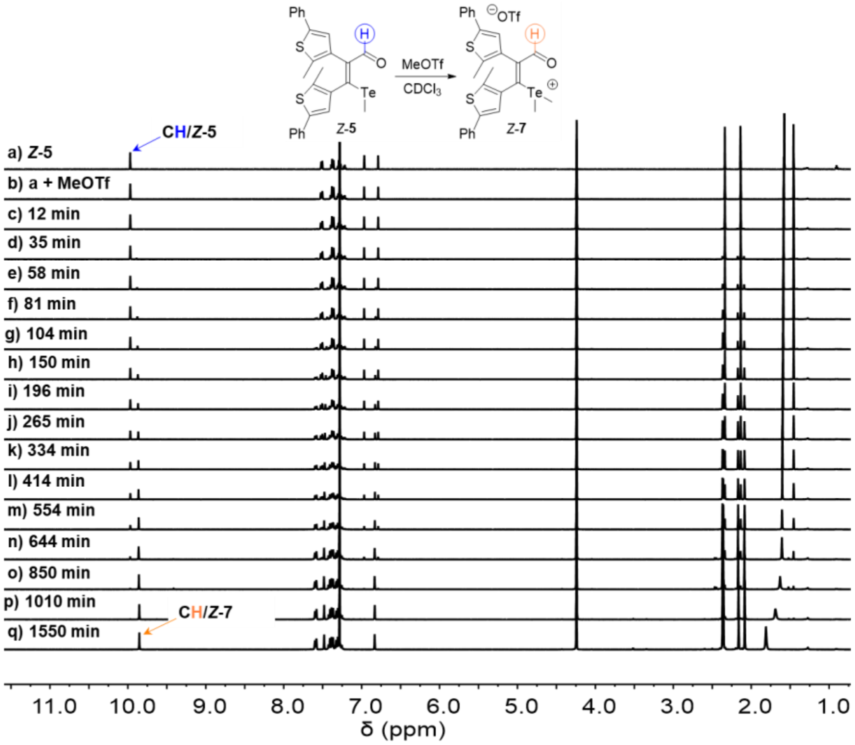

Supplementary Figure 122. ^{1}H NMR spectra of the reaction of *Z*-**5** (10 mM) and MeOTf (3.0 equiv.) in $CDCl_3$ at varied time. The yield of *Z*-**7** is 100% after reaching equilibrium.

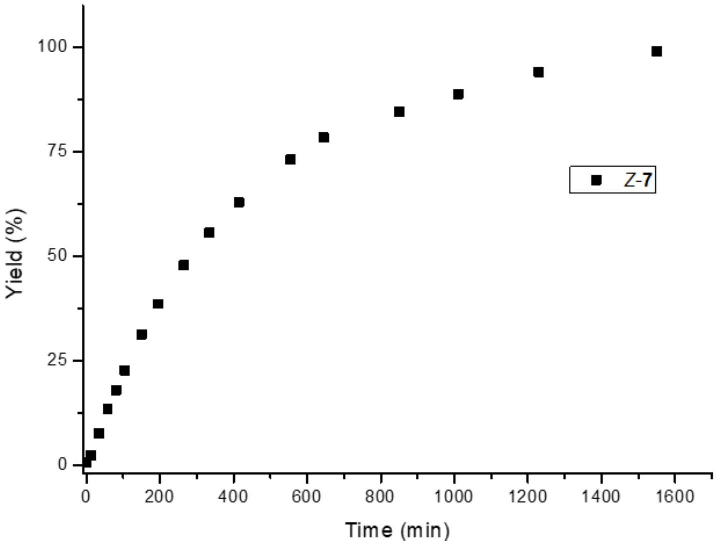

Supplementary Figure 123. Kinetic profile of the reaction of *Z*-**5** (10 mM) and MeOTf (3.0 equiv.) in $CDCl_3$.

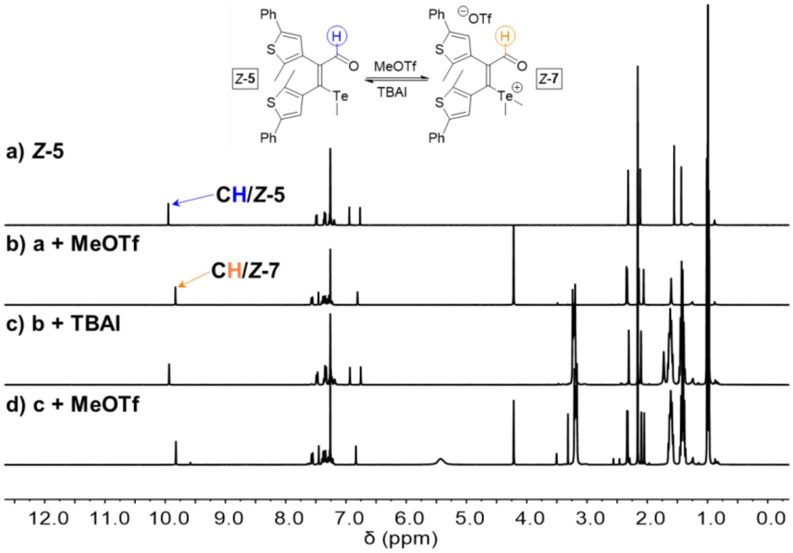

Supplementary Figure 124. (a) ^{1}H NMR spectrum of *Z*-**5** (10 mM) in $CDCl_3$ at 25 °C; (b) The addition of MeOTf (3.0 equiv.). The yield of *Z*-**7** is 100%; (c) Further addition of TBAI (3.2 equiv.). The yield of *Z*-**5** is 100%; (d) Further addition of MeOTf (3.2 equiv.). The yield of **5** (*Z*:*E* = 91:9) is 100%

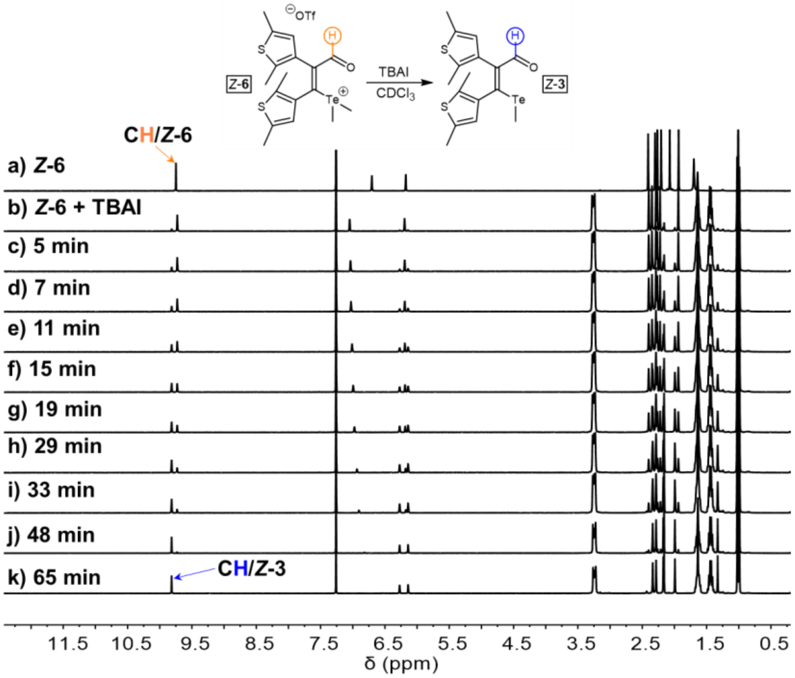

Supplementary Figure 125. ^{1}H NMR spectra of the reaction of *Z*-**6** (10 mM) and tetrabutylammonium iodide (TBAI) (1.3 equiv.) in $CDCl_3$ at varied time. The yield of *Z*-**3** is 100% after 65 min at 25 °C.

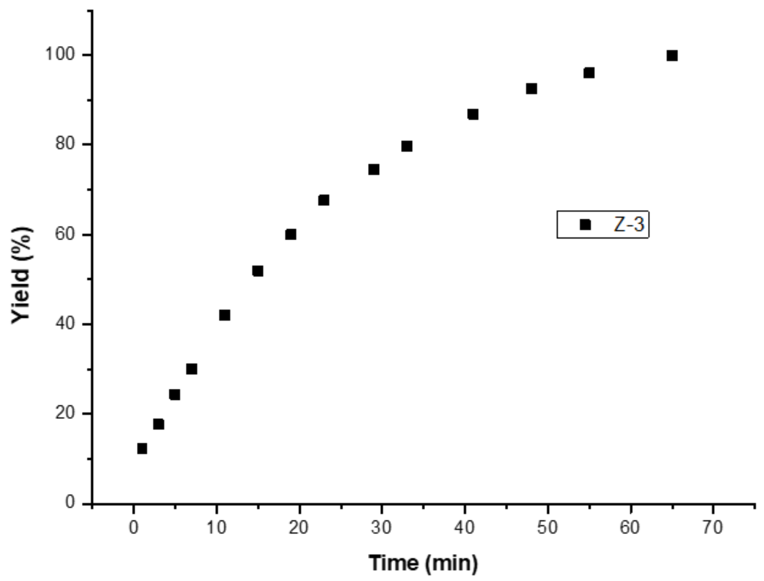

Supplementary Figure 126. Kinetic profile of the reaction of *Z*-**6** (10 mM) and TBAI (1.3 equiv.) in $CDCl_3$ at 25 °C.

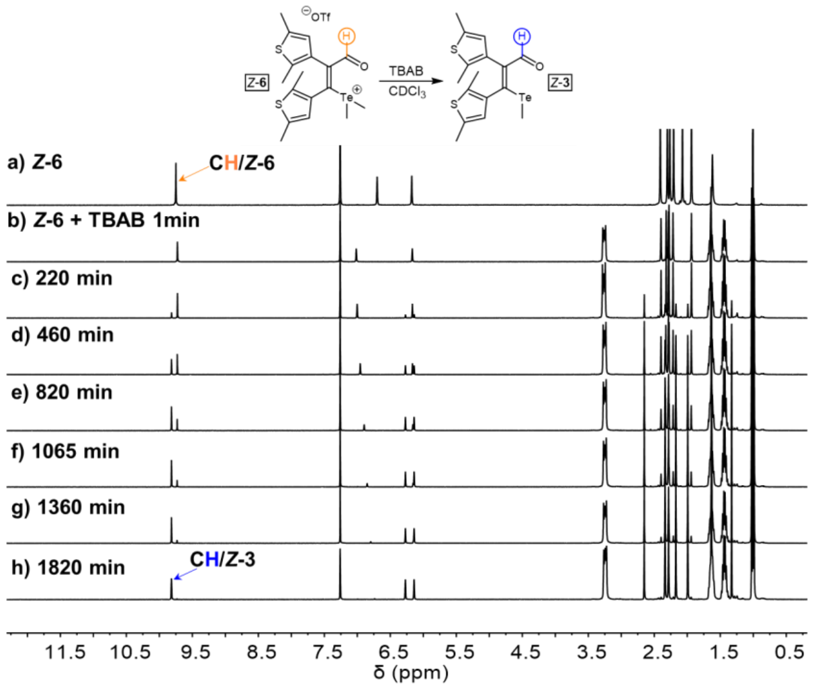

Supplementary Figure 127. ^{1}H NMR spectra of the reaction of *Z*-**6** (10 mM) and tetrabutylammonium bromide (TBAB) (1.3 equiv.) in $CDCl_3$ at varied time. The yield of *Z*-**3** is 100% after 30 h at 25 °C.

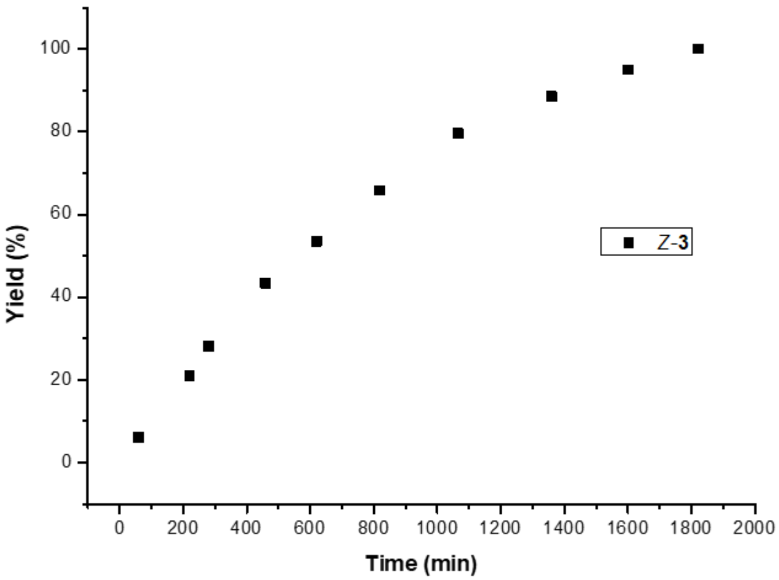

Supplementary Figure 128. Kinetic profile of the reaction of *Z*-**6** (10 mM) and TBAB (1.3 equiv.) in $CDCl_3$ at 25 ℃.

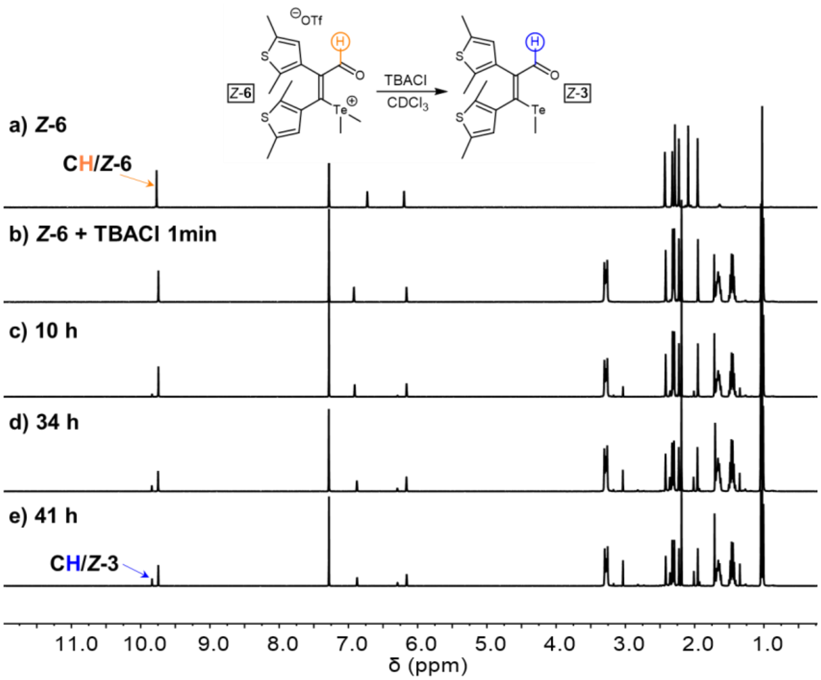

Supplementary Figure 129. ^{1}H NMR spectra of the reaction of *Z*-**6** (10 mM) and tetrabutylammonium chloride (TBACl) (1.3 equiv.) in $CDCl_3$ at varied time. The yield of *Z*-**3** was 43% at 41 h.

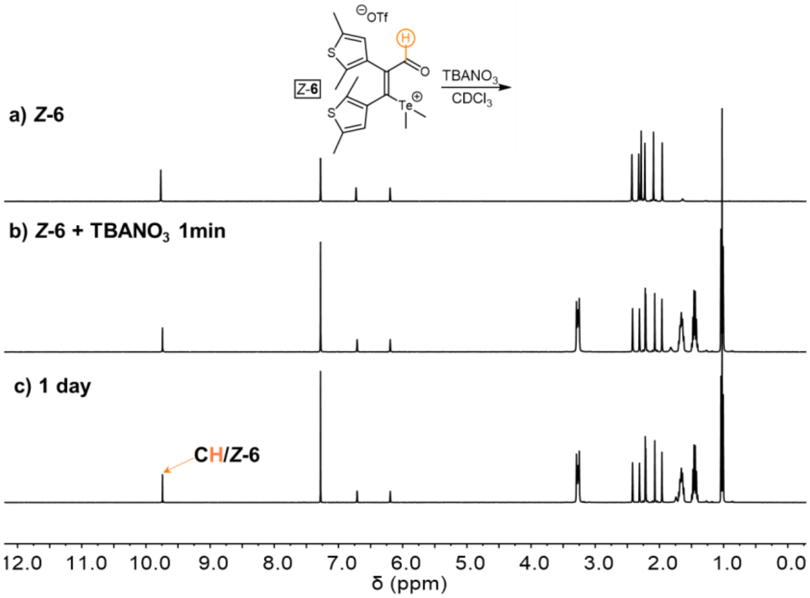

Supplementary Figure 130. ^{1}H NMR spectra of the reaction of *Z*-**6** (10 mM) and tetrabutylammonium nitrate (TBANO$_3$) (1.3 equiv.) in CDCl$_3$ at varied time. *Z*-**3** was not observed.

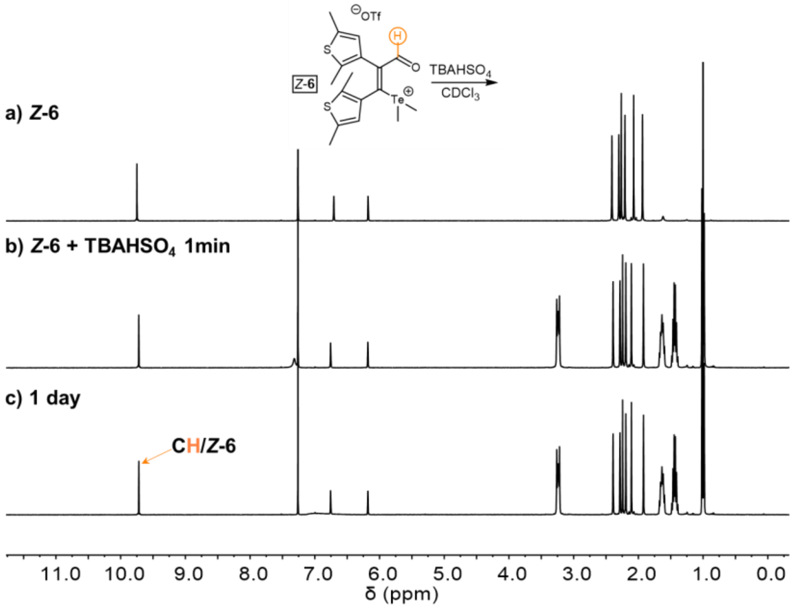

Supplementary Figure 131. ^{1}H NMR spectra of the reaction of *Z*-**6** (10 mM) and tetrabutylammonium hydrogen sulfate (TBAHSO$_4$) (1.3 equiv.) in CDCl$_3$ at varied time. *Z*-**3** was not observed.

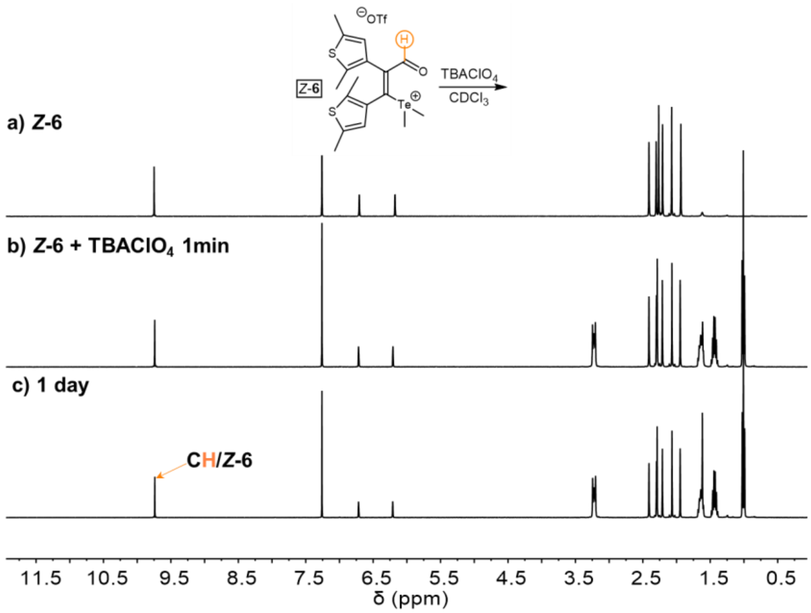

Supplementary Figure 132. ^{1}H NMR spectra of the reaction of *Z*-**6** (10 mM) and tetrabutylammonium perchlorate (TBAClO$_4$) (1.3 equiv.) in CDCl$_3$ at varied time. *Z*-**3** was not observed.

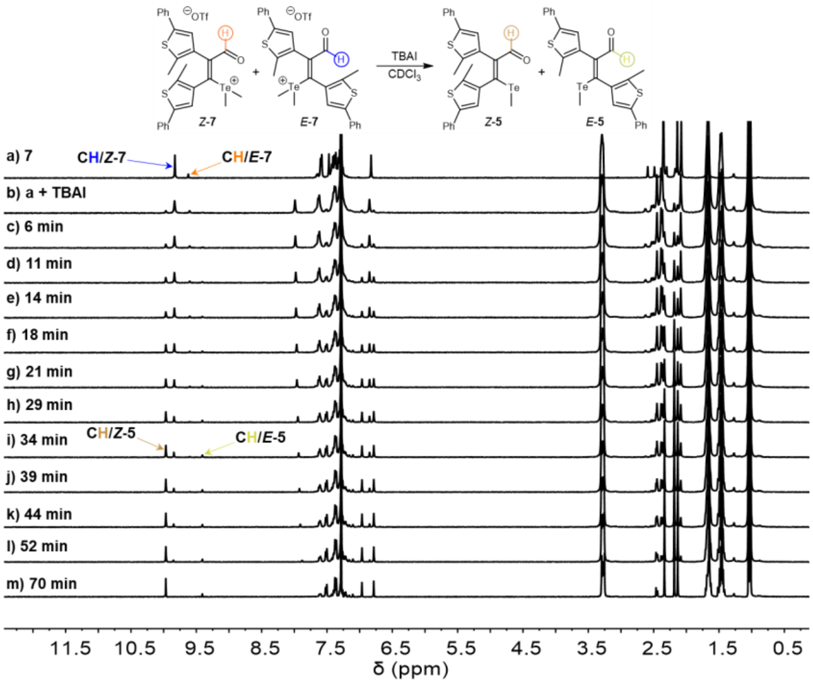

Supplementary Figure 133. ^{1}H NMR spectra of the reaction of **7** (*Z*:*E* = 86:14, 10 mM) and tetrabutylammonium iodide (TBAI) (1.3 equiv.) in CDCl$_3$ at varied time. The yield of **5** (*Z*:*E* = 86:14) is 100% after 70 min.

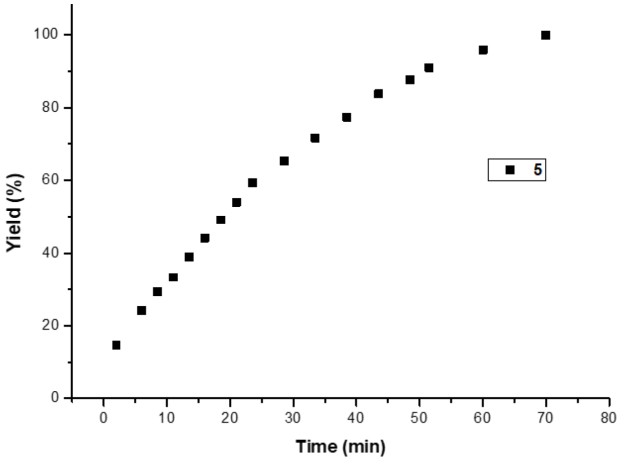

Supplementary Figure 134. Kinetic profile of the reaction of **7** (Z:E = 86:14, 10 mM) and TBAI (1.3 equiv.) in $CDCl_3$.

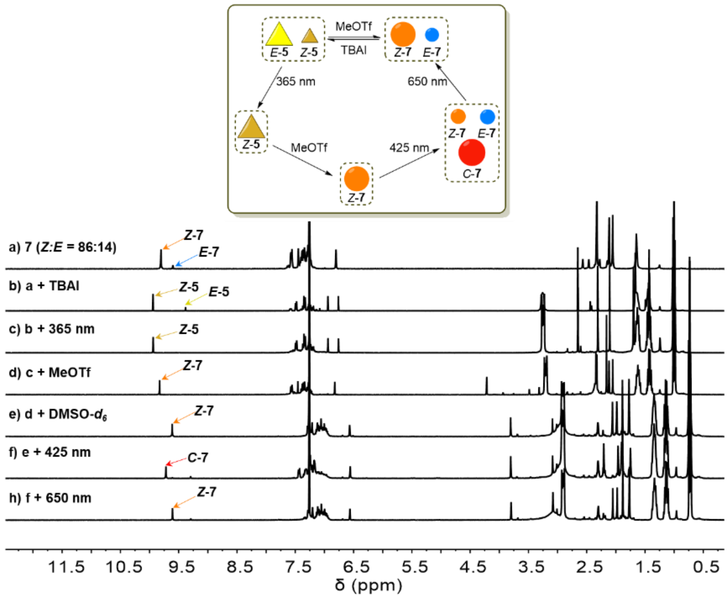

Supplementary Figure 135. The control of the distribution of photochromic states via dynamic covalent reactions within one multistep cycle (the formyl peaks of different compounds are labeled). (a) ^{1}H NMR spectrum of **7** (Z:E = 86:14, 10 mM) in $CDCl_3$ (600 μl) at 25 °C; (b) The addition of TBAI (1.3 equiv.). The yield of **5** (Z:E = 86:14) is 100%; (c) Further irradiation with UV light (365 nm, 10 min). The ratio of E:Z is 98:2; (d) Further addition of MeOTf (2.3 equiv.). The yield of **7** is 100%; (e) Further addition of DMSO-d_6 (80 μl); (f) Further irradiation with visible light (425 nm, 40 min). The ratio of E:Z:C is 11:5:84; (h) Further irradiation with visible light (650 nm, 180 min). The ratio of E:Z is 11:89. The movement of NMR peaks in e-h is due to the addition of DMSO-d_6 to $CDCl_3$.

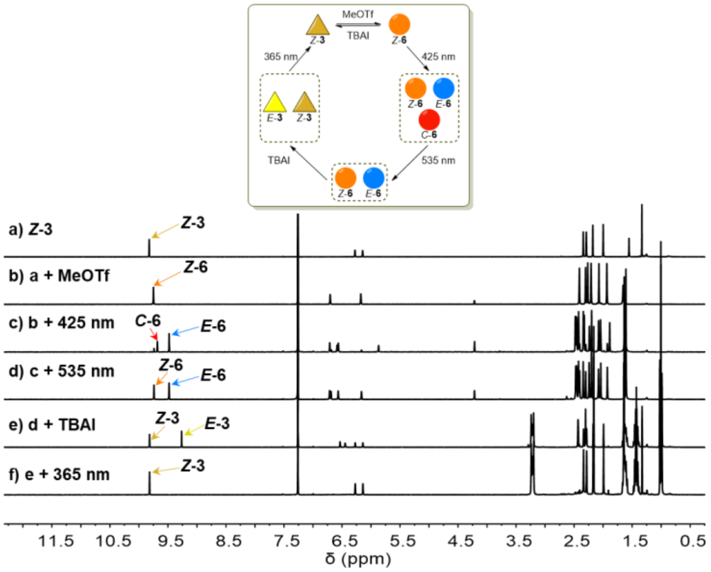

Supplementary Figure 136. The control of the distribution of photochromic states via dynamic covalent reactions within one multistep cycle (the formyl peaks of different compounds are labeled). (a) ^{1}H NMR spectrum of *Z*-**3** (10 mM) in $CDCl_3$ at 25 °C; (b) The addition of MeOTf (1.1 equiv.). The yield of *Z*-**6** is 100%; (c) Further irradiation with visible light (425 nm, 5 min). The ratio of *E*:*Z*:*C* is 52:9:39; (d) Further irradiation with visible light (535 nm, 20 min). The ratio of *E*:*Z* is 52:48; (e) Further addition of TBAI (1.3 equiv.). The ratio of *E*-**3**:*Z*-**3** is 52:48; (f) Further irradiation with UV light (365 nm, 25 min). The ratio of *E*:*Z* is 3:97.

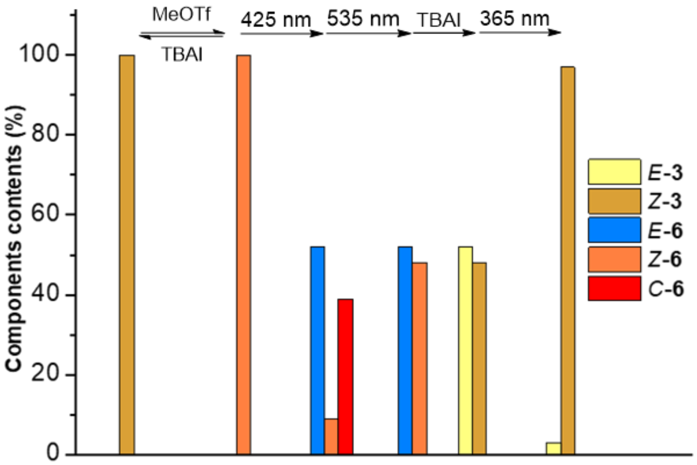

Supplementary Figure 137. The control of the distribution of photochromic states via dynamic covalent reactions within one multistep cycle (5% experimental error).

Supplementary Note 7. Photoaddressed Rewritable Materials

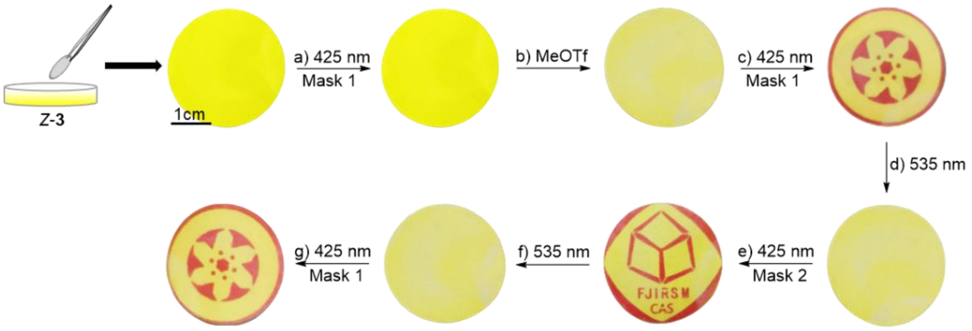

Supplementary Figure 138. The manipulation of *Z*-**3** loaded filter paper with photoswitching and dynamic nucleophilic substitution for varied patterns. (a) 425 nm, 10 s; (b) MeOTf, 5 min; (c) 425 nm, 10 s; (d) 535 nm, 60 s; (e) 425 nm, 10 s; (f) 535 nm, 60 s; (g) 425 nm, 10 s.

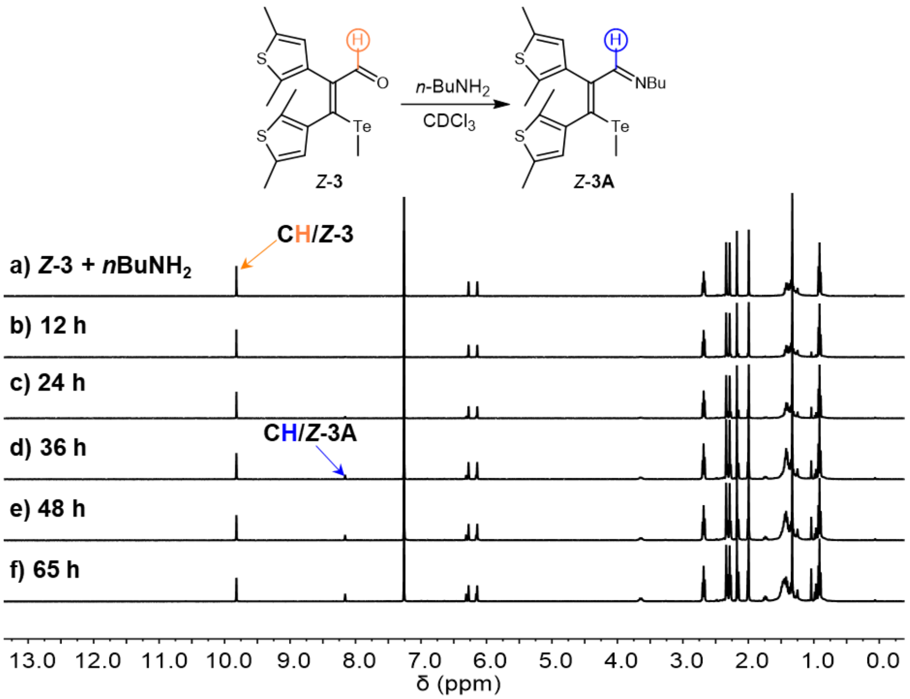

Supplementary Figure 139. ^{1}H NMR spectra of the reaction of *Z*-**3** (10 mM) and 1-butylamine (1.4 equiv.) in $CDCl_3$ at varied time. The yield of imine is 33% after 65 h.

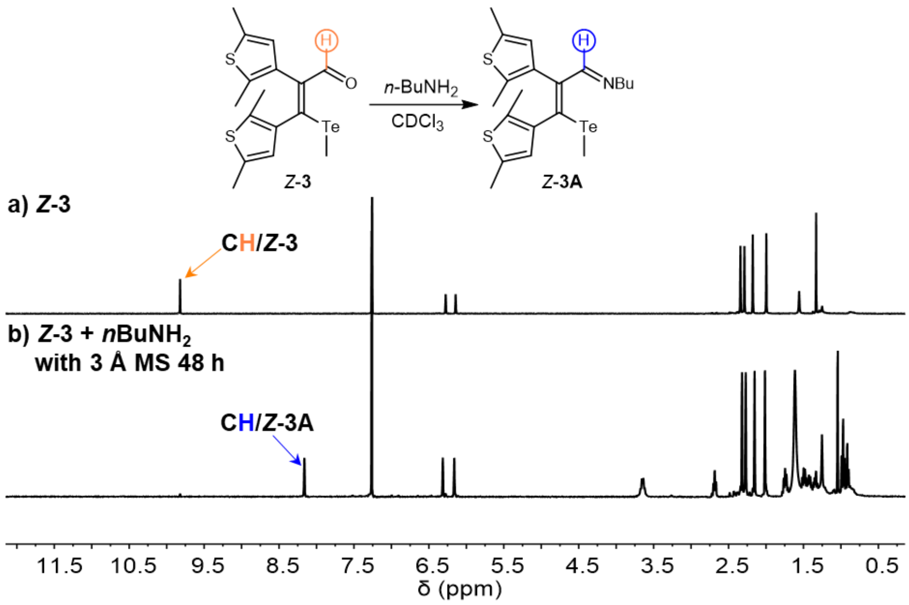

Supplementary Figure 140. ^{1}H NMR spectra of the reaction of *Z*-**3** (10 mM) and 1-butylamine (1.4 equiv.) with 3 Å molecular sieve (MS) in $CDCl_3$. The yield of imine is 95% after 48 h.

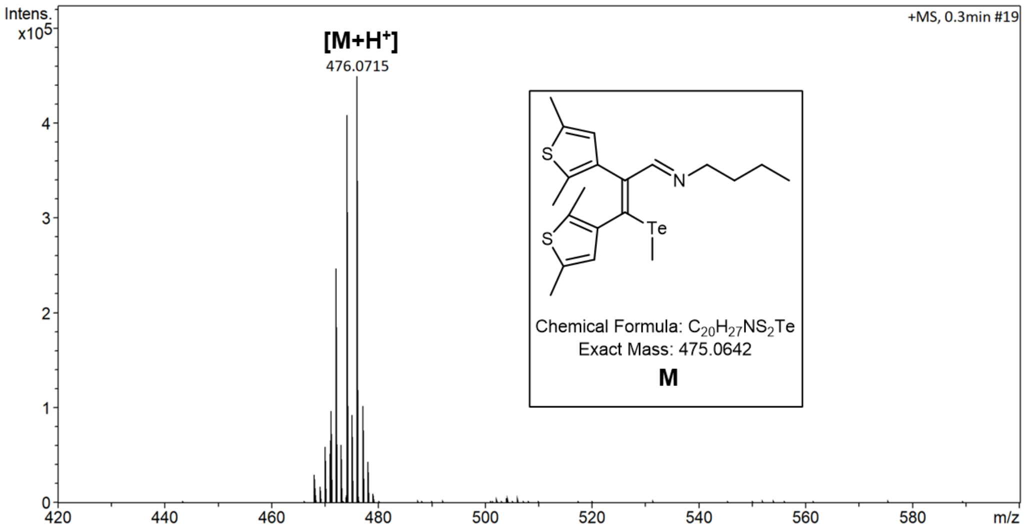

Supplementary Figure 141. ESI-HRMS spectrum of the reaction of *Z*-**3** (10 mM) and 1-butylamine (1.4 equiv.) in $CDCl_3$.

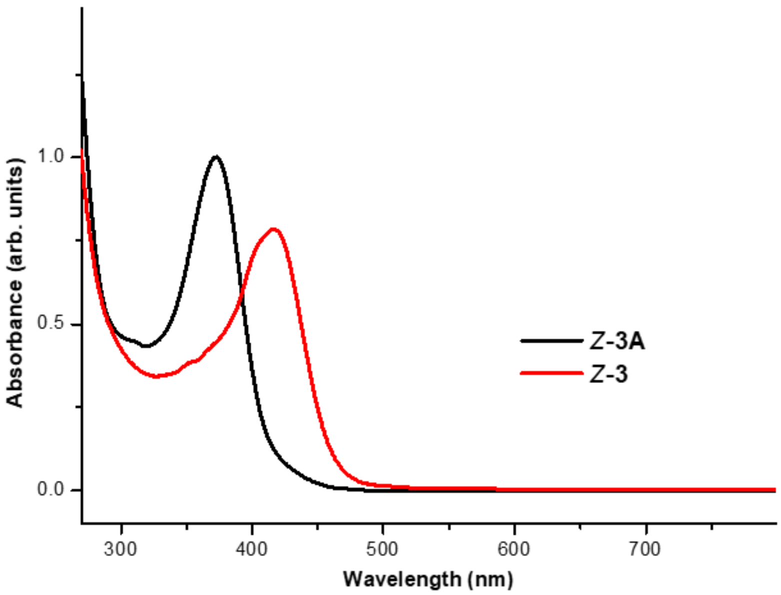

Supplementary Figure 142. UV-vis absorption spectra of *Z*-**3** and *Z*-**3A** in $CHCl_3$.

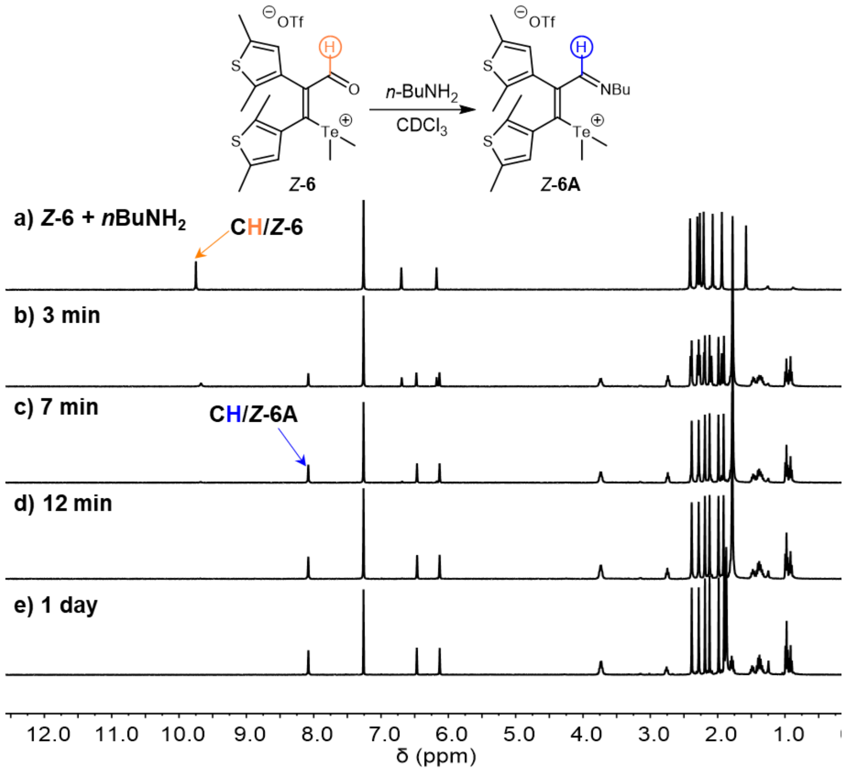

Supplementary Figure 143. ^{1}H NMR spectra of the reaction of *Z*-**6** (10 mM) and 1-butylamine (1.4 equiv.) in $CDCl_3$ at varied time. The yield of imine is 100% after 12 min.

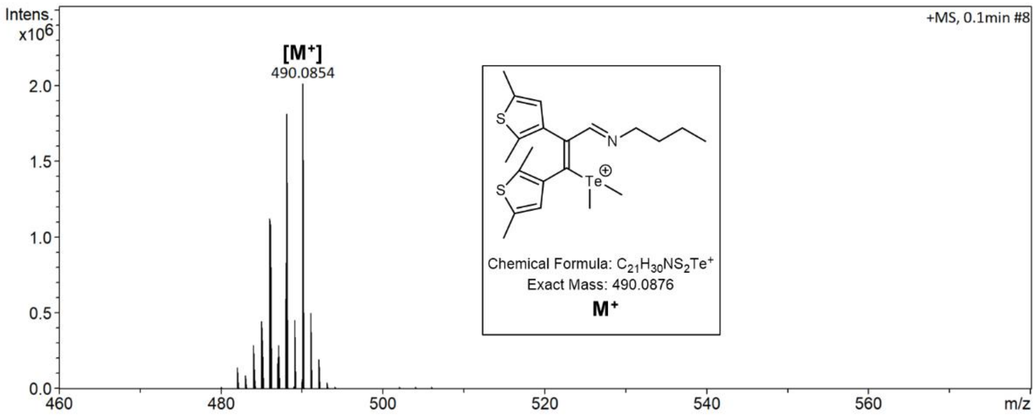

Supplementary Figure 144. ESI-HRMS spectrum of the reaction of *Z*-**6** (10 mM) and 1-butylamine (1.4 equiv.) in $CDCl_3$.

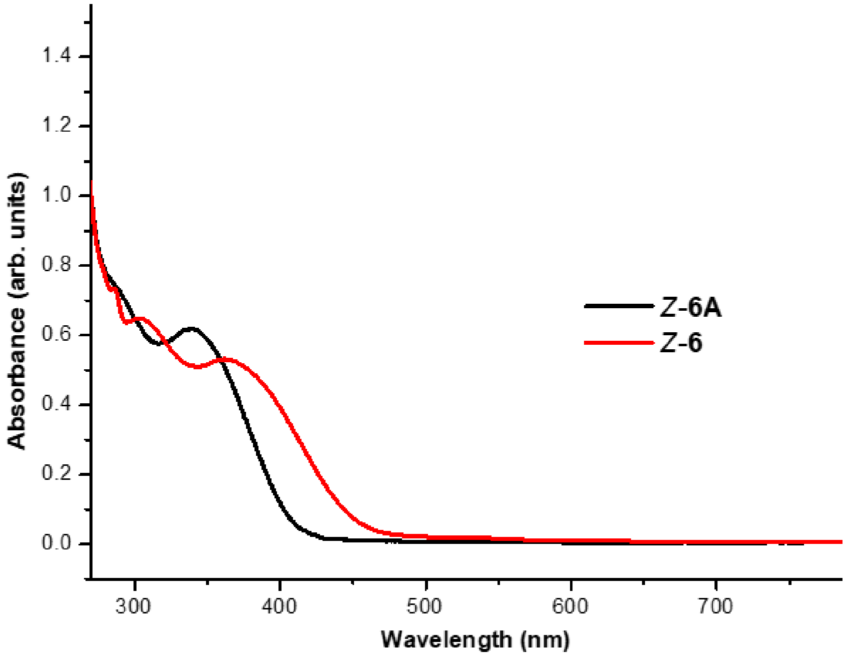

Supplementary Figure 145. UV-vis absorption spectra of *Z*-**6** and *Z*-**6A** in DMSO.

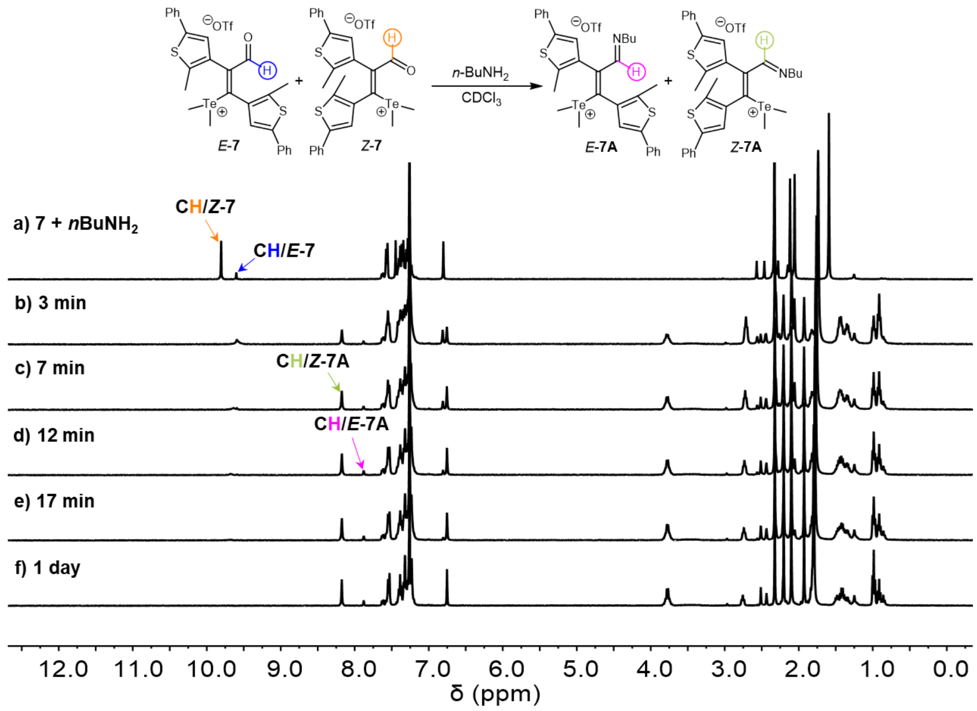

Supplementary Figure 146. ^{1}H NMR spectra of the reaction of **7** (10 mM) and 1-butylamine (1.4 equiv.) in $CDCl_3$ at varied time. The yield of imine is 100% after 17 min.

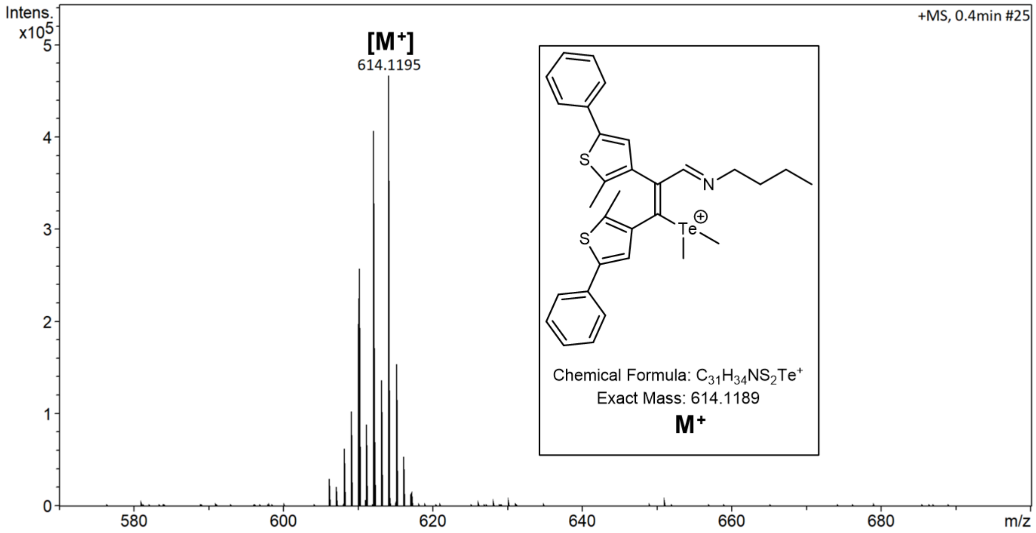

Supplementary Figure 147. ESI-HRMS spectrum of the reaction of **7** (10 mM) and 1-butylamine (1.4 equiv.) in $CDCl_3$.

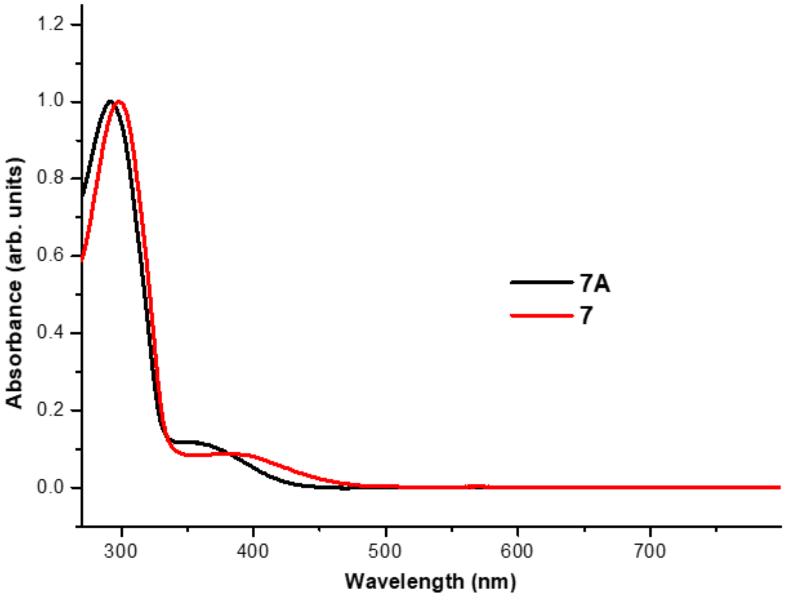

Supplementary Figure 148. UV-vis absorption spectra of **7** and **7A** in DMSO.

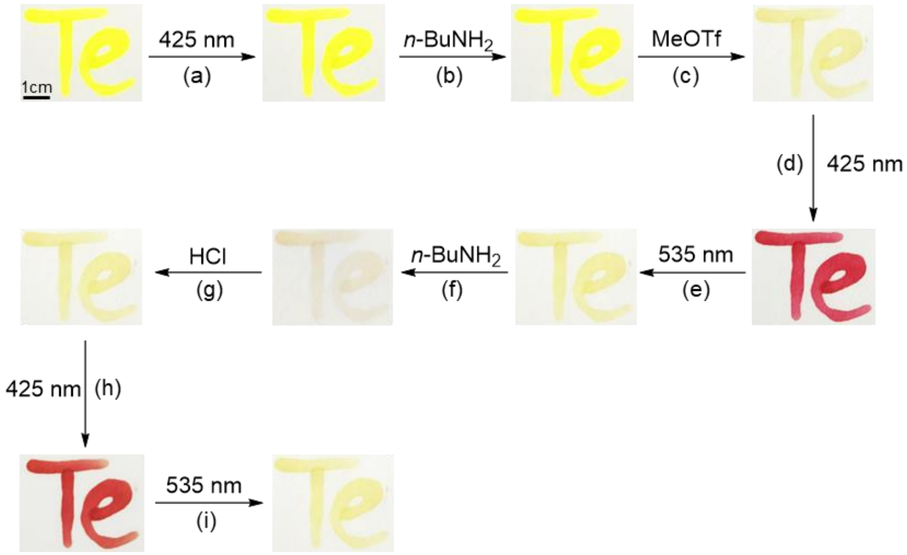

Supplementary Figure 149. The manipulation of writing of Z-**3** (10 mM, $CHCl_3$) as letters "Te" on a filter paper with photoswitching, dynamic nucleophilic substitution, and dynamic imine chemistry. (a) 425 nm, 10 s; (b) aqueous *n*-$BuNH_2$ (40% w/w), 20 s; (c) MeOTf, 5 min; (d) 425 nm, 10 s; (e) 535 nm, 60 s; (f) aqueous *n*-$BuNH_2$ (40% w/w), 20 s; (g) aqueous HCl (30% w/w), 40 s. (h) 425 nm, 10 s; (i) 535 nm, 60 s.

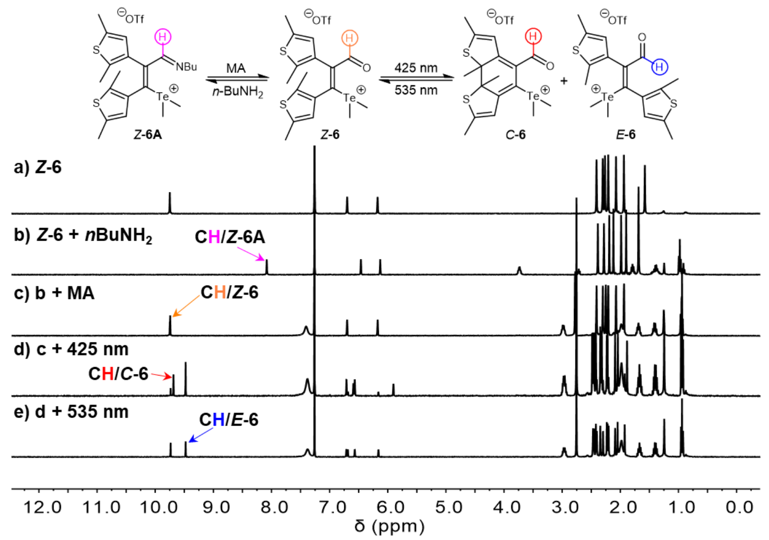

Supplementary Figure 150. (a) ^{1}H NMR spectrum of *Z*-**6** (10 mM) in $CDCl_3$ at 25 °C; (b) ^{1}H NMR spectrum of the reaction of *Z*-**6** (10 mM) and 1-butylamine (1.4 equiv.) in $CDCl_3$ at 25 °C. The yield of *Z*-**6A** is 100%; (c) The addition of MA (2.0 equiv.). The yield of *Z*-**6** is 100%; (d) Further irradiation with visible light (425 nm, 5 min). The ratio of *E*:*Z*:*C* is 52:9:39; (e) Further irradiation with visible light (535 nm, 20 min). The ratio of *E*:*Z* is 52:48.

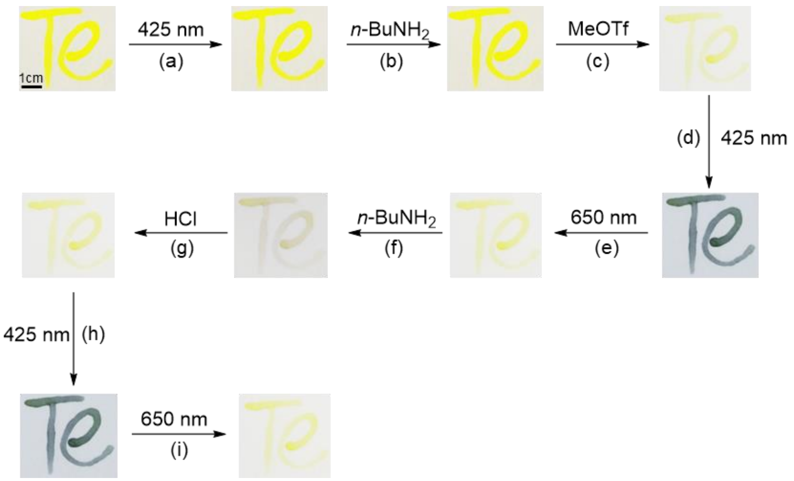

Supplementary Figure 151. The manipulation of writing of Z-**5** (10 mM, $CHCl_3$) as letters "Te" on a filter paper with photoswitching, dynamic nucleophilic substitution, and dynamic imine chemistry. (a) 425 nm, 10 s; (b) aqueous *n*-$BuNH_2$ (40% w/w), 20 s; (c) MeOTf, 10 min; (d) 425 nm, 20 s; (e) 650 nm, 4 min; (f) aqueous *n*-$BuNH_2$ (40% w/w), 30 s; (g) aqueous HCl (30% w/w), 40 s. (h) 425 nm, 20 s; (i) 650 nm, 4 min.

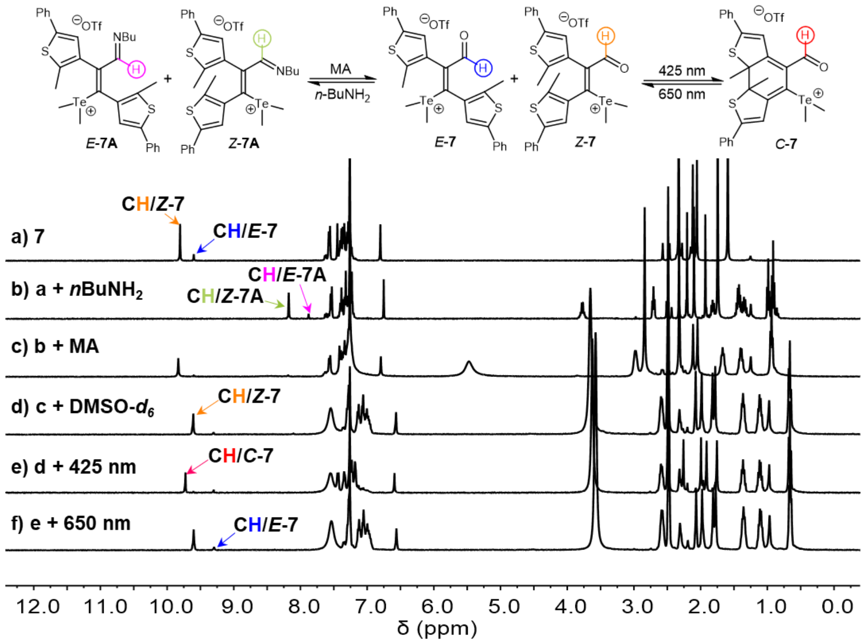

Supplementary Figure 152. (a) ^{1}H NMR spectrum of **7** (*Z*:*E* = 86:14, 10 mM) in $CDCl_3$ at 25 °C; (b) ^{1}H NMR spectrum of the reaction of **7** (10 mM) and 1-butylamine (1.6 equiv.) in $CDCl_3$ (600 μl) at 25 °C. The yield of **7A** is 100%; (c) Further addition of MA (2.2 equiv.). The yield of **7** is 100%; (d) Further addition of DMSO-*d*$_6$ (80 μl); (e) Further irradiation with visible light (425 nm, 50 min). The ratio of *E*:*Z*:*C* is 12:4:84; (f) Further irradiation with visible light (650 nm, 150 min). The ratio of *E*:*Z* is 12:88.

Supplementary Note 8. References

1. Mrozek, T., Görner, H. & Daub, J. Multimode-Photochromism Based on Strongly Coupled Dihydroazulene and Diarylethene. *Chem. - Eur. J.* **7**, 1028-1040 (2001).

2. Teator, A. J. et al. An Isolable, Photoswitchable N-Heterocyclic Carbene: On-Demand Reversible Ammonia Activation. *Angew. Chem., Int. Ed.* **54**, 11559-11563 (2015).

3. (a) Hatchard, C. G. & Parker, C. A. *Proc. Roy. Soc. London, Ser. A* **235**, 518-536 (1956). (b) Montalti, M., Credi, A., Prodi, L., Gandolfi, M. T. in *Handbook of Photochemistry, Third Edition*; CRC Press: Boca Raton, 601-616 (2006).

4. Glaze, A. P., Heller, H. G. & Whittall, J. *J. Chem. Soc. Perk. Trans.* **2**, 591-594 (1992).
