# Supplementary material for: Selection of isomerization pathways of multistep photoswitches by chalcogen bonding
Source: Nat Commun. 2023 Nov 6;14:7139. doi: 10.1038/s41467-023-43013-8 (PMC10628202; doi:10.1038/s41467-023-43013-8)
Supplement: Supplementary file 3 — Description of Additional Supplementary Files [file 41467_2023_43013_MOESM3_ESM.pdf]

Description of Additional Supplementary Files

File name: Supplementary Data 1
Description: Atomic coordinates of the optimized geometries for the studied systems.

File name: Supplementary Data 2
Description: The single-crystal data of *E*-**2**, *E*-**3**, *E*-**4**, *Z*-**4**, *Z*-**5**, and *Z*-**6** are summarized in Supplementary Data 2 and archived at the Cambridge Crystallographic Data Centre under deposition no. CCDC 2249588 (*E*-**4**), 2249589 (*Z*-**4**), 2249590 (*Z*-**5**), 2249591 (*Z*-**6**), 2260917 (*E*-**2**), and 2260918 (*E*-**3**).
